# Inferences on the evolution of the ascorbic acid synthesis pathway in insects using Phylogenetic Tree Collapser (PTC), a tool for the automated collapsing of phylogenetic trees using taxonomic information

**DOI:** 10.1515/jib-2023-0051

**Published:** 2024-07-24

**Authors:** Daniel Glez-Peña, Hugo López-Fernández, Pedro Duque, Cristina P. Vieira, Jorge Vieira

**Affiliations:** ESEI: Escuela Superior de Ingeniería Informática, University of Vigo, Edificio Politécnico, Campus Universitario As Lagoas s/n, 32004, Ourense, Spain; CINBIO: Centro de Investigaciones Biomédicas, University of Vigo, Campus Universitario Lagoas-Marcosende, 36310, Vigo, Spain; SING Research Group, Galicia Sur Health Research Institute (IIS Galicia Sur), SERGAS-UVIGO, 36213 Vigo, Spain; Instituto de Investigação e Inovação em Saúde (I3S), 26706 Universidade do Porto, Rua Alfredo Allen, 208, 4200-135 Porto, Portugal; Instituto de Biologia Molecular e Celular (IBMC), Rua Alfredo Allen, 208, 4200-135 Porto, Portugal; Instituto de Ciências Biomédicas Abel Salazar (ICBAS), 26706 Universidade do Porto, Rua de Jorge Viterbo Ferreira, 228, 4050-313 Porto, Portugal; Faculdade de Ciências da Universidade do Porto (FCUP), Rua do Campo Alegre, s/n, 4169-007 Porto, Portugal

**Keywords:** evolutionary biology, phylogenetic trees, sequence analysis

## Abstract

When inferring the evolution of a gene/gene family, it is advisable to use all available coding sequences (CDS) from as many species genomes as possible in order to infer and date all gene duplications and losses. Nowadays, this means using hundreds or even thousands of CDSs, which makes the inferred phylogenetic trees difficult to visualize and interpret. Therefore, it is useful to have an automated way of collapsing large phylogenetic trees according to a taxonomic term decided by the user (family, class, or order, for instance), in order to highlight the minimal set of sequences that should be used to recapitulate the full history of the gene/gene family being studied at that taxonomic level, that can be refined using additional software. Here we present the Phylogenetic Tree Collapser (PTC) program (https://github.com/pegi3s/phylogenetic-tree-collapser), a flexible tool for automated tree collapsing using taxonomic information, that can be easily used by researchers without a background in informatics, since it only requires the installation of Docker, Podman or Singularity. The utility of PTC is demonstrated by addressing the evolution of the ascorbic acid synthesis pathway in insects. A Docker image is available at Docker Hub (https://hub.docker.com/r/pegi3s/phylogenetic-tree-collapser) with PTC installed and ready-to-run.

## 1 Introduction

The huge amount of genome data that is available today, and that will continue to grow at a faster pace in the near future, can be used to perform comparative analyses and thus, provide a global view on gene/gene family evolution, increase the predictive power of gene function, and identify genes that give each organism its unique characteristics, among others. The use of such large amounts of information implies, however, the capability to process FASTA files in an efficient way and without errors, to align hundreds or even thousands of sequences and to infer gene trees using large datasets. Software is available for all three steps. For instance, SEDA [1] can be used for the efficient processing of a large number of FASTA files. It offers both an easy-to-use graphical interface and a command-line interface with multiple options to deal with gene annotation, sequence retrieval, and FASTA file formatting among others. Clustal Omega [2] (which is also integrated in SEDA) can be used to align hundreds or even thousands of sequences, and FastTree [3] can be used to infer approximately-maximum-likelihood phylogenetic trees using large datasets. Therefore, phylogenies with hundreds of branches can now be routinely obtained, but their visualization and interpretation is difficult due to the large tree size. Moreover, there are other alignment and tree inference methods that perform better than Clustal Omega and FastTree [4, 5], although they cannot feasibly be used to process such large datasets [5, 6]. In this context, we have developed the Phylogenetic Tree Collapser (PTC) software, that performs the automated collapsing of tree branches based on the user-specified taxonomic level (according to the information available at the NCBI Taxonomy database; https://www.ncbi.nlm.nih.gov/taxonomy/), without losing any information regarding the evolution of a given gene/gene family, and provides an additional TSV file with the name of all sequences included in each collapsed group. By doing so, the visualization of the gene tree is improved, which can provide insight into the overall evolution of the gene/gene family, lead to the identification of sequences that fall into unexpected phylogenetic places given known species relationships, likely due to gene miss-annotation, and identify the minimal set of sequences that should be included in further more detailed studies, using more accurate alignment and tree inference algorithms that cannot be used with hundreds or thousands of sequences. For instance, if complex evolutionary scenarios are inferred, it must be shown that such inferences are not dependent on the use of a given alignment or tree reconstruction algorithm. Reconciliation software (reviewed in ref. [7]), known to present as well computational limitations when dealing with large-scale analyses [8], can then be used to correct gene trees and facilitate the identification of gene loss/duplication events [9, 10]. OrthoFinder2 [11], an automated pipeline that can be used to infer orthogroups, genes trees, and gene duplication events, and provides a rooted species tree and extensive comparative genomic statistics, starting from FASTA files, is also computationally expensive and designed for experimental work that might extend beyond the scope of a working hypothesis focused on a small number of genes [12]. Such software can also be prone to errors when dealing with genes with increased rates of evolution [13]. Performing the branch collapsing step manually, using software suites such as MEGA 11 [14], is a time-consuming and error-prone operation. Moreover, the knowledge required to perform the branch collapsing performed by PTC, using, for instance, R scripts is not trivial, and thus such knowledge is not common among life science researchers. PTC can be easily used by researchers without a background in informatics, since it only requires the installation of Docker [15], Podman (https://podman.io/) or Singularity [16]. PTC can also be used to prepare summary figures that show the evolutionary history of the gene/gene family at some taxonomic level, starting from a Newick file where sequences are identified by species names. In summary, PTC is a flexible tool that accepts as input phylogenetic trees in either NEXUS, Newick, Nexml, Phyloxml, or Cdao formats. If all sequence names in the input phylogenetic tree start with the species name (e.g. Drosophila_melanogaster_fruit_fly_Drosophilidae_AAN09306.2), and if sequence groups are to be collapsed at the family level, it does not require any additional files, as PTC can automatically download the taxonomical information and identify the species and family of each sequence in the input tree. Nevertheless, additional files can be provided with custom taxonomies, or that allow the collapsing of the sequence groups to be performed at taxonomic levels other than the family.

PTC has been previously used to simplify a large-scale phylogeny of 2,143 putative bacterial aldonolactone oxireductases coding sequences (CDS) [17], although a descriptive discussion regarding the potential applications of the software was not performed at the time. As such, to further demonstrate the utility of PTC, a study is here presented on the evolution of the genes involved in the synthesis of ascorbic acid (AA), also known as vitamin C or ascorbate, in hexapods. AA is an indispensable antioxidant for normal function and development of eukaryotic cells [18–20]. Many organisms synthesize AA endogenously, but some species such as humans and teleost fish have lost this ability, due to the loss of the *L-gulonolactone oxidase* (*GULO*) gene, involved in the last step of AA synthesis in animals [21, 22]. Although there is no *GULO* gene in *Drosophila melanogaster*, axenic (without microbiome) flies raised on a diet without an AA source present normal AA levels [23, 24]. *De novo* synthesis of AA in *D. melanogaster* must therefore occur, either through a novel pathway or the replacement of GULO by an alternative enzyme.

Recently, it has been suggested that the insect *Bombyx mori*, where *GULO* is missing as well, might synthesize AA through a pathway identical to that of vertebrate species but where GULO is replaced by another enzyme that presents GULO-like activity [25]. Although both are insects, the common ancestor of *D. melanogaster* and *B. mori* lived 290 million years ago [26], and thus it is unclear whether it is logical to expect that these two species synthesize AA using the same pathway. The evolutionary analysis of the genes involved in the animal AA synthesis pathway, as well as of the gene identified in *B. mori* as having GULO-like activity can give an indication on how reasonable such assumption is.

**Figure 1:**
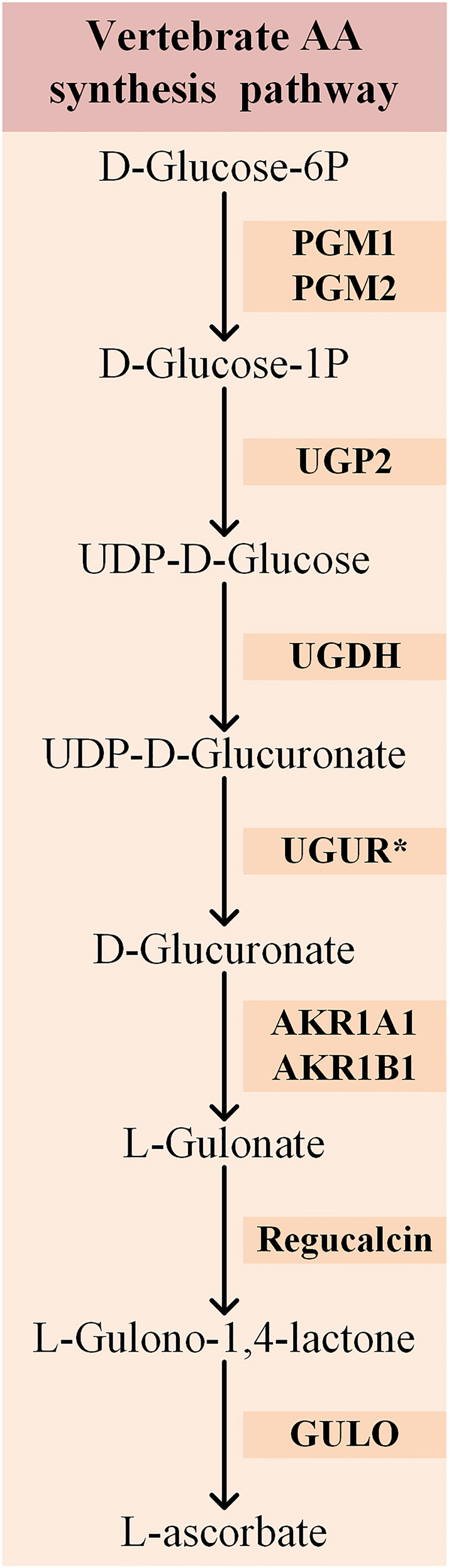
Graphical illustration of the characterized vertebrate AA synthesis pathway, adapted from the MetaCyc database [40]. The enzymes that catalyze each reaction step are shown between the relevant substrates and products. The asterisk highlights the only substrate conversion that remains hypothetical in the literature.

In the characterized vertebrate pathway (Figure 1), D-Glucose is successively converted by Phosphoglucomutases 1 and 2 (PGM1 and PGM2), UDP-Glucose pyrophosphorylase 2 (UGP2), UDP-Glucose 6-dehydrogenase (UGDH), a putative UDP-Glucuronidase (UGUR), the A1/B1 members of the aldo-keto reductase family 1 (AKR1A1/AKR1B1), and the Senescence Marker Protein 30/Regucalcin, to L-Gulonolactone, the substrate of GULO [22, 27], [28], [29]. In humans, PGM1 is responsible for almost 90 % of the total phosphoglucomutase activity, while PGM2 mainly acts as a phosphoribomutase [30–32]. Nevertheless, the two enzymes can perform the conversion of α-D-glucose 6-phosphate to α-D-glucose 1-phosphate, and thus both can play a role in AA synthesis.

Although in vertebrates the *AKR1A1* gene is responsible for approximately 85 % of the D-Glucuronate conversion to L-Gulonate [29], this gene has no homolog in *D. melanogaster* (information available at https://www.ensembl.org; gene ENSG00000117448). Since the *AKR1B1* gene is responsible for the remaining 15 % of the D-Glucuronate conversion in vertebrates [29], and it has a one-to-many homolog relationship relative to *D. melanogaster* (gene ENSG00000085662), it is a prime candidate for the AA synthesis role in this organism. Therefore, here, we infer the evolutionary history of the *PGM1*, *PGM2*, *UGP2*, *UGDH*, *AKR1B1* and the *B. mori* gene showing GULO-like activity (gene *DHCR24*), using CDS from 165 annotated Hexapoda genomes. When performing this study, PTC proved invaluable in the format conversion of NEXUS Bayesian trees, the fast interpretation of gene loss/duplication events, and the easy construction of summary cladograms. Results were obtained in less than 15 min in a task that often takes one day of manual curation in the host lab.

## 2 Materials and methods

### 2.1 PTC implementation

**Figure 2:**
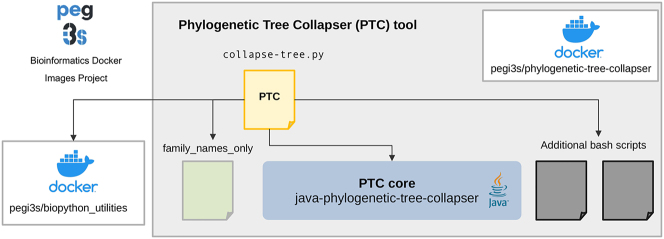
PTC components. The ‘*PTC core*’ is programmed in Java and implements the collapsing procedure explained in Section 2.2. Then, the main PTC command (‘*collapse-tree.py*’), facilitates the invocation of the Java binary by adding some functionalities (e.g. tree format conversion, taxonomy downloading). The main Python scripts uses two additional bash scripts to perform auxiliary work, namely: (1) get_taxonomy.sh, which gets the taxonomy information for each sequence in a given phylogenetic tree file in Newick format; and (2) flatten_taxonomy_using_stop_terms.sh, which flattens a given taxonomy using the stop terms list provided and produces a new taxonomy that will contain only two elements: the first element in the input taxonomy and the element that is present in the stop terms list.

As depicted in Figure 2, PTC is composed of two main components: (*i*) the PTC core, which is programmed in Java 8 using Maven for managing and building the project (https://github.com/sing-group/java-phylogenetic-tree-collapser) and where the collapsing procedure, as explained below, is implemented; (*ii*) the PTC tool, which is mainly implemented as a Python script, using additional scripts, and distributed as an executable Docker image (https://github.com/pegi3s/phylogenetic-tree-collapser). This tool also requires, in some cases, the usage of Docker images from our pegi3s Bioinformatics Docker Images Project (https://pegi3s.github.io/dockerfiles) [33].

The *‘collapse-tree’* command of PTC requires the following input files:

– A phylogenetic tree (--input) in Newick, NEXUS, nexml, phyloxml, or cdao formats (--input-format). If the input file is in a format different to Newick, PTC automatically converts it into Newick and saves it along with the original tree with .nwk extension.
– A tab-delimited file mapping each sequence name to its species (--sequence-mapping). This file must have two columns: the first containing the sequence names of the input tree and the second one containing their corresponding species.
– A plain-text file with the input taxonomy file (--taxonomy). This file must have one line for each species with their taxonomy terms separated by semi-colons.
– A plain-text file with the taxonomy stop terms file (--taxonomy-stop-terms). This file must contain one line for each stop term.

If all sequence names in the input phylogenetic tree start with the species name (e.g. Drosophila_melanogaster_fruit_fly_Drosophilidae_AAN09306.2), then the sequence mapping and taxonomy files are optional. If not provided, the script will retrieve this information from the NCBI Databases using the Entrez utilities. In this case, the files are saved along with the input tree in Newick format with *‘.taxonomy’* and *‘.sequence_to_species_mapping’* extensions so that they can be reused in further analyses.

The taxonomy stop terms file is also optional. If not provided, the script will use as stop terms a list of predefined family names. This file was obtained from NCBI in July 2021 and covers all species.

The output tree (--output) generated with PTC can be a cladogram (--output-type cladogram) or a phylogram (--output-type phylogram) in Newick format. To obtain a phylogram, the input phylogenetic tree must include branch lengths.

The names of the collapsed nodes have the following format: ‘*<common_ancestor>_<group_name>_<number_of_nodes>*’, where *‘<group_name>’* is an auto-incremental index for disambiguation and ’*<number_of_nodes>*’ is the number of nodes under this collapsing. In addition, a tab-delimited file with the collapsed nodes (mapping collapsed node names to sequence names) is generated (--output-collapsed-nodes).

The test cases section of the user manual (https://github.com/pegi3s/phylogenetic-tree-collapser#test-cases) shows different ways of collapsing a sample phylogenetic tree.

### 2.2 PTC collapsing procedure

For each possible pair of sibling nodes, PTC checks if they are collapsible or not based on the taxonomic information and the list of stop terms provided. Two sibling nodes are collapsible if they share a common ancestor in the taxonomy that is below a specified stop term, or the stop term itself, and if it does not result in the inclusion of a species that is already a member of that group. Consider two sequences belonging to *Drosophila_melanogaster* and *Bactrocera_latifrons* species and the following simplified taxonomy:

Bactrocera_latifrons;Tephritidae;Acalyptratae;Schizophora;Brachycera;Diptera;Eukaryota

Drosophila_melanogaster;Drosophilidae;Acalyptratae;Schizophora;Brachycera;Diptera;Eukaryota

If *Drosophilidae* and *Tephritidae* (families) are the stop terms list, then the two sequences are not collapsible. However, if only *Diptera* (an order) is provided as stop term, then they are collapsed because the stop term is shared between the two sequences being considered. In this case, the two nodes are collapsed into a single node, whose name is the one of the lowest common ancestor, *Acalyptratae* in this case. If the input phylogenetic tree includes branch lengths, a weighted average is set as branch of the new collapsed branch: the weight of each branch to be collapsed is the number of leaf nodes under that branch divided by the total of leaf nodes being collapsed. In addition, when several nodes are collapsed into a single one and there are no more siblings, the new node is collapsed with its parent. In this case, the branch length of the resulting node is just the sum of the two branch lengths. This process continues until no more sibling nodes can be collapsed.

As explained above, the name of the collapsed nodes is the one of the lowest common ancestor below the stop terms. In some cases, there is interest in having the stop terms as names of the collapsed nodes (e.g. collapsing a tree using only the family names as stop terms and having these family names in the collapsed tree). To do this, the --flatten-taxonomy-with-stop-terms flag can be used to ask PTC to flatten the taxonomy using the list of stop terms provided before applying the collapsing procedure. In this case, the flattened taxonomy is saved as a file named *‘<input_taxonomy>.flattened_stop_terms’*.

Considering the previous taxonomy of the *Drosophila_melanogaster* and *Bactrocera_latifrons* species, if *Drosophilidae* and *Tephritidae* (families) were used as stop terms, the flattened taxonomy would be:

Bactrocera_latifrons;Tephritidae;

Drosophila_melanogaster;Drosophilidae;

In this case, sequences from the *Tephritidae* family will be collapsed and appear under a node named *‘Tephritidae_<group_name>_<number_of_nodes>’*.

### 2.3 Data preparation and phylogenetic analyses

Sequence datasets were prepared using SEDA [1] and an adaptation of the protocol described in ref. [34]. All CDS from 165 Hexapoda genomes, as well as from *Homo sapiens* and *Mus musculus* were retrieved in FASTA format from the NCBI RefSeq database (last accessed on April 7, 2021). Using SEDA [1], tblastn searches were performed employing as individual queries the human PGM1, PGM2, UGP2, UGDH, AKR1B1 and the *B. mori* GULO-like protein sequences (accession numbers NP_002624.2, NP_060760.2, NP_006750.3, NP_003350.1, NP_001619.1 and XP_004926865.1, respectively). With the exception of the *AKR1B1* dataset, only sequences showing less than 10 % size difference relative to the size of the reference sequence were kept. For *AKR1B1* dataset this value was 2 %, since a higher value leads to the inclusion of a high number of sequences that represent distinct Aldo Keto Reductases with a lower degree of similarity to the query. Two sequences were considered to be isoforms if they share a word that is longer than 50 % of the length of the reference sequence (850, 918, 488, 741, 450 and 759 for *PGM1*, *PGM2*, *UGP2*, *UGDH*, *AKR1B1*, and the *B. mori* gene with *GULO-like* activity, respectively). A Neighbor-Joining phylogeny was produced using MEGA 11 [14], and sequences that clearly did not represent the lineages under study removed (Table S1 in Supplementary File 1).

Using the datasets prepared with SEDA, the phylogenetic analyses described below were performed using Docker images from our pegi3s Bioinformatics Docker Images Project (https://pegi3s.github.io/dockerfiles) [33].

The phylogenetic trees for the *PGM1*, *PGM2*, *UGP2*, *UGDH* and *B. mori* gene with GULO-like activity datasets were produced using the MUSCLE alignment algorithm [35], and using MrBayes [36], two independent runs of 1,500,000 generations, and a defined burn-in of 25 % for the complete analysis, as implemented in the Automatic Detection Of Positively Selected Sites pipeline (ADOPS [37]) Docker image (https://hub.docker.com/r/pegi3s/adops-gui/) were performed. The *UGDH* dataset was subdivided into two smaller ones, according to GC content, given that variation in this parameter prevented convergence.

Due to the high number of sequences present in the *AKR1B1* dataset, it is not feasible to infer Bayesian trees using MrBayes [36], as implemented in ADOPS [37]. Therefore, the *AKR1B1* dataset was aligned using a Docker image for Clustal Omega [2] (https://hub.docker.com/r/pegi3s/clustalomega/), and a tree obtained using a Docker image for FastTree software [3] (https://hub.docker.com/r/pegi3s/fasttree/), using standard parameters. It should be noted that although FastTree is much faster than MrBayes, it is not so accurate [5].

Tree branches were automatically collapsed according to the chosen taxonomic levels using the PTC Docker image here reported. The Newick trees produced by PTC were rooted (the *H. sapiens* and *M. musculus* sequences are the outgroup) using a Docker image for FigTree v1.4.4 software (https://hub.docker.com/r/pegi3s/figtree/).

## 3 Results and discussion

**Figure 3:**
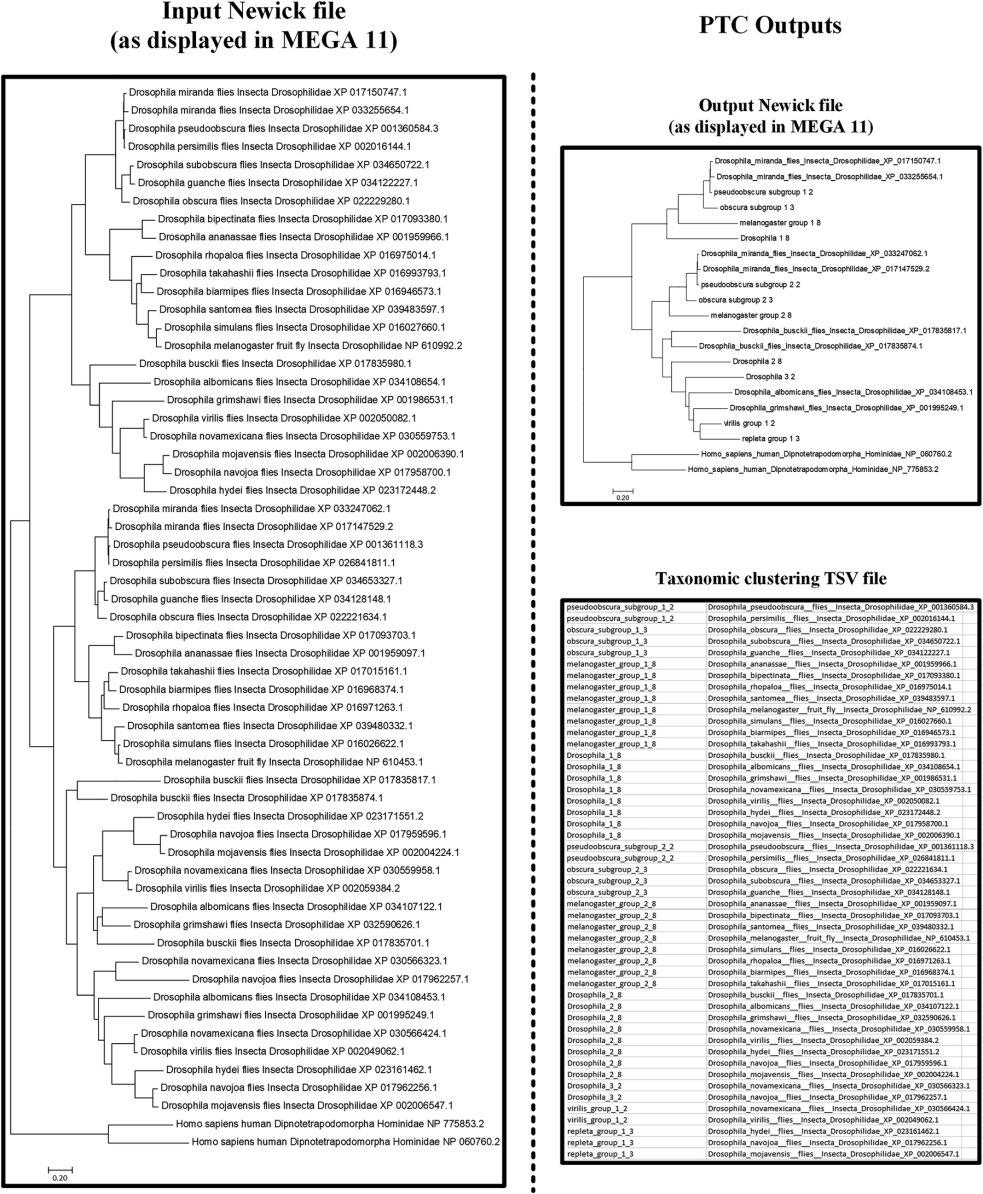
PTC output example when using a subset of *Drosophila PGM2* sequences and the sequences from *Homo sapiens* (as outgroup), using the family taxonomic level as stop term (default protocol). Using a compatible input file (in this case in Newick format), PTC collapses the sequences present in each phylogenetic cluster at the lowest taxonomic level possible. In the collapsed tree, the numbers after the taxonomic group names indicate the number of sequences contained within each simplified branch. An additional TSV file created after the taxonomic collapsing operation indicates the names of the sequences included in each group.

PTC allows the collapsing of phylogenetic trees at any chosen taxonomic level, which greatly simplifies the manual comparison of inferred gene trees with species phylogenies reported in the literature or websites such as “The Tree of Life Web Project” (http://tolweb.org/tree/; [38]), and ultimately facilitates the interpretation of results by the user. A small example of the use of PTC is shown in Figure 3. It also allows the identification of the minimal set of sequences that should be included in further more detailed studies without implying loss of information, using additional software. Moreover, in the case of single copy genes, the ability of PTC to collapse branches according to the chosen taxonomic groups simplifies the creation of summary phylograms/cladograms, and thus of species trees, that after minor editing can be used for publication purposes.

By using PTC, the interpretation of the evolutionary history of the genes putatively involved in the AA synthesis in *B. mori* was addressed in order to understand whether a similar pathway could be used by other insect species. Two approaches were used, namely i) restraining the branch collapsing to the chosen taxonomic level, in this case the order, using a “Stop terms” input file in addition to the phylogeny (Custom protocol). By doing so, an overview of the evolutionary history of the gene being studied is obtained; ii) branch collapsing based on the taxonomy information present in NCBI, using only the phylogenies as input (Default protocol). The stop term file and a file describing the commands used (Supplementary Files 2 and 3, respectively), together with the original Newick/Nexus trees (Supplementary Files 4–10) can be used to reproduce these analyses. The rooted collapsed phylogenies are also available as supplementary material (Figures S1–S14 in Supplementary File 1).

By collapsing sequences from related taxonomic groups at the lowest taxonomic rank, as long as it is below or equal to the family level, miss-interpretations are avoided, such as, claiming that all species from a given family have two copies of a given gene, when all evidence comes from species of a single genus, for instance. For both cases, the number of tree branches was significantly reduced (Table 1).

**Table 1:** Progressive collapse of the analyzed phylogenetic trees using PTC.

Gene	Original tree branches	Default protocol tree branches	Custom protocol tree branches
*PGM1*	157	65	12
*PGM2*	220	86	57
*UGP2*	159	63	30
*UGDH*	137/36^a^	67/19^a^	43/16^a^
*AKR1B1*	685	346	269
*DHCR24*	93	69	38

^a^Numbers for the two phylogenies created due to variable GC content issues in the initial dataset.

**Figure 4:**
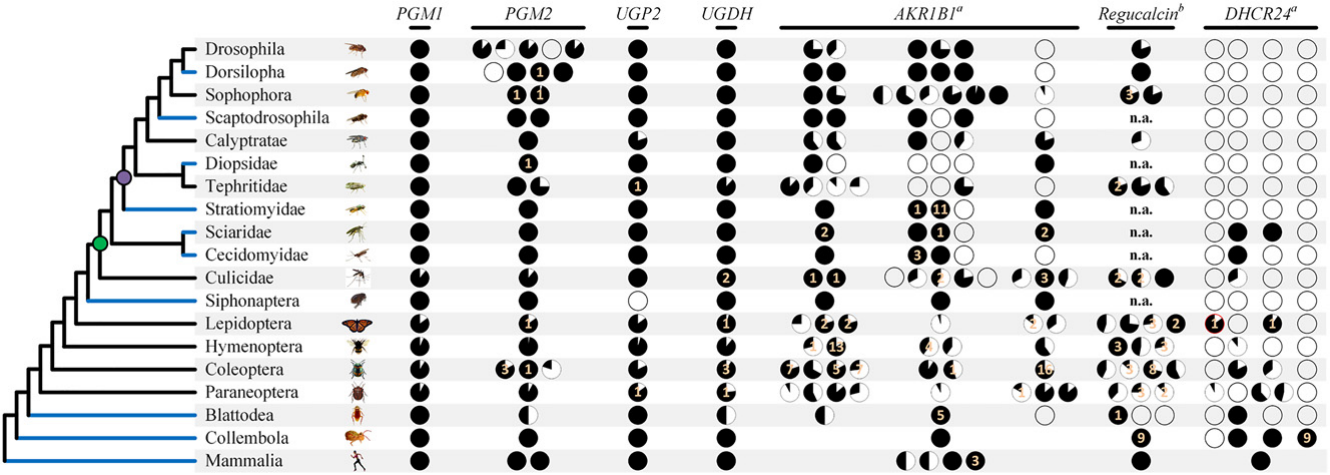
Summary of the presence/absence patterns of the analyzed genes within the Hexapoda. The circles represent the total number of inferred homologs for each lineage in the cladogram, and the circles’ fill indicates the number of species in which the given gene was identified relative to the total number of species analyzed. The orange numbers on top of circles represent inferred specific gene duplications found within the lineages. The red color outline within the *DHCR24* Lepidoptera section of the image represents the *B. mori DHCR24* gene lineage. The taxonomic groups represented by less than three species are highlighted by blue branches in the cladogram. The green and purple circles included in the cladogram indicate the start of the Diptera order and Brachycera suborder, respectively. Taxonomic relationships are depicted as seen in refs. [38, 41], [42], [43]. The sections marked with “a” represent genes with inferred ancestral duplicates separated by distinct columns from gene 1 (left side) up to gene 4 (right side). The information present in “b” was adapted from [44]. CDS from *Clunio marinus* (Chironomidae) were not available in the RefSeq dataset used in these analyses, and as such the results regarding this species presented in the original report are not represented in the figure. Some of the species analyzed in this work were not available in ref. [44], and as such some groups are represented as “not available” (n.a.). Table S2 (see Supplementary File 1) shows the name of the fly homologs of the human genes here analyzed.

Figure 4 shows the summary of the analyses here performed. The gene identified in *B. mori* by Hou et al. as having GULO-like activity [25] is present as a single copy in humans (Gene *DHCR24*). There are, however, four Hexapoda gene lineages, here labeled as GL1 to GL4. The reported *B. mori* gene with GULO-like activity belongs to GL1. This gene lineage was lost in the majority of the taxonomic groups analyzed, being only present in the Lepidoptera and Paraneoptera orders. GL2 is also only present in a reduced number of Hymenoptera and basal Diptera families. The Coleoptera, Blattodea and Collembola. GL3 gene is only present in the Diptera, Lepidoptera, Coleoptera, Paraneoptera and Collembola. Within the Paraneoptera Aphididae family, the ancestral gene of the GL3 lineage was duplicated. GL4 is only present in Collembola, and appears to have been lost in the Insecta lineage. Therefore, there are no homologs of the *B. mori* gene with GULO-like activity in the dipteran Brachycera suborder, where *D. melanogaster* is included. As such, AA is not synthesized by the same pathway in *Drosophila* and *Bombyx*. It should be noted, that when a lineage is represented by less than three species, gene losses cannot be confidently inferred, since most genomes are not fully sequenced and genes are not always correctly annotated [39], but this is not the case.

Since AA is not synthesized by the same pathway in *Drosophila* and *Bombyx*, the evolutionary history of the other genes putatively involved in the *B. mori* pathway could be informative. Indeed, the *GULO* gene was lost before the separation of crustaceans and insects, an event inferred to have happened more than 520 million years ago [26]. Therefore, the constraints acting on the genes previously involved in the synthesis of AA may have changed. Moreover, such constraints may have changed again in the lineage leading to Lepidoptera, when the ability to synthesize AA was reacquired. Such changes in constrains may have allowed gene duplications or deletions that were previously not allowed.

*PGM1* is always present as a single copy gene, while *PGM2* appears to have been duplicated once in the common ancestor of the dipteran Drosophilidae family, and further duplicated in the Drosophila and Dorsilopha subgenera, but not in the Sophophora and Scaptodrosophila groups. An ancestral duplication also appears to have happened within the Tephritidae family and in the common ancestor of the coleopteran Cerambycidae, Chrysomelidae and Curculionidae groups (Figure 4). Having no homologs of the *B. mori* gene with GULO-like activity seems to be associated with a higher propensity to duplicate the *PGM2* gene. Whether this gene is involved in the reacquisition of AA synthesis in the dipteran Brachycera lineage must now be tested on the bench.

*UGP2* is mainly a single copy gene in Hexapoda, although it can be found duplicated in the dipteran *Rhagoletis* genus and in the Paraneoptera *Acyrthosiphon pisum* and *Thrips palmi* species (Figure 4).

*UGDH* gene is also mainly present as a single copy gene, but gene duplications can be found in few species from Diptera (family Culicidae), Lepidoptera, Coleoptera and Paraneoptera (Figure 4).

The interpretation of the *AKR1B1* phylogeny does not fully rely only on the inferred scenario, since many sequence relationships show little or no support. Overall, it was possible to infer three main *AKR1B1* duplicates derived from a single copy in the common ancestor of the Hexapoda (henceforth referred as *Gene 1* to *Gene 3*). *Gene 1* has a high prevalence of conserved duplicates in many of the major Hexapoda lineages possibly derived from ancestral duplications within the Diptera, Lepidoptera, Hymenoptera and Coleoptera orders. The scenario is similar in *Gene 2*, where this phenomenon can be observed within the Diptera, Hymenoptera and Coleoptera orders. *Gene 3* has a less complex duplication pattern, as the most ancestral duplication observed only affected the lepidopteran species. Although as an aggregate the three *AKR1B1* duplicates are spread across all the Hexapoda species analyzed, individually they display a few independent loss events (Figure 4).

## 4 Conclusions

By collapsing branches that do not provide additional information, while retaining information on all gene duplications that occurred during evolution, the PTC program here presented greatly facilitated the interpretation of phylogenetic analyses here performed, involving CDSs from 165 Hexapoda annotated genomes and two other genomes used as an outgroup. These analyses showed that AA synthesis does not occur through the same pathway in *Bombyx* and *Drosophila*. Contrary to the remaining genes that could be involved in AA synthesis in *Drosophila*, the gene that is postulated to replace *GULO* in *B. mori* has lost all homologs in the dipteran Brachycera lineage, and such event seems to be associated with a higher propensity to duplicate the *PGM2* gene. This could be an indication of the involvement of this gene in the reacquisition of AA synthesis in this lineage.

## Supplementary Material

Supplementary Material Details
