# Supplementary material for: Inferences on the evolution of the ascorbic acid synthesis pathway in insects using Phylogenetic Tree Collapser (PTC), a tool for the automated collapsing of phylogenetic trees using taxonomic information
Source: J Integr Bioinform. 2024 Jul 24;21(2):20230051. doi: 10.1515/jib-2023-0051 (PMC11377030; doi:10.1515/jib-2023-0051)
Supplement: Supplementary file 1 — Supplementary Material Details [file j_jib-2023-0051_suppl_001.zip › Supplementary_File_1.pdf]

Inferences on the evolution of the ascorbic acid synthesis pathway in insects using Phylogenetic Tree Collapser (PTC), a tool for the automated collapsing of phylogenetic trees using taxonomic information

Daniel Glez-Peña, Hugo López-Fernández, Pedro Duque, Cristina P. Vieira, and Jorge Vieira

Supplementary tables

Table S1 – Sequences removed from the original datasets after Neighbor-joining verification.
Table S2 – *D. melanogaster* gene homologs obtained from the phylogenetic analyses.

Supplementary figures

Figure S1 – *PGM1* phylogeny after the PTC Custom protocol.
Figure S2 – *PGM1* phylogeny after the PTC Default protocol.
Figure S3 – *PGM2* phylogeny after the PTC Custom protocol.
Figure S4 – *PGM2* phylogeny after the PTC Default protocol.
Figure S5 – *UGP2* phylogeny after the PTC Custom protocol.
Figure S6 – *UGP2* phylogeny after the PTC Default protocol.
Figure S7 – Main *UGDH* phylogeny after the PTC Custom protocol.
Figure S8 – Main *UGDH* phylogeny after the PTC Default protocol.
Figure S9 – Secondary *UGDH* phylogeny after the PTC Custom protocol.
Figure S10 – Secondary *UGDH* phylogeny after the PTC Default protocol.
Figure S11 – *AKR1B1* phylogeny after the PTC Custom protocol.
Figure S12 – *AKR1B1* phylogeny after the PTC Default protocol.
Figure S13 – *DHCR24* phylogeny after the PTC Custom protocol.
Figure S14 – *DHCR24* phylogeny after the PTC Default protocol.

Table S1

PGM1	*PGM2*	*UGDH*	*UGP2*	*DHCR24*
Bombyx_mori_(domestic_silkworm)_Insecta_Bombycidae_XP_004929437.1	Drosophila_simulans_(flies)_Insecta_Drosophilidae_XP_016032127.1	Rhagoletis_zephyria_(snowberry_fruit_fly)_Insecta_Tephritidae_XP_017477891.1	Aethina_tumida_(small_hive_beetle)_Insecta_XP_019881380.1	Amyelois_transitella_(moths)_Lepidoptera_Pyralidae_XP_013188145.1
Bombyx_mandarina_(wild_silkworm)_Insecta_Bombycidae_XP_028030827.1	Drosophila_sechellia_(flies)_Insecta_Drosophilidae_XP_032573482.1	Rhagoletis_zephyria_(snowberry_fruit_fly)_Insecta_Tephritidae_XP_017493009.1	Papilio_xuthus_(Asian_swallowtail)_Insecta_Papilionidae_XP_013163759.1	Galleria_mellonella_(greater_wax_moth)_Lepidoptera_Pyralidae_XP_026751344.1
Manduca_sexta_(tobacco_hornworm)_Insecta_Sphingidae_XP_037302089.1	Drosophila_mauritiana_(flies)_Insecta_Drosophilidae_XP_033157475.1		Papilio_xuthus_(Asian_swallowtail)_Insecta_Papilionidae_XP_013162454.1	Apis_laboriosa_(Himalayan_honeybee)_Hymenoptera_Apidae_XP_043803556.1
Danaus_plexippus_plexippus_(monarch_butterfly)_Insecta_Nymphalidae_XP_032515592.1	Drosophila_melanogaster_(fruit_fly)_Insecta_Drosophilidae_NP_524675.1		Diabrotica_virgifera_virgifera_(western_corn_rootworm)_Insecta_Chrysomelidae_XP_028145235.1	Chrysoperla_carnea_(insects)_XP_044730725.1
Vanessa_tameamea_(butterflies)_Insecta_Nymphalidae_XP_026489474.1	Drosophila_erecta_(flies)_Insecta_Drosophilidae_XP_001973181.1		Fopius_arisanus_(wasps,_ants,_and_bees)_Insecta_Braconidae_XP_011305910.1	Orussus_abietinus_(hymenopterans)_Hymenoptera_Orussidae_XP_012281185.1
Helicoverpa_armigera_(cotton_bollworm)_Insecta_Noctuidae_XP_021194330.1	Drosophila_santomea_(flies)_Insecta_Drosophilidae_XP_039484920.1		Trichogramma_pretiosum_(wasps,_ants,_and_bees)_Insecta_Trichogrammatidae_XP_014227887.1	Anoplophora_glabripennis_(Asian_longhorned_beetle)_Coleoptera_Cerambycidae_XP_018569933.1
Papilio_polytes_(common_Mormon)_Insecta_Papilionidae_XP_013149198.1	Drosophila_yakuba_(flies)_Insecta_Drosophilidae_XP_002095770.2		Sitophilus_oryzae_(rice_weevil)_Insecta_Curculionidae_XP_030747353.1	Apis_cerana_(Asiatic_honeybee)_Hymenoptera_Apidae_XP_016921488.1
Papilio_machaon_(common_yellow_swallowtail)_Insecta_Papilionidae_XP_014369384.1	Drosophila_elegans_(flies)_Insecta_Drosophilidae_XP_017127571.1		Sitophilus_oryzae_(rice_weevil)_Insecta_Curculionidae_XP_030755882.1	Melanaphis_sacchari_(aphids)_Hemiptera_Aphididae_XP_025193263.1
Pieris_rapae_XP_022123915.1	Drosophila_rhopaloa_(flies)_Insecta_Drosophilidae_XP_016971773.1		Maniola_hyperantus_(ringlet)_Insecta_XP_034838984.1	Onthophagus_taurus_(beetles)_Coleoptera_Scarabaeidae_XP_022919082.1
Teleopsis_dalmanni_(flies)_Insecta_Diopsidae_XP_037929741.1	Drosophila_takahashii_(flies)_Insecta_Drosophilidae_XP_017015766.1		Anopheles_coluzzii_(mosquitos)_Insecta_Culicidae_XP_040236446.1	Cryptotermes_secundus_(termites)_Blattodea_Kalotermitidae_XP_023713618.1
Teleopsis_dalmanni_(flies)_Insecta_Diopsidae_XP_037949477.1	Drosophila_biarmipes_(flies)_Insecta_Drosophilidae_XP_016966257.1		Acyrthosiphon_pisum_(pea_aphid)_Insecta_Aphididae_XP_001944680.1	Zootermopsis_nevadensis_(termites)_Blattodea_Termopsidae_XP_021937079.1
Cimex_lectularius_(bed_bug)_Insecta_Cimicidae_XP_014251917.1	Drosophila_subpulchrella_(flies)_Insecta_Drosophilidae_XP_037721855.1		Agrilus_planipennis_(emerald_ash_borer)_Insecta_Buprestidae_XP_018322656.1	
Stomoxys_calcitrans_(stable_fly)_Insecta_Muscidae_XP_013104900.1	Drosophila_suzukii_(flies)_Insecta_Drosophilidae_XP_016926671.2		Anoplophora_glabripennis_(Asian_longhorned_beetle)_Insecta_Cerambycidae_XP_018578897.1	
Lucilia_cuprina_(Australian_sheep_blowfly)_Insecta_Calliphoridae_XP_023307068.1	Drosophila_kikkawai_(flies)_Insecta_Drosophilidae_XP_017028155.1		Anoplophora_glabripennis_(Asian_longhorned_beetle)_Insecta_Cerambycidae_XP_023313065.1	
Lucilia_sericata_(common_green_bottle_fly)_Insecta_Calliphoridae_XP_037815336.1	Drosophila_serrata_(flies)_Insecta_Drosophilidae_XP_020817122.1		Anopheles_albimanus_(mosquitos)_Insecta_Culicidae_XP_035773908.1	
Nicrophorus_vespilloides_(beetles)_Insecta_Silphidae_XP_017781760.1	Drosophila_eugracilis_(flies)_Insecta_Drosophilidae_XP_017081105.1		Apis_cerana_(Asiatic_honeybee)_Insecta_Apidae_XP_016909221.1	
Cimex_lectularius_(bed_bug)_Insecta_Cimicidae_XP_014244419.1	Drosophila_bipectinata_(flies)_Insecta_Drosophilidae_XP_017098422.1		Hyposmocoma_kahamanoa_(moths)_Insecta_Cosmopterigidae_XP_026317391.1	
Drosophila_eugracilis_(flies)_Insecta_Drosophilidae_XP_017069549.1	Drosophila_ananassae_(flies)_Insecta_Drosophilidae_XP_001957236.1		Melanaphis_sacchari_(aphids)_Insecta_Aphididae_XP_025194846.1	
Homo_sapiens_human_Mammalia_Hominidae_NP_068800.2	Drosophila_persimilis_(flies)_Insecta_Drosophilidae_XP_002024975.1		Nicrophorus_vespilloides_(beetles)_Insecta_Silphidae_XP_017769985.1	
Mus_musculus_house_mouse_Mammalia_Muridae_NP_778178.3	Drosophila_subobscura_(flies)_Insecta_Drosophilidae_XP_034655421.1		Bradysia_coprophila_(flies)_Insecta_Sciaridae_XP_037027705.1	
	Drosophila_virilis_(flies)_Insecta_Drosophilidae_XP_002046538.1		Anopheles_stephensi_(Asian_malaria_mosquito)_Insecta_Culicidae_XP_035916578.1	
	Drosophila_novamexicana_(flies)_Insecta_Drosophilidae_XP_030567850.1		Copidosoma_floridanum_(wasps,_ants,_and_bees)_Insecta_Encyrtidae_XP_014214929.1	
	Drosophila_arizonae_(flies)_Insecta_Drosophilidae_XP_017863552.1		Eufriesea_mexicana_(bees)_Insecta_Apidae_XP_017760787.1	
	Drosophila_mojavensis_(flies)_Insecta_Drosophilidae_XP_002011961.1		Aedes_aegypti_(yellow_fever_mosquito)_Insecta_Culicidae_XP_001659746.2	
	Drosophila_grimshawi_(flies)_Insecta_Drosophilidae_XP_001984409.1		Danaus_plexippus_plexippus_(monarch_butterfly)_Insecta_XP_032528285.1	
	Drosophila_busckii_(flies)_Insecta_Drosophilidae_XP_017844450.1		Chelonus_insularis_(wasps,_ants,_and_bees)_Insecta_Braconidae_XP_034935842.1	
	Drosophila_willistoni_(flies)_Insecta_Drosophilidae_XP_002062067.1		Rhopalosiphum_maidis_(corn_leaf_aphid)_Insecta_Aphididae_XP_026821945.1	
	Rhagoletis_pomonella_(apple_maggot)_Insecta_Tephritidae_XP_036338294.1		Bombus_bifarius_(bees)_Insecta_Apidae_XP_033313127.1	
	Rhagoletis_zephyria_(snowberry_fruit_fly)_Insecta_Tephritidae_XP_017477828.1		Nylanderia_fulva_(ants)_Insecta_Formicidae_XP_029158035.1	
	Bactrocera_oleae_(olive_fruit_fly)_Insecta_Tephritidae_XP_014098751.1		Bombus_impatiens_(common_eastern_bumble_bee)_Insecta_Apidae_XP_003487879.1	
	Musca_domestica_(house_fly)_Insecta_Muscidae_XP_005188107.1		Bicyclus_anynana_(squinting_bush_brown)_Insecta_XP_023949028.1	
	Stomoxys_calcitrans_(stable_fly)_Insecta_Muscidae_XP_013103995.1		Leptinotarsa_decemlineata_(Colorado_potato_beetle)_Insecta_Chrysomelidae_XP_023022882.1	
	Lucilia_sericata_(common_green_bottle_fly)_Insecta_Calliphoridae_XP_037817741.1		Leptinotarsa_decemlineata_(Colorado_potato_beetle)_Insecta_Chrysomelidae_XP_023024179.1	
	Lucilia_cuprina_(Australian_sheep_blowfly)_Insecta_Calliphoridae_XP_023300726.1		Dendroctonus_ponderosae_(mountain_pine_beetle)_Insecta_Curculionidae_XP_019753639.1	
	Anopheles_gambiae_str._PEST_(African_malaria_mosquito)_Insecta_Culicidae_XP_315885.2		Dendroctonus_ponderosae_(mountain_pine_beetle)_Insecta_Curculionidae_XP_019763712.1	
	Hermetia_illucens_(flies)_Insecta_Stratiomyidae_XP_037907221.1		Anopheles_arabiensis_(mosquitos)_Insecta_Culicidae_XP_040166189.1	
	Contarinia_nasturtii_(swede_midge)_Insecta_Cecidomyiidae_XP_031638990.1		Contarinia_nasturtii_(swede_midge)_Insecta_Cecidomyiidae_XP_031627197.1	
	Bradysia_coprophila_(flies)_Insecta_Sciaridae_XP_037039073.1		Bombus_terrestris_(buff-tailed_bumblebee)_Insecta_Apidae_XP_003399057.1	
	Anoplophora_glabripennis_(Asian_longhorned_beetle)_Insecta_Cerambycidae_XP_023309822.1		Aphis_gossypii_(cotton_aphid)_Insecta_Aphididae_XP_027837611.1	
	Diabrotica_virgifera_virgifera_(western_corn_rootworm)_Insecta_Chrysomelidae_XP_028143837.1		Microplitis_demolitor_(wasps,_ants,_and_bees)_Insecta_Braconidae_XP_008558283.1	
	Sitophilus_oryzae_(rice_weevil)_Insecta_Curculionidae_XP_030749056.1		Photinus_pyralis_(common_eastern_firefly)_Insecta_Lampyridae_XP_031344850.1	
	Microplitis_demolitor_(wasps,_ants,_and_bees)_Insecta_Braconidae_XP_014296482.1		Photinus_pyralis_(common_eastern_firefly)_Insecta_Lampyridae_XP_031345222.1	
	Nasonia_vitripennis_(jewel_wasp)_Insecta_Pteromalidae_XP_001608147.1		Folsomia_candida_(springtails)_Collembola_Isotomidae_XP_035709198.1	
	Belonocnema_treatae_(wasps,_ants,_and_bees)_Insecta_Cynipidae_XP_033214709.1		Orussus_abietinus_(hymenopterans)_Insecta_Orussidae_XP_023289624.1	
	Acromyrmex_echinatior_(Panamanian_leafcutter_ant)_Insecta_Formicidae_XP_011055210.1		Apis_florea_(little_honeybee)_Insecta_Apidae_XP_003691874.1	
	Atta_cephalotes_(ants)_Insecta_Formicidae_XP_012062937.1		Diachasma_alloeum_(wasps,_ants,_and_bees)_Insecta_Braconidae_XP_015120680.1	
	Atta_colombica_(ants)_Insecta_Formicidae_XP_018044394.1		Nilaparvata_lugens_(brown_planthopper)_Insecta_Delphacidae_XP_022189563.1	
	Cyphomyrmex_costatus_(ants)_Insecta_Formicidae_XP_018405593.1		Culex_pipiens_pallens_(northern_house_mosquito)_Insecta_Culicidae_XP_039438692.1	
	Harpegnathos_saltator_(Jerdon's_jumping_ant)_Insecta_Formicidae_XP_011140815.1		Bombus_vancouverensis_nearcticus_(bees)_Insecta_Apidae_XP_033189516.1	
	Megachile_rotundata_(alfalfa_leafcutting_bee)_Insecta_Megachilidae_XP_003699636.1		Myzus_persicae_(green_peach_aphid)_Insecta_Aphididae_XP_022183731.1	
	Bombus_terrestris_(buff-tailed_bumblebee)_Insecta_Apidae_XP_003401536.1		Bemisia_tabaci_(sweet_potato_whitefly)_Insecta_Aleyrodidae_XP_018902053.1	
	Bombus_vosnesenskii_(bees)_Insecta_Apidae_XP_033344194.1		Aethina_tumida_(small_hive_beetle)_Insecta_XP_019879559.1	
	Bombus_impatiens_(common_eastern_bumble_bee)_Insecta_Apidae_XP_012247316.1		Nasonia_vitripennis_(jewel_wasp)_Insecta_Pteromalidae_XP_001602623.1	
	Bombus_vancouverensis_nearcticus_(bees)_Insecta_Apidae_XP_033191144.1		Sipha_flava_(yellow_sugarcane_aphid)_Insecta_Aphididae_XP_025420849.1	
	Bombus_bifarius_(bees)_Insecta_Apidae_XP_033300165.1		Drosophila_grimshawi_(flies)_Insecta_Drosophilidae_XP_001993128.2	
	Folsomia_candida_(springtails)_Collembola_Isotomidae_XP_035709459.1		Tribolium_castaneum_(red_flour_beetle)_Insecta_Tenebrionidae_NP_001164534.1	
	Copidosoma_floridanum_(wasps,_ants,_and_bees)_Insecta_Encyrtidae_XP_014210141.1		Tribolium_castaneum_(red_flour_beetle)_Insecta_Tenebrionidae_NP_001164533.1	
	Anopheles_albimanus_(mosquitos)_Insecta_Culicidae_XP_035785831.1		Habropoda_laboriosa_(bees)_Insecta_Apidae_XP_017793482.1	
	Aethina_tumida_(small_hive_beetle)_Insecta_XP_019881850.1		Anopheles_gambiae_str._PEST_(African_malaria_mosquito)_Insecta_Culicidae_XP_317600.4	
	Agrilus_planipennis_(emerald_ash_borer)_Insecta_Buprestidae_XP_018318404.1		Aedes_albopictus_(Asian_tiger_mosquito)_Insecta_Culicidae_XP_019558819.2	
	Diabrotica_virgifera_virgifera_(western_corn_rootworm)_Insecta_Chrysomelidae_XP_028135822.1		Pararge_aegeria_(specked_wood_butterfly)_Insecta_XP_039762966.1	
	Onthophagus_taurus_(beetles)_Insecta_Scarabaeidae_XP_022917833.1		Diabrotica_virgifera_virgifera_(western_corn_rootworm)_Insecta_Chrysomelidae_XP_028134782.1	
	Nicrophorus_vespilloides_(beetles)_Insecta_Silphidae_XP_017769385.1			
	Leptinotarsa_decemlineata_(Colorado_potato_beetle)_Insecta_Chrysomelidae_XP_023018813.1			
	Anoplophora_glabripennis_(Asian_longhorned_beetle)_Insecta_Cerambycidae_XP_018576461.1			
	Chelonus_insularis_(wasps,_ants,_and_bees)_Insecta_Braconidae_XP_034949850.1			

Table S2

Human gene	*D. melanogaster* homologs
PGM1	*Pgm1* (NP_524675.1)
PGM2	*Pgm2a* (NP_610453.1)
	Pgm2b (NP_610992.2)
UGP2	*UGP* (NP_001163399.1)
UGDH	*sugarless* (NP_476980.1)
AKR1B1	*Akr1B* (NP_001261717.1)
	CG6083 (NP_648485.1)
	CG12766 (NP_647839.1)
	CG10863 (NP_647840.1)
	CG10638 (NP_996068.1 and NP_729808.1)
	CG9436 (NP_610235.1)

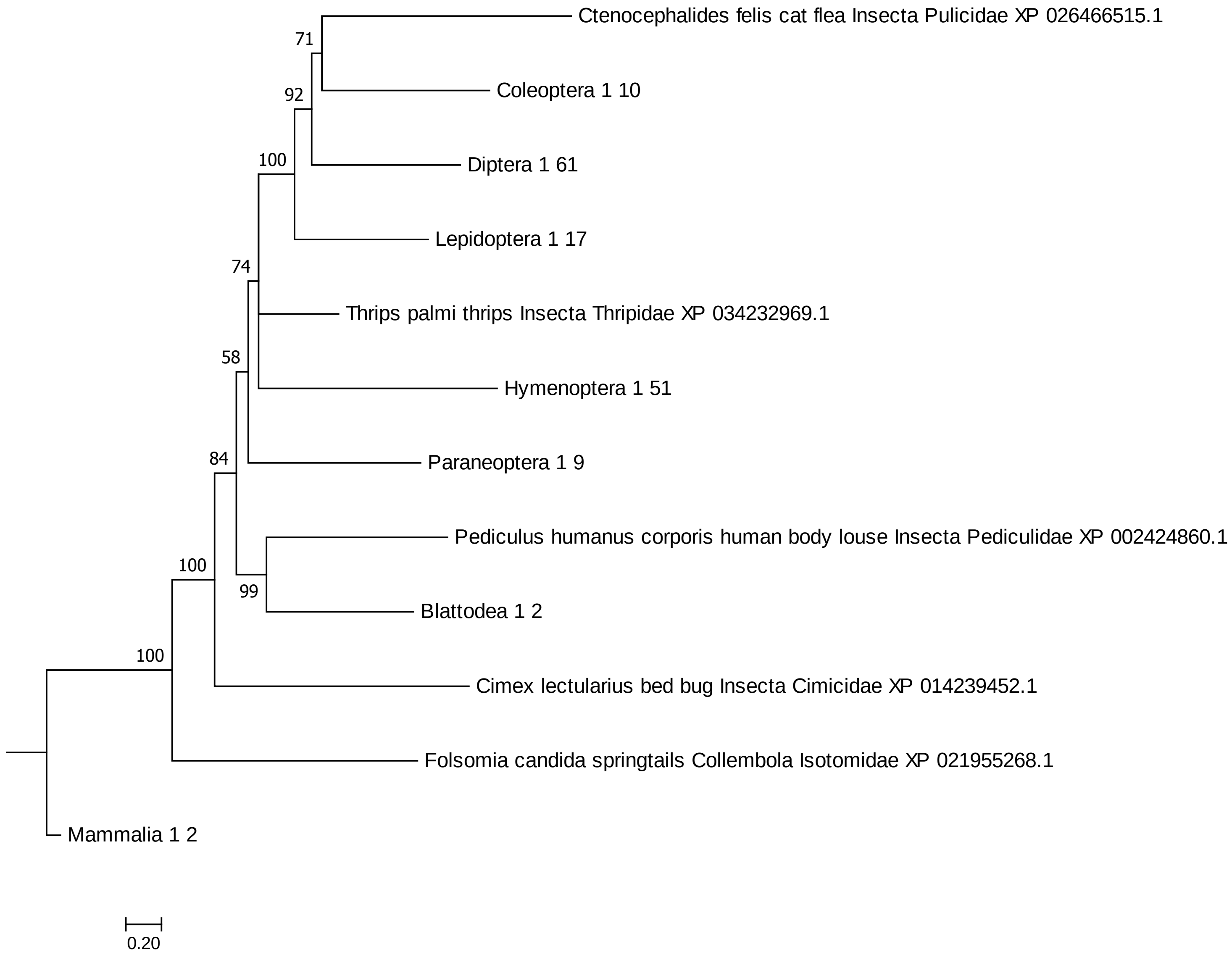
Ctenocephalides felis cat flea Insecta Pulicidae XP 026466515.1
Coleoptera 1 10
Diptera 1 61
Lepidoptera 1 17
Thrips palmi thrips Insecta Thripidae XP 034232969.1
Hymenoptera 1 51
Paraneoptera 1 9
Pediculus humanus corporis human body louse Insecta Pediculidae XP 002424860.1
Blattodea 1 2
Cimex lectularius bed bug Insecta Cimicidae XP 014239452.1
Folsomia candida springtails Collembola Isotomidae XP 021955268.1
Mammalia 1 2
71
92
100
74
58
84
100
99
100
0.20

Figure S1

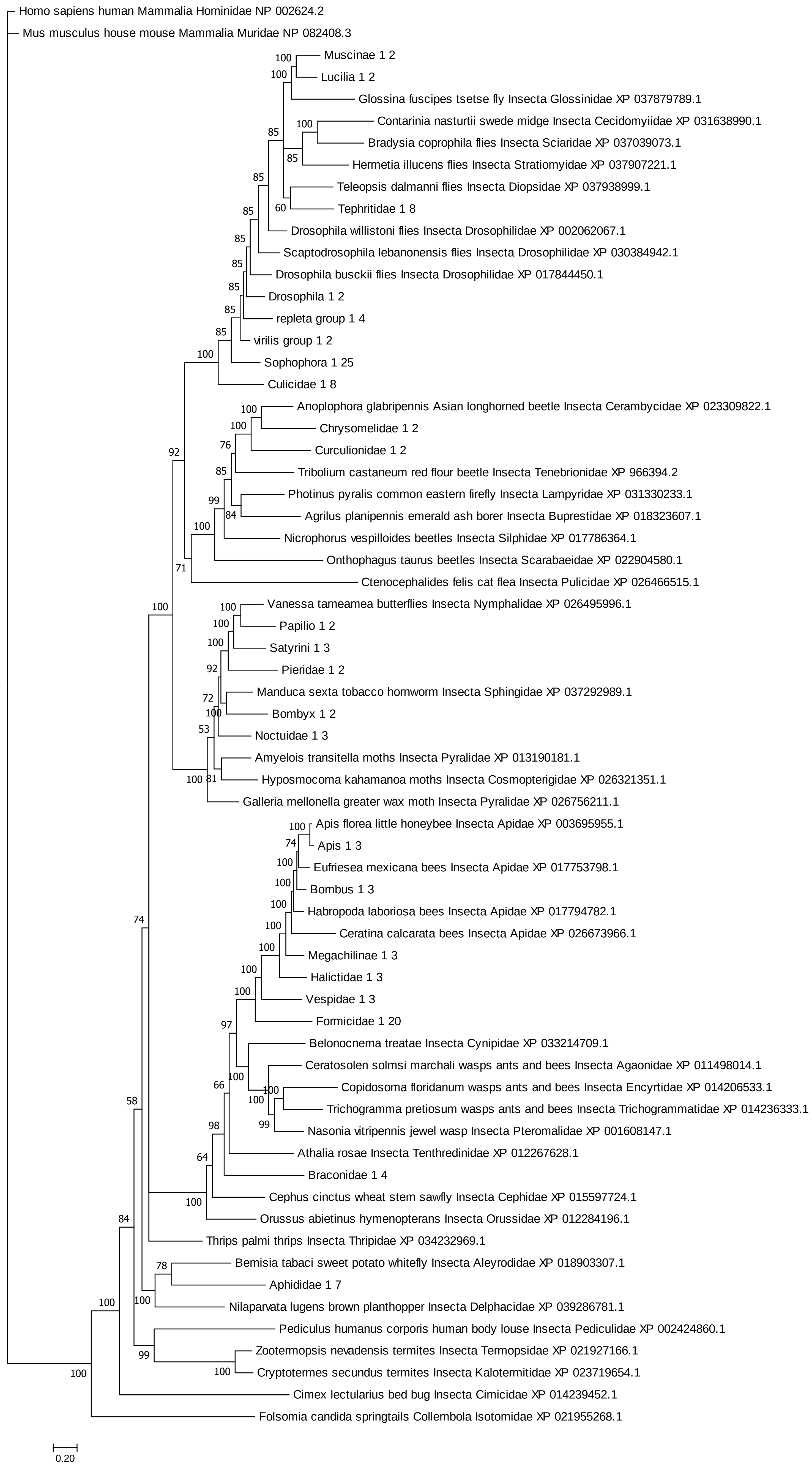
Homo sapiens human Mammalia Hominidae NP 002624.2
Mus musculus house mouse Mammalia Muridae NP 082408.3
Muscinae 1 2
Lucilia 1 2
Glossina fuscipes tsetse fly Insecta Glossinidae XP 037879789.1
Contarinia nasturtii swede midge Insecta Cecidomyiidae XP 031638990.1
Bradysia coprophila flies Insecta Sciaridae XP 037039073.1
Hermetia illucens flies Insecta Stratiomyidae XP 037907221.1
Teleopsis dalmanni flies Insecta Diopsidae XP 037938999.1
Tephritidae 1 8
Drosophila willistoni flies Insecta Drosophilidae XP 002062067.1
Scaptodrosophila lebanonensis flies Insecta Drosophilidae XP 030384942.1
Drosophila busckii flies Insecta Drosophilidae XP 017844450.1
Drosophila 1 2
repleta group 1 4
virilis group 1 2
Sophophora 1 25
Culicidae 1 8
Anoplophora glabripennis Asian longhorned beetle Insecta Cerambycidae XP 023309822.1
Chrysomelidae 1 2
Curculionidae 1 2
Tribolium castaneum red flour beetle Insecta Tenebrionidae XP 966394.2
Photinus pyralis common eastern firefly Insecta Lampyridae XP 031330233.1
Agrilus planipennis emerald ash borer Insecta Buprestidae XP 018323607.1
Nicrophorus vespilloides beetles Insecta Silphidae XP 017786364.1
Onthophagus taurus beetles Insecta Scarabaeidae XP 022904580.1
Ctenocephalides felis cat flea Insecta Pulicidae XP 026466515.1
Vanessa tameamea butterflies Insecta Nymphalidae XP 026495996.1
Papilio 1 2
Satyrini 1 3
Pieridae 1 2
Manduca sexta tobacco hornworm Insecta Sphingidae XP 037292989.1
Bombyx 1 2
Noctuidae 1 3
Amyelois transitella moths Insecta Pyralidae XP 013190181.1
Hyposmocoma kahamanoa moths Insecta Cosmopterigidae XP 026321351.1
Galleria mellonella greater wax moth Insecta Pyralidae XP 026756211.1
Apis florea little honeybee Insecta Apidae XP 003695955.1
Apis 1 3
Eufriesea mexicana bees Insecta Apidae XP 017753798.1
Bombus 1 3
Habropoda laboriosa bees Insecta Apidae XP 017794782.1
Ceratina calcarata bees Insecta Apidae XP 026673966.1
Megachilinae 1 3
Halictidae 1 3
Vespidae 1 3
Formicidae 1 20
Belonocnema treatae Insecta Cynipidae XP 033214709.1
Ceratosolen solmsi marchali wasps ants and bees Insecta Agaonidae XP 011498014.1
Copidosoma floridanum wasps ants and bees Insecta Encyrtidae XP 014206533.1
Trichogramma pretiosum wasps ants and bees Insecta Trichogrammatidae XP 014236333.1
Nasonia vitripennis jewel wasp Insecta Pteromalidae XP 001608147.1
Athalia rosae Insecta Tenthredinidae XP 012267628.1
Braconidae 1 4
Cephus cinctus wheat stem sawfly Insecta Cephidae XP 015597724.1
Orussus abietinus hymenopterans Insecta Orussidae XP 012284196.1
Thrips palmi thrips Insecta Thripidae XP 034232969.1
Bemisia tabaci sweet potato whitefly Insecta Aleyrodidae XP 018903307.1
Aphididae 1 7
Nilaparvata lugens brown planthopper Insecta Delphacidae XP 039286781.1
Pediculus humanus corporis human body louse Insecta Pediculidae XP 002424860.1
Zootermopsis nevadensis termites Insecta Termopsidae XP 021927166.1
Cryptotermes secundus termites Insecta Kalotermitidae XP 023719654.1
Cimex lectularius bed bug Insecta Cimicidae XP 014239452.1
Folsomia candida springtails Collembola Isotomidae XP 021955268.1
0.20

Figure S2

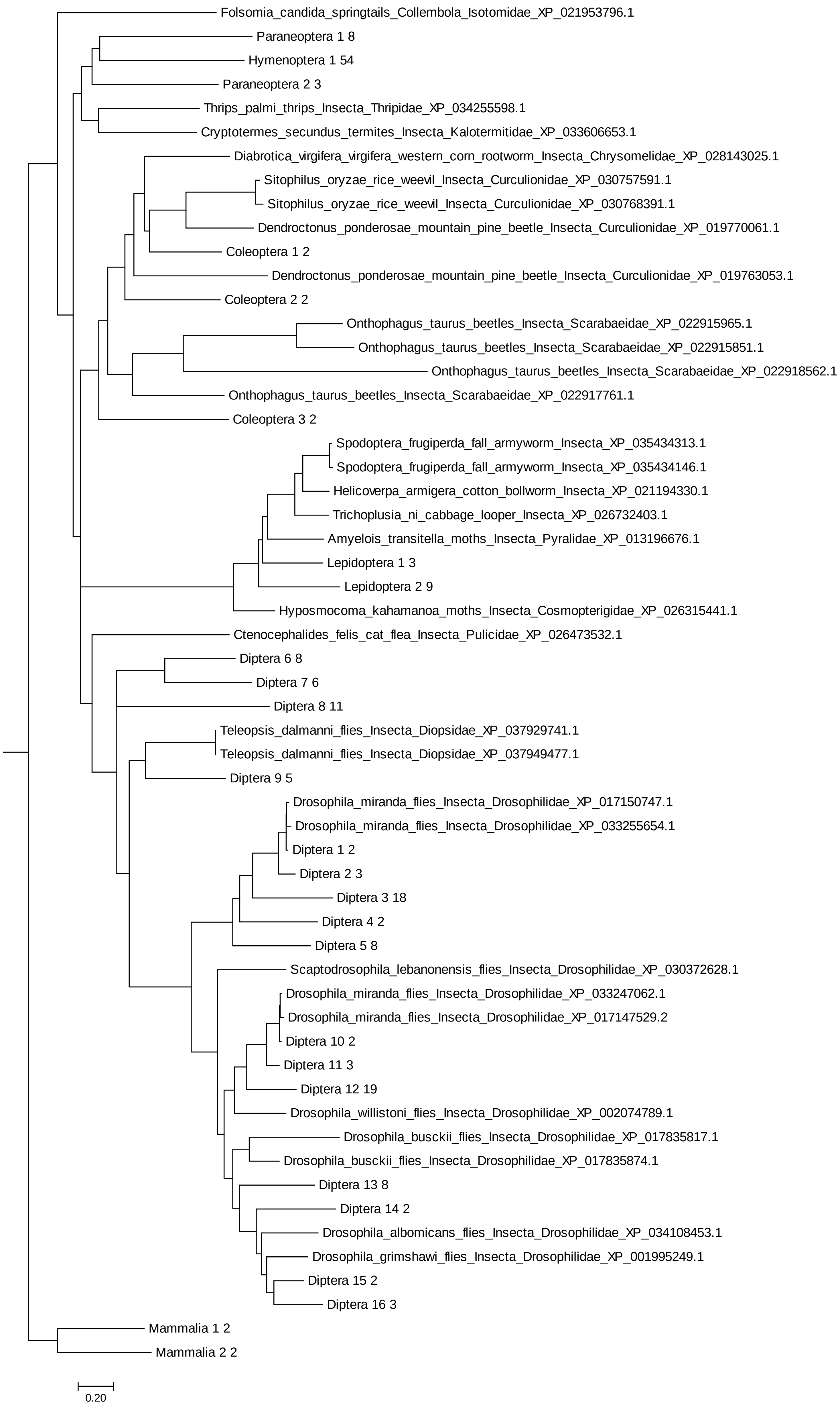
Folsomia_candida_springtails_Collembola_Isotomidae_XP_021953796.1
Paraneoptera 1 8
Hymenoptera 1 54
Paraneoptera 2 3
Thrips_palmi_thrips_Insecta_Thripidae_XP_034255598.1
Cryptotermes_secundus_termites_Insecta_Kalotermitidae_XP_033606653.1
Diabrotica_virgifera_virgifera_western_corn_rootworm_Insecta_Chrysomelidae_XP_028143025.1
Sitophilus_oryzae_rice_weevil_Insecta_Curculionidae_XP_030757591.1
Sitophilus_oryzae_rice_weevil_Insecta_Curculionidae_XP_030768391.1
Dendroctonus_ponderosae_mountain_pine_beetle_Insecta_Curculionidae_XP_019770061.1
Coleoptera 1 2
Dendroctonus_ponderosae_mountain_pine_beetle_Insecta_Curculionidae_XP_019763053.1
Coleoptera 2 2
Onthophagus_taurus_beetles_Insecta_Scarabaeidae_XP_022915965.1
Onthophagus_taurus_beetles_Insecta_Scarabaeidae_XP_022915851.1
Onthophagus_taurus_beetles_Insecta_Scarabaeidae_XP_022918562.1
Onthophagus_taurus_beetles_Insecta_Scarabaeidae_XP_022917761.1
Coleoptera 3 2
Spodoptera_frugiperda_fall_armyworm_Insecta_Noctuidae_XP_035434313.1
Spodoptera_frugiperda_fall_armyworm_Insecta_Noctuidae_XP_035434146.1
Helicoverpa_armigera_cotton_bollworm_Insecta_Noctuidae_XP_021194330.1
Trichoplusia_ni_cabbage_looper_Insecta_Noctuidae_XP_026732403.1
Amyelois_transitella_moths_Insecta_Pyralidae_XP_013196676.1
Lepidoptera 1 3
Lepidoptera 2 9
Hyposmocoma_kahamanoa_moths_Insecta_Cosmopterigidae_XP_026315441.1
Ctenocephalides_felis_cat_flea_Insecta_Pulicidae_XP_026473532.1
Diptera 6 8
Diptera 7 6
Diptera 8 11
Teleopsis_dalmanni_flies_Insecta_Diopsidae_XP_037929741.1
Teleopsis_dalmanni_flies_Insecta_Diopsidae_XP_037949477.1
Diptera 9 5
Drosophila_miranda_flies_Insecta_Drosophilidae_XP_017150747.1
Drosophila_miranda_flies_Insecta_Drosophilidae_XP_033255654.1
Diptera 1 2
Diptera 2 3
Diptera 3 18
Diptera 4 2
Diptera 5 8
Scaptodrosophila_lebanonensis_flies_Insecta_Drosophilidae_XP_030372628.1
Drosophila_miranda_flies_Insecta_Drosophilidae_XP_033247062.1
Drosophila_miranda_flies_Insecta_Drosophilidae_XP_017147529.2
Diptera 10 2
Diptera 11 3
Diptera 12 19
Drosophila_willistoni_flies_Insecta_Drosophilidae_XP_002074789.1
Drosophila_busckii_flies_Insecta_Drosophilidae_XP_017835817.1
Drosophila_busckii_flies_Insecta_Drosophilidae_XP_017835874.1
Diptera 13 8
Diptera 14 2
Drosophila_albomicans_flies_Insecta_Drosophilidae_XP_034108453.1
Drosophila_grimshawi_flies_Insecta_Drosophilidae_XP_001995249.1
Diptera 15 2
Diptera 16 3
Mammalia 1 2
Mammalia 2 2
0.20

Figure S3

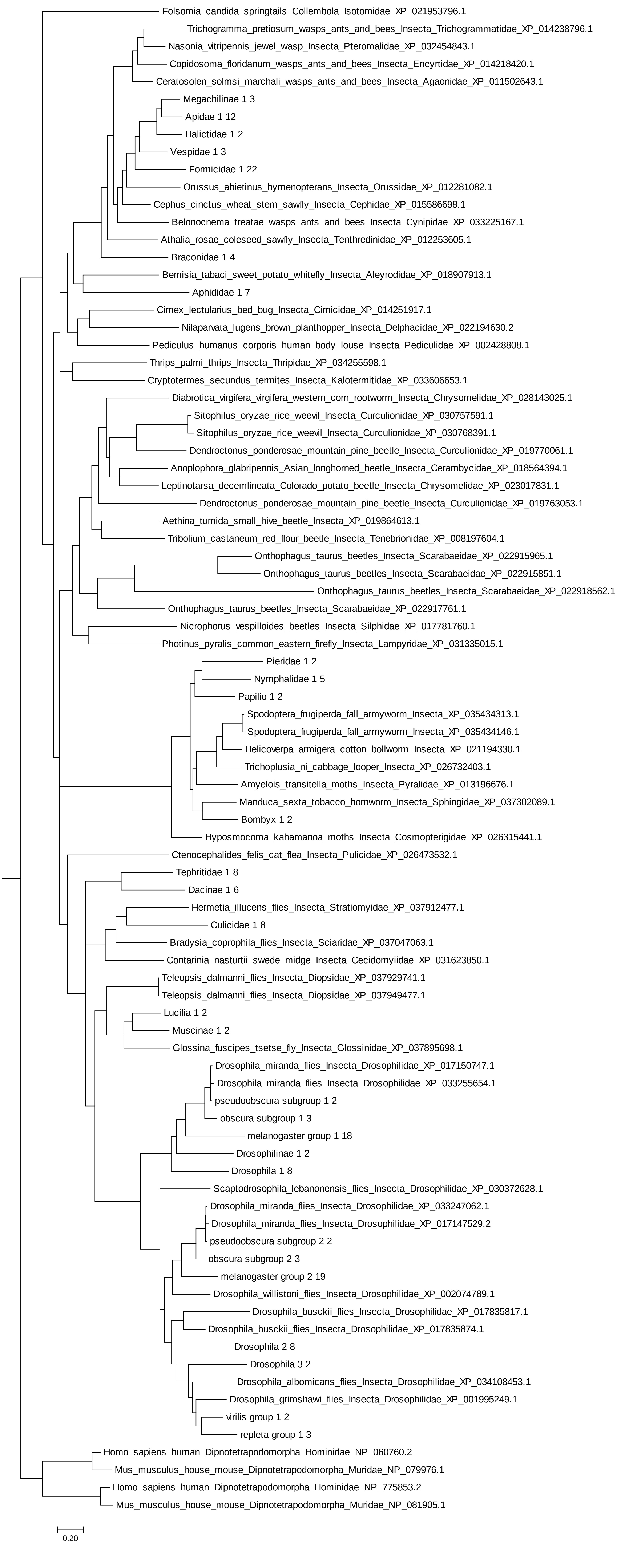
Folsomia_candida_springtails_Collembola_Isotomidae_XP_021953796.1
Trichogramma_pretiosum_wasps_ants_and_bees_Insecta_Trichogrammatidae_XP_014238796.1
Nasonia_vitripennis_jewel_wasp_Insecta_Pteromalidae_XP_032454843.1
Copidosoma_floridanum_wasps_ants_and_bees_Insecta_Encyrtidae_XP_014218420.1
Ceratosolen_solmsi_marchali_wasps_ants_and_bees_Insecta_Agaonidae_XP_011502643.1
Megachilinae 1 3
Apidae 1 12
Halictidae 1 2
Vespidae 1 3
Formicidae 1 22
Orussus_abietinus_hymenopterans_Insecta_Orussidae_XP_012281082.1
Cephus_cinctus_wheat_stem_sawfly_Insecta_Cephidae_XP_015586698.1
Belonocnema_treatae_wasps_ants_and_bees_Insecta_Cynipidae_XP_033225167.1
Athalia_rosae_coleseed_sawfly_Insecta_Tenthredinidae_XP_012253605.1
Braconidae 1 4
Bemisia_tabaci_sweet_potato_whitefly_Insecta_Aleyrodidae_XP_018907913.1
Aphididae 1 7
Cimex_lectularius_bed_bug_Insecta_Cimicidae_XP_014251917.1
Nilaparvata_lugens_brown_planthopper_Insecta_Delphacidae_XP_022194630.2
Pediculus_humanus_corporis_human_body_louse_Insecta_Pediculidae_XP_002428808.1
Thrips_palmi_thrips_Insecta_Thripidae_XP_034255598.1
Cryptotermes_secundus_termites_Insecta_Kalotermitidae_XP_033606653.1
Diabrotica_virgifera_virgifera_western_corn_rootworm_Insecta_Chrysomelidae_XP_028143025.1
Sitophilus_oryzae_rice_weevil_Insecta_Curculionidae_XP_030757591.1
Sitophilus_oryzae_rice_weevil_Insecta_Curculionidae_XP_030768391.1
Dendroctonus_ponderosae_mountain_pine_beetle_Insecta_Curculionidae_XP_019770061.1
Anoplophora_glabripennis_Asian_longhorned_beetle_Insecta_Cerambycidae_XP_018564394.1
Leptinotarsa_decemlineata_Colorado_potato_beetle_Insecta_Chrysomelidae_XP_023017831.1
Dendroctonus_ponderosae_mountain_pine_beetle_Insecta_Curculionidae_XP_019763053.1
Aethina_tumida_small_hive_beetle_Insecta_XP_019864613.1
Tribolium_castaneum_red_flour_beetle_Insecta_Tenebrionidae_XP_008197604.1
Onthophagus_taurus_beetles_Insecta_Scarabaeidae_XP_022915965.1
Onthophagus_taurus_beetles_Insecta_Scarabaeidae_XP_022915851.1
Onthophagus_taurus_beetles_Insecta_Scarabaeidae_XP_022918562.1
Onthophagus_taurus_beetles_Insecta_Scarabaeidae_XP_022917761.1
Nicrophorus_vespilloides_beetles_Insecta_Silphidae_XP_017781760.1
Photinus_pyralis_common_eastern_firefly_Insecta_Lampyridae_XP_031335015.1
Pieridae 1 2
Nymphalidae 1 5
Papilio 1 2
Spodoptera_frugiperda_fall_armyworm_Insecta_XP_035434313.1
Spodoptera_frugiperda_fall_armyworm_Insecta_XP_035434146.1
Helicoverpa_armigera_cotton_bollworm_Insecta_XP_021194330.1
Trichoplusia_ni_cabbage_looper_Insecta_XP_026732403.1
Amyelois_transitella_moths_Insecta_Pyralidae_XP_013196676.1
Manduca_sexta_tobacco_hornworm_Insecta_Sphingidae_XP_037302089.1
Bombyx 1 2
Hyposmocoma_kahamanoa_moths_Insecta_Cosmopterigidae_XP_026315441.1
Ctenocephalides_felis_cat_flea_Insecta_Pulicidae_XP_026473532.1
Tephritidae 1 8
Dacinae 1 6
Hermetia_illucens_flies_Insecta_Stratiomyidae_XP_037912477.1
Culicidae 1 8
Bradysia_coprophila_flies_Insecta_Sciaridae_XP_037047063.1
Contarinia_nasturtii_swede_midge_Insecta_Cecidomyiidae_XP_031623850.1
Teleopsis_dalmanni_flies_Insecta_Diopsidae_XP_037929741.1
Teleopsis_dalmanni_flies_Insecta_Diopsidae_XP_037949477.1
Lucilia 1 2
Muscinae 1 2
Glossina_fuscipes_tsetse_fly_Insecta_Glossinidae_XP_037895698.1
Drosophila_miranda_flies_Insecta_Drosophilidae_XP_017150747.1
Drosophila_miranda_flies_Insecta_Drosophilidae_XP_033255654.1
pseudoobscura subgroup 1 2
obscura subgroup 1 3
melanogaster group 1 18
Drosophilinae 1 2
Drosophila 1 8
Scaptodrosophila_lebanonensis_flies_Insecta_Drosophilidae_XP_030372628.1
Drosophila_miranda_flies_Insecta_Drosophilidae_XP_033247062.1
Drosophila_miranda_flies_Insecta_Drosophilidae_XP_017147529.2
pseudoobscura subgroup 2 2
obscura subgroup 2 3
melanogaster group 2 19
Drosophila_willistoni_flies_Insecta_Drosophilidae_XP_002074789.1
Drosophila_busckii_flies_Insecta_Drosophilidae_XP_017835817.1
Drosophila_busckii_flies_Insecta_Drosophilidae_XP_017835874.1
Drosophila 2 8
Drosophila 3 2
Drosophila_albomicans_flies_Insecta_Drosophilidae_XP_034108453.1
Drosophila_grimshawi_flies_Insecta_Drosophilidae_XP_001995249.1
virilis group 1 2
repleta group 1 3
Homo_sapiens_human_Dipnotetrapodomorpha_Hominidae_NP_060760.2
Mus_musculus_house_mouse_Dipnotetrapodomorpha_Muridae_NP_079976.1
Homo_sapiens_human_Dipnotetrapodomorpha_Hominidae_NP_775853.2
Mus_musculus_house_mouse_Dipnotetrapodomorpha_Muridae_NP_081905.1
0.20

Figure S4

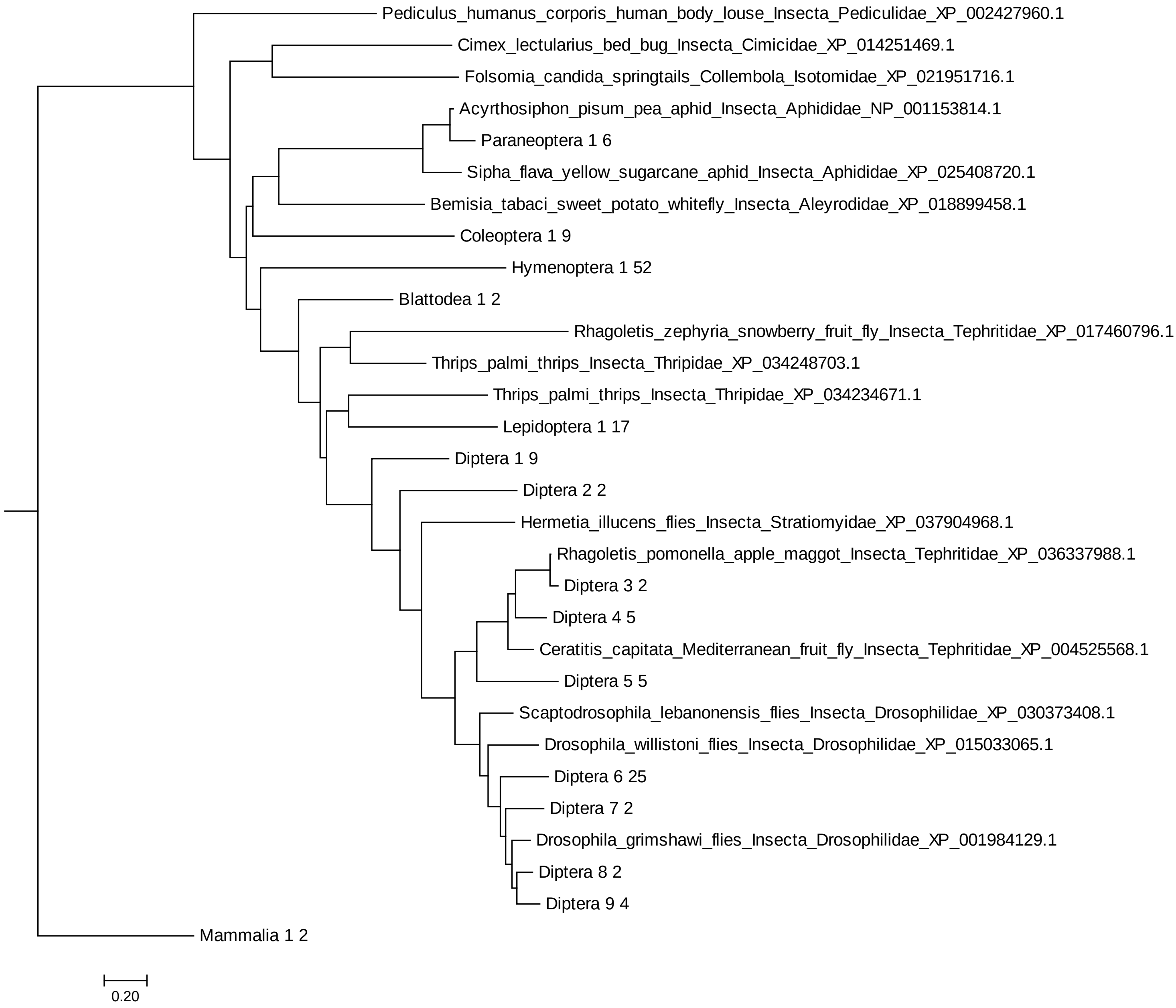
Pediculus_humanus_corporis_human_body_louse_Insecta_Pediculidae_XP_002427960.1
Cimex_lectularius_bed_bug_Insecta_Cimicidae_XP_014251469.1
Folsomia_candida_springtails_Collembola_Isotomidae_XP_021951716.1
Acyrthosiphon_pisum_pea_aphid_Insecta_Aphididae_NP_001153814.1
Paraneoptera 1 6
Sipha_flava_yellow_sugarcane_aphid_Insecta_Aphididae_XP_025408720.1
Bemisia_tabaci_sweet_potato_whitefly_Insecta_Aleyrodidae_XP_018899458.1
Coleoptera 1 9
Hymenoptera 1 52
Blattodea 1 2
Rhagoletis_zephyria_snowberry_fruit_fly_Insecta_Tephritidae_XP_017460796.1
Thrips_palmi_thrips_Insecta_Thripidae_XP_034248703.1
Thrips_palmi_thrips_Insecta_Thripidae_XP_034234671.1
Lepidoptera 1 17
Diptera 1 9
Diptera 2 2
Hermetia_illucens_flies_Insecta_Stratiomyidae_XP_037904968.1
Rhagoletis_pomonella_apple_maggot_Insecta_Tephritidae_XP_036337988.1
Diptera 3 2
Diptera 4 5
Ceratitis_capitata_Mediterranean_fruit_fly_Insecta_Tephritidae_XP_004525568.1
Diptera 5 5
Scaptodrosophila_lebanonensis_flies_Insecta_Drosophilidae_XP_030373408.1
Drosophila_willistoni_flies_Insecta_Drosophilidae_XP_015033065.1
Diptera 6 25
Diptera 7 2
Drosophila_grimshawi_flies_Insecta_Drosophilidae_XP_001984129.1
Diptera 8 2
Diptera 9 4
Mammalia 1 2
0.20

Figure S5

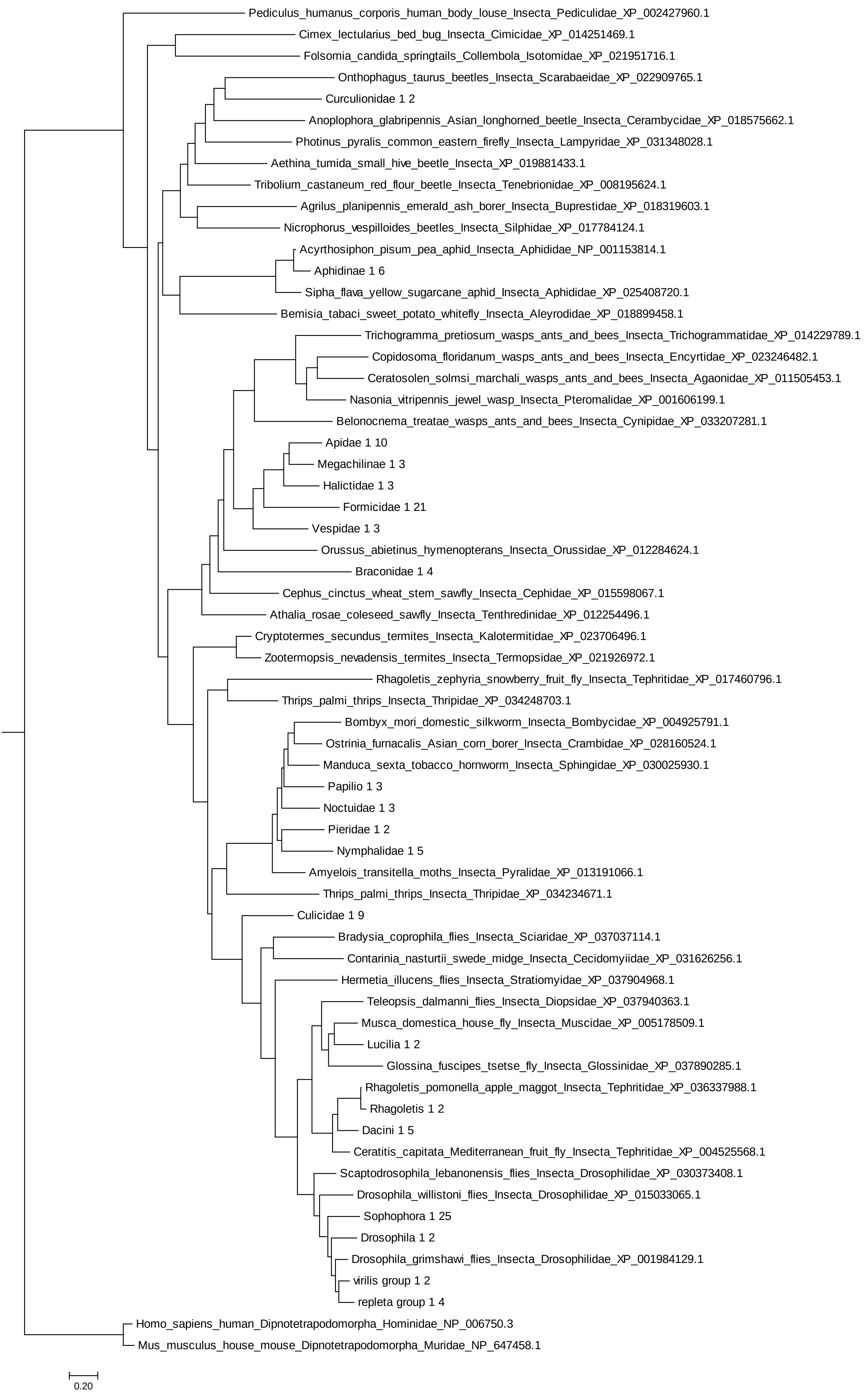
Pediculus_humanus_corporis_human_body_louse_Insecta_Pediculidae_XP_002427960.1
Cimex_lectularius_bed_bug_Insecta_Cimicidae_XP_014251469.1
Folsomia_candida_springtails_Collembola_Isotomidae_XP_021951716.1
Onthophagus_taurus_beetles_Insecta_Scarabaeidae_XP_022909765.1
Curculionidae 1 2
Anoplophora_glabripennis_Asian_longhorned_beetle_Insecta_Cerambycidae_XP_018575662.1
Photinus_pyralis_common_eastern_firefly_Insecta_Lampyridae_XP_031348028.1
Aethina_tumida_small_hive_beetle_Insecta_XP_019881433.1
Tribolium_castaneum_red_flour_beetle_Insecta_Tenebrionidae_XP_008195624.1
Agrilus_planipennis_emerald_ash_borer_Insecta_Buprestidae_XP_018319603.1
Nicrophorus_vespilloides_beetles_Insecta_Silphidae_XP_017784124.1
Acyrthosiphon_pisum_pea_aphid_Insecta_Aphididae_NP_001153814.1
Aphidinae 1 6
Sipha_flava_yellow_sugarcane_aphid_Insecta_Aphididae_XP_025408720.1
Bemisia_tabaci_sweet_potato_whitefly_Insecta_Aleyrodidae_XP_018899458.1
Trichogramma_pretiosum_wasps_ants_and_bees_Insecta_Trichogrammatidae_XP_014229789.1
Copidosoma_floridanum_wasps_ants_and_bees_Insecta_Encyrtidae_XP_023246482.1
Ceratosolen_solmsi_marchali_wasps_ants_and_bees_Insecta_Agaonidae_XP_011505453.1
Nasonia_vitripennis_jewel_wasp_Insecta_Pteromalidae_XP_001606199.1
Belonocnema_treatae_wasps_ants_and_bees_Insecta_Cynipidae_XP_033207281.1
Apidae 1 10
Megachilinae 1 3
Halictidae 1 3
Formicidae 1 21
Vespidae 1 3
Orussus_abietinus_hymenopterans_Insecta_Orussidae_XP_012284624.1
Braconidae 1 4
Cephus_cinctus_wheat_stem_sawfly_Insecta_Cephidae_XP_015598067.1
Athalia_rosae_coleseed_sawfly_Insecta_Tenthredinidae_XP_012254496.1
Cryptotermes_secundus_termites_Insecta_Kalotermitidae_XP_023706496.1
Zootermopsis_nevadensis_termites_Insecta_Termopsidae_XP_021926972.1
Rhagoletis_zephyria_snowberry_fruit_fly_Insecta_Tephritidae_XP_017460796.1
Thrips_palmi_thrips_Insecta_Thripidae_XP_034248703.1
Bombyx_mori_domestic_silkworm_Insecta_Bombycidae_XP_004925791.1
Ostrinia_furnacalis_Asian_corn_borer_Insecta_Crambidae_XP_028160524.1
Manduca_sexta_tobacco_hornworm_Insecta_Sphingidae_XP_030025930.1
Papilio 1 3
Noctuidae 1 3
Pieridae 1 2
Nymphalidae 1 5
Amyelois_transitella_moths_Insecta_Pyralidae_XP_013191066.1
Thrips_palmi_thrips_Insecta_Thripidae_XP_034234671.1
Culicidae 1 9
Bradysia_coprophila_flies_Insecta_Sciaridae_XP_037037114.1
Contarinia_nasturtii_swede_midge_Insecta_Cecidomyiidae_XP_031626256.1
Hermetia_illucens_flies_Insecta_Stratiomyidae_XP_037904968.1
Teleopsis_dalmanni_flies_Insecta_Diopsidae_XP_037940363.1
Musca_domestica_house_fly_Insecta_Muscidae_XP_005178509.1
Lucilia 1 2
Glossina_fuscipes_tsetse_fly_Insecta_Glossinidae_XP_037890285.1
Rhagoletis_pomonella_apple_maggot_Insecta_Tephritidae_XP_036337988.1
Rhagoletis 1 2
Dacini 1 5
Ceratitis_capitata_Mediterranean_fruit_fly_Insecta_Tephritidae_XP_004525568.1
Scaptodrosophila_lebanonensis_flies_Insecta_Drosophilidae_XP_030373408.1
Drosophila_willistoni_flies_Insecta_Drosophilidae_XP_015033065.1
Sophophora 1 25
Drosophila 1 2
Drosophila_grimshawi_flies_Insecta_Drosophilidae_XP_001984129.1
virilis group 1 2
repleta group 1 4
Homo_sapiens_human_Dipnotetrapodomorpha_Hominidae_NP_006750.3
Mus_musculus_house_mouse_Dipnotetrapodomorpha_Muridae_NP_647458.1
0.20

Figure S6

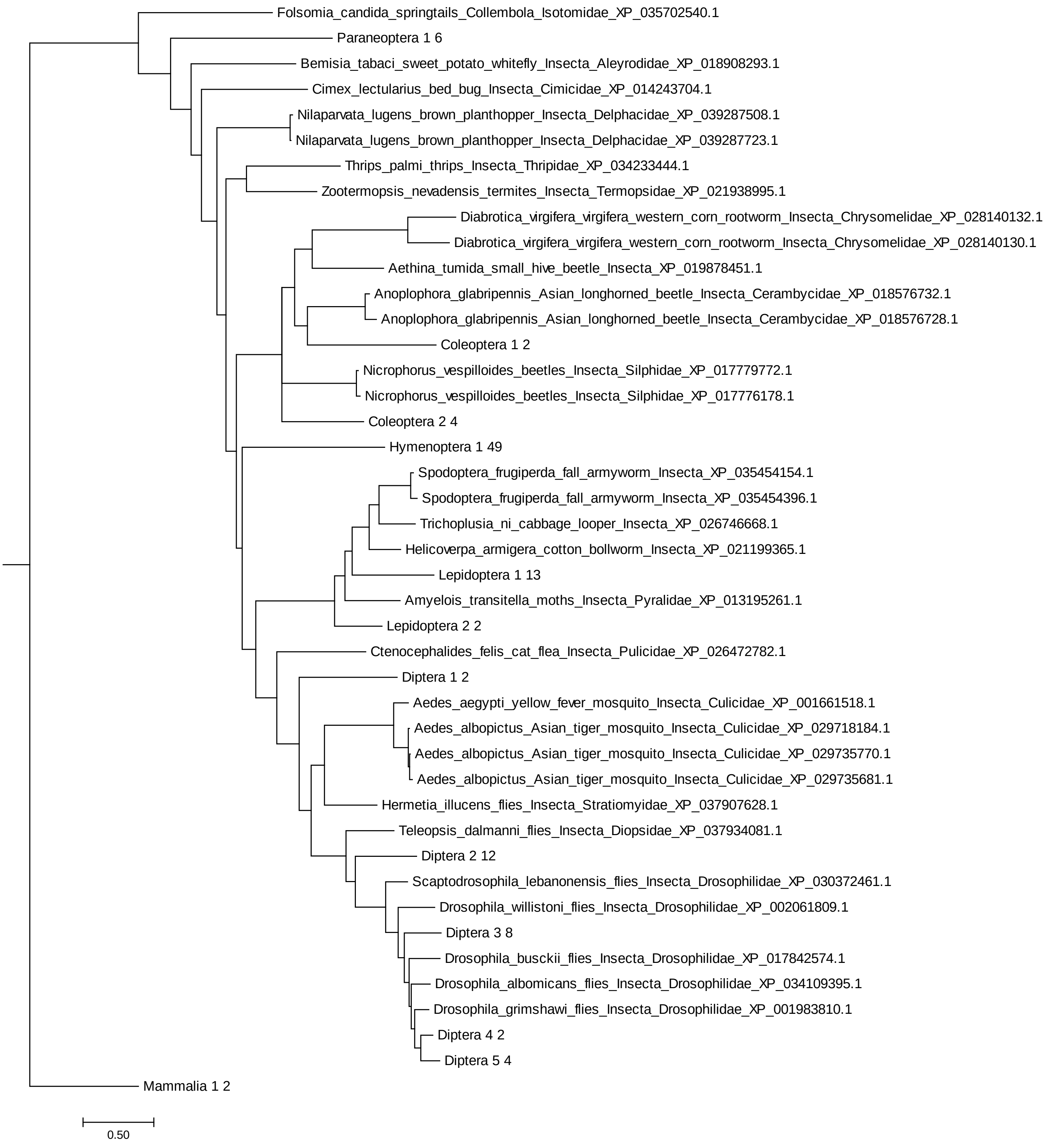
Folsomia_candida_springtails_Collembola_Isotomidae_XP_035702540.1
Paraneoptera 1 6
Bemisia_tabaci_sweet_potato_whitefly_Insecta_Aleyrodidae_XP_018908293.1
Cimex_lectularius_bed_bug_Insecta_Cimicidae_XP_014243704.1
Nilaparvata_lugens_brown_planthopper_Insecta_Delphacidae_XP_039287508.1
Nilaparvata_lugens_brown_planthopper_Insecta_Delphacidae_XP_039287723.1
Thrips_palmi_thrips_Insecta_Thripidae_XP_034233444.1
Zootermopsis_nevadensis_termites_Insecta_Termopsidae_XP_021938995.1
Diabrotica_virgifera_virgifera_western_corn_rootworm_Insecta_Chrysomelidae_XP_028140132.1
Diabrotica_virgifera_virgifera_western_corn_rootworm_Insecta_Chrysomelidae_XP_028140130.1
Aethina_tumida_small_hive_beetle_Insecta_XP_019878451.1
Anoplophora_glabripennis_Asian_longhorned_beetle_Insecta_Cerambycidae_XP_018576732.1
Anoplophora_glabripennis_Asian_longhorned_beetle_Insecta_Cerambycidae_XP_018576728.1
Coleoptera 1 2
Nicrophorus_vespilloides_beetles_Insecta_Silphidae_XP_017779772.1
Nicrophorus_vespilloides_beetles_Insecta_Silphidae_XP_017776178.1
Coleoptera 2 4
Hymenoptera 1 49
Spodoptera_frugiperda_fall_armyworm_Insecta_XP_035454154.1
Spodoptera_frugiperda_fall_armyworm_Insecta_XP_035454396.1
Trichoplusia_ni_cabbage_looper_Insecta_XP_026746668.1
Helicoverpa_armigera_cotton_bollworm_Insecta_XP_021199365.1
Lepidoptera 1 13
Amyelois_transitella_moths_Insecta_Pyralidae_XP_013195261.1
Lepidoptera 2 2
Ctenocephalides_felis_cat_flea_Insecta_Pulicidae_XP_026472782.1
Diptera 1 2
Aedes_aegypti_yellow_fever_mosquito_Insecta_Culicidae_XP_001661518.1
Aedes_albopictus_Asian_tiger_mosquito_Insecta_Culicidae_XP_029718184.1
Aedes_albopictus_Asian_tiger_mosquito_Insecta_Culicidae_XP_029735770.1
Aedes_albopictus_Asian_tiger_mosquito_Insecta_Culicidae_XP_029735681.1
Hermetia_illucens_flies_Insecta_Stratiomyidae_XP_037907628.1
Teleopsis_dalmanni_flies_Insecta_Diopsidae_XP_037934081.1
Diptera 2 12
Scaptodrosophila_lebanonensis_flies_Insecta_Drosophilidae_XP_030372461.1
Drosophila_willistoni_flies_Insecta_Drosophilidae_XP_002061809.1
Diptera 3 8
Drosophila_busckii_flies_Insecta_Drosophilidae_XP_017842574.1
Drosophila_albomicans_flies_Insecta_Drosophilidae_XP_034109395.1
Drosophila_grimshawi_flies_Insecta_Drosophilidae_XP_001983810.1
Diptera 4 2
Diptera 5 4
Mammalia 1 2
0.50

Figure S7

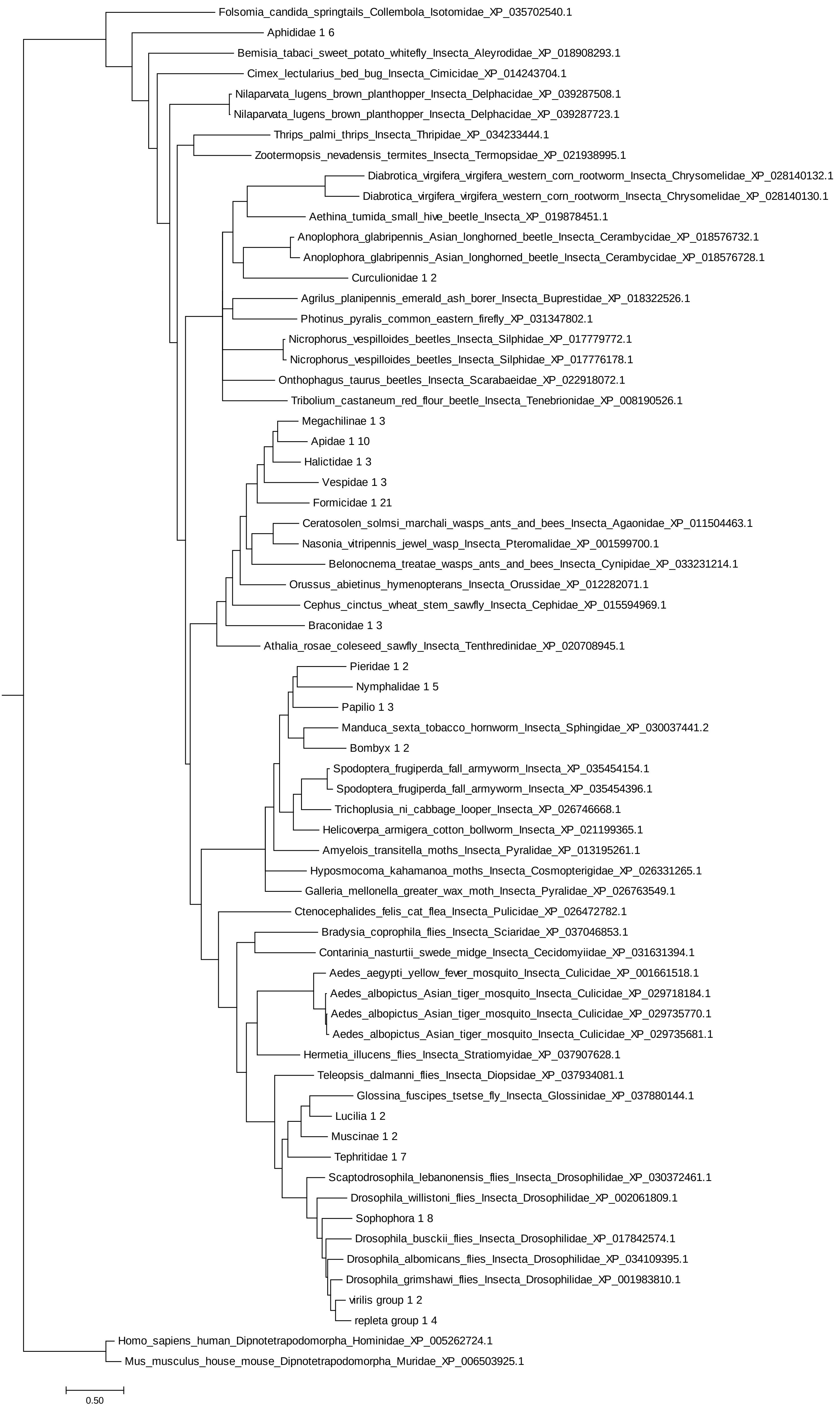
Folsomia_candida_springtails_Collembola_Isotomidae_XP_035702540.1
Aphididae 1 6
Bemisia_tabaci_sweet_potato_whitefly_Insecta_Aleyrodidae_XP_018908293.1
Cimex_lectularius_bed_bug_Insecta_Cimicidae_XP_014243704.1
Nilaparvata_lugens_brown_planthopper_Insecta_Delphacidae_XP_039287508.1
Nilaparvata_lugens_brown_planthopper_Insecta_Delphacidae_XP_039287723.1
Thrips_palmi_thrips_Insecta_Thripidae_XP_034233444.1
Zootermopsis_nevadensis_termites_Insecta_Termopsidae_XP_021938995.1
Diabrotica_virgifera_virgifera_western_corn_rootworm_Insecta_Chrysomelidae_XP_028140132.1
Diabrotica_virgifera_virgifera_western_corn_rootworm_Insecta_Chrysomelidae_XP_028140130.1
Aethina_tumida_small_hive_beetle_Insecta_XP_019878451.1
Anoplophora_glabripennis_Asian_longhorned_beetle_Insecta_Cerambycidae_XP_018576732.1
Anoplophora_glabripennis_Asian_longhorned_beetle_Insecta_Cerambycidae_XP_018576728.1
Curculionidae 1 2
Agrilus_planipennis_emerald_ash_borer_Insecta_Buprestidae_XP_018322526.1
Photinus_pyralis_common_eastern_firefly_XP_031347802.1
Nicrophorus_vespilloides_beetles_Insecta_Silphidae_XP_017779772.1
Nicrophorus_vespilloides_beetles_Insecta_Silphidae_XP_017776178.1
Onthophagus_taurus_beetles_Insecta_Scarabaeidae_XP_022918072.1
Tribolium_castaneum_red_flour_beetle_Insecta_Tenebrionidae_XP_008190526.1
Megachilinae 1 3
Apidae 1 10
Halictidae 1 3
Vespidae 1 3
Formicidae 1 21
Ceratosolen_solmsi_marchali_wasps_ants_and_bees_Insecta_Agaonidae_XP_011504463.1
Nasonia_vitripennis_jewel_wasp_Insecta_Pteromalidae_XP_001599700.1
Belonocnema_treatae_wasps_ants_and_bees_Insecta_Cynipidae_XP_033231214.1
Orussus_abietinus_hymenopterans_Insecta_Orussidae_XP_012282071.1
Cephus_cinctus_wheat_stem_sawfly_Insecta_Cephidae_XP_015594969.1
Braconidae 1 3
Athalia_rosae_coleseed_sawfly_Insecta_Tenthredinidae_XP_020708945.1
Pieridae 1 2
Nymphalidae 1 5
Papilio 1 3
Manduca_sexta_tobacco_hornworm_Insecta_Sphingidae_XP_030037441.2
Bombyx 1 2
Spodoptera_frugiperda_fall_armyworm_Insecta_XP_035454154.1
Spodoptera_frugiperda_fall_armyworm_Insecta_XP_035454396.1
Trichoplusia_ni_cabbage_looper_Insecta_XP_026746668.1
Helicoverpa_armigera_cotton_bollworm_Insecta_XP_021199365.1
Amyelois_transitella_moths_Insecta_Pyralidae_XP_013195261.1
Hyposmocoma_kahamanoa_moths_Insecta_Cosmopterigidae_XP_026331265.1
Galleria_mellonella_greater_wax_moth_Insecta_Pyralidae_XP_026763549.1
Ctenocephalides_felis_cat_flea_Insecta_Pulicidae_XP_026472782.1
Bradysia_coprophila_flies_Insecta_Sciaridae_XP_037046853.1
Contarinia_nasturtii_swede_midge_Insecta_Cecidomyiidae_XP_031631394.1
Aedes_aegypti_yellow_fever_mosquito_Insecta_Culicidae_XP_001661518.1
Aedes_albopictus_Asian_tiger_mosquito_Insecta_Culicidae_XP_029718184.1
Aedes_albopictus_Asian_tiger_mosquito_Insecta_Culicidae_XP_029735770.1
Aedes_albopictus_Asian_tiger_mosquito_Insecta_Culicidae_XP_029735681.1
Hermetia_illucens_flies_Insecta_Stratiomyidae_XP_037907628.1
Teleopsis_dalmanni_flies_Insecta_Diopsidae_XP_037934081.1
Glossina_fuscipes_tsetse_fly_Insecta_Glossinidae_XP_037880144.1
Lucilia 1 2
Muscinae 1 2
Tephritidae 1 7
Scaptodrosophila_lebanonensis_flies_Insecta_Drosophilidae_XP_030372461.1
Drosophila_willistoni_flies_Insecta_Drosophilidae_XP_002061809.1
Sophophora 1 8
Drosophila_busckii_flies_Insecta_Drosophilidae_XP_017842574.1
Drosophila_albomicans_flies_Insecta_Drosophilidae_XP_034109395.1
Drosophila_grimshawi_flies_Insecta_Drosophilidae_XP_001983810.1
virilis group 1 2
repleta group 1 4
Homo_sapiens_human_Dipnotetrapodomorpha_Hominidae_XP_005262724.1
Mus_musculus_house_mouse_Dipnotetrapodomorpha_Muridae_XP_006503925.1
0.50

Figure S8

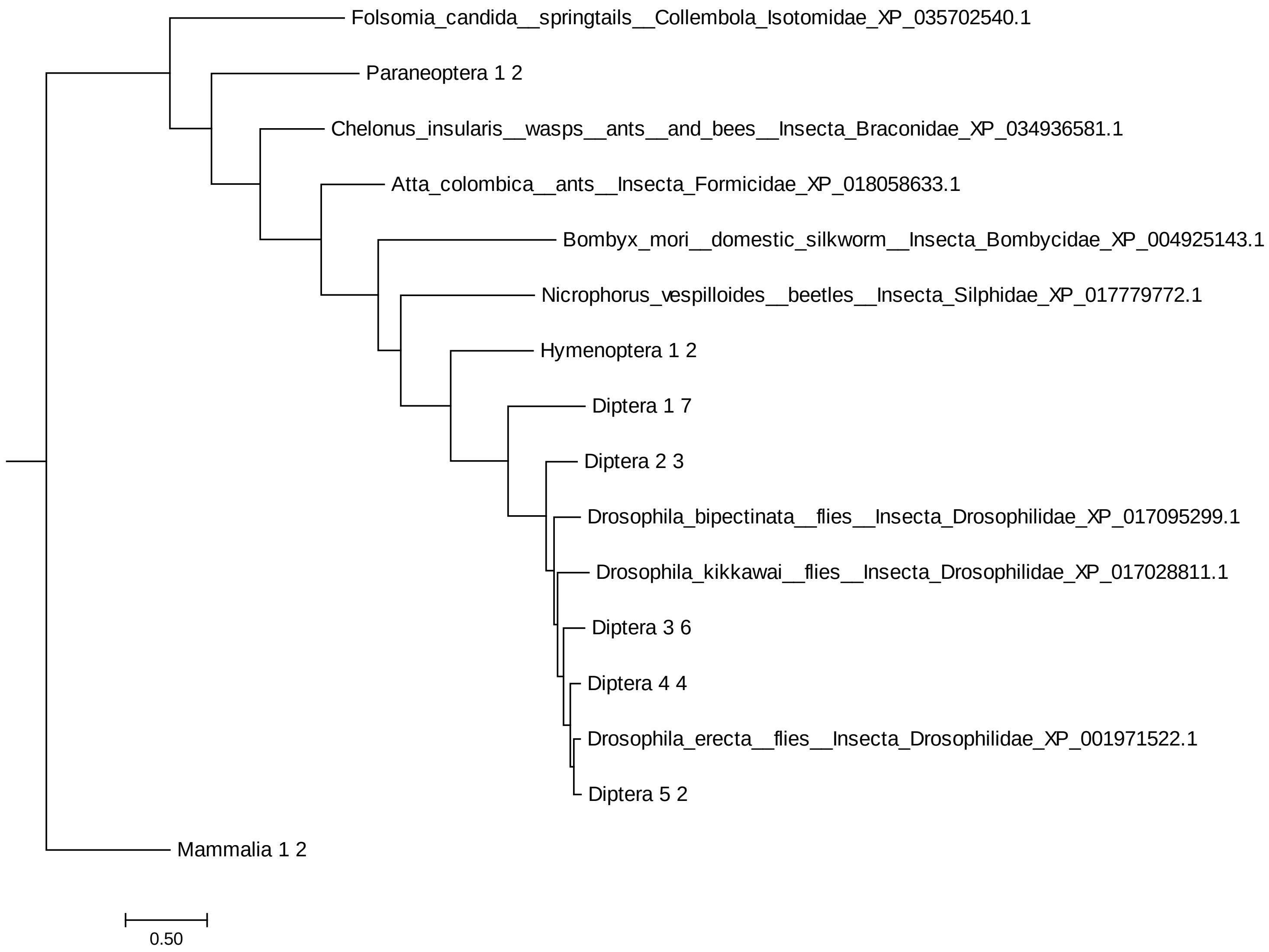
Folsomia_candida__springtails__Collembola_Isotomidae_XP_035702540.1
Paraneoptera 1 2
Chelonus_insularis__wasps__ants__and_bees__Insecta_Braconidae_XP_034936581.1
Atta_colombica__ants__Insecta_Formicidae_XP_018058633.1
Bombyx_mori__domestic_silkworm__Insecta_Bombycidae_XP_004925143.1
Nicrophorus_vespilloides__beetles__Insecta_Silphidae_XP_017779772.1
Hymenoptera 1 2
Diptera 1 7
Diptera 2 3
Drosophila_bipectinata__flies__Insecta_Drosophilidae_XP_017095299.1
Drosophila_kikkawai__flies__Insecta_Drosophilidae_XP_017028811.1
Diptera 3 6
Diptera 4 4
Drosophila_erecta__flies__Insecta_Drosophilidae_XP_001971522.1
Diptera 5 2
Mammalia 1 2
0.50

Figure S9

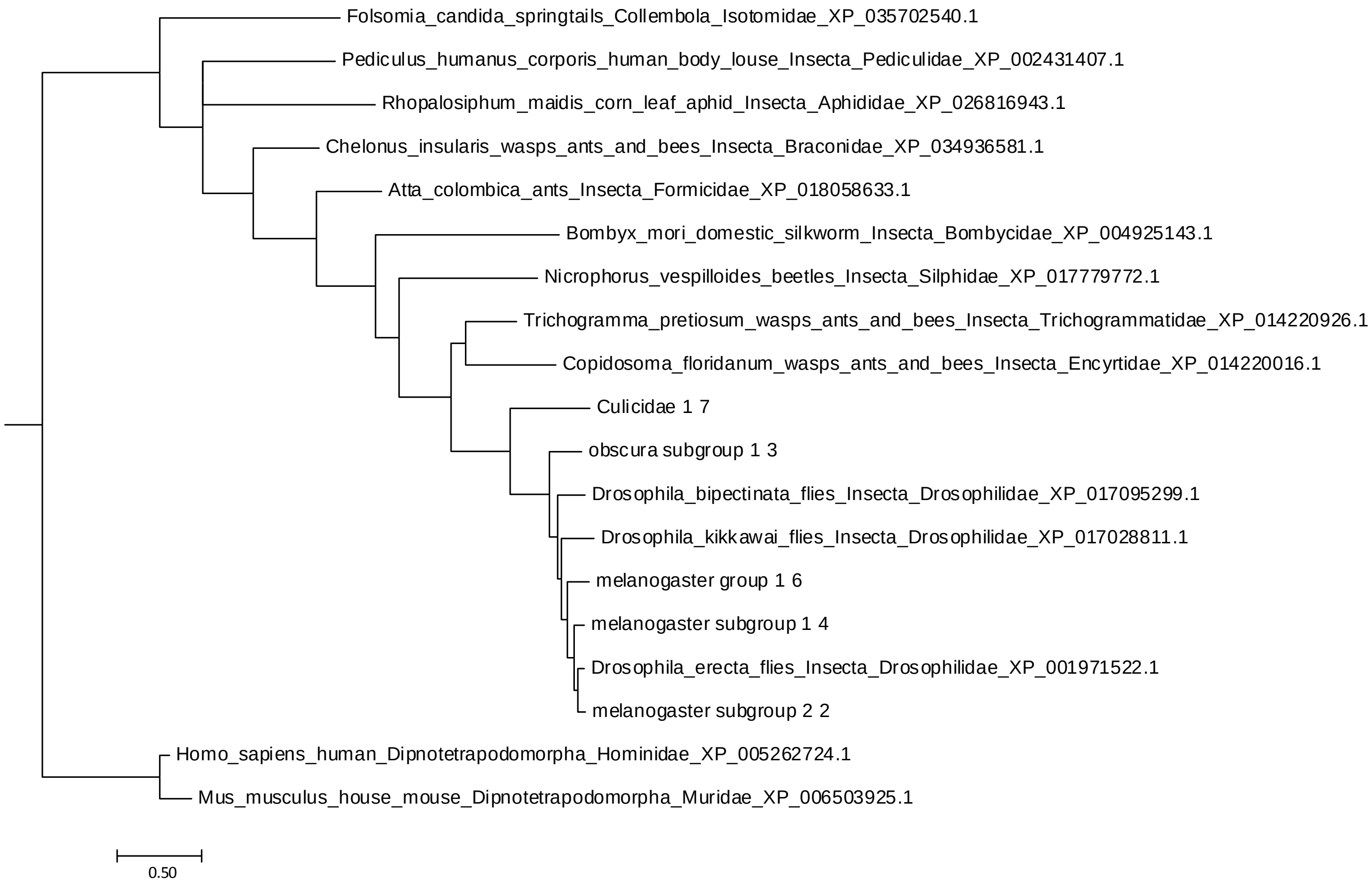
Folsomia_candida_springtails_Collembola_Isotomidae_XP_035702540.1
Pediculus_humanus_corporis_human_body_louse_Insecta_Pediculidae_XP_002431407.1
Rhopalosiphum_maidis_corn_leaf_aphid_Insecta_Aphididae_XP_026816943.1
Chelonus_insularis_wasps_ants_and_bees_Insecta_Braconidae_XP_034936581.1
Atta_colombica_ants_Insecta_Formicidae_XP_018058633.1
Bombyx_mori_domestic_silkworm_Insecta_Bombycidae_XP_004925143.1
Nicrophorus_vespilloides_beetles_Insecta_Silphidae_XP_017779772.1
Trichogramma_pretiosum_wasps_ants_and_bees_Insecta_Trichogrammatidae_XP_014220926.1
Copidosoma_floridanum_wasps_ants_and_bees_Insecta_Encyrtidae_XP_014220016.1
Culicidae 1 7
obscura subgroup 1 3
Drosophila_bipectinata_flies_Insecta_Drosophilidae_XP_017095299.1
Drosophila_kikkawai_flies_Insecta_Drosophilidae_XP_017028811.1
melanogaster group 1 6
melanogaster subgroup 1 4
Drosophila_erecta_flies_Insecta_Drosophilidae_XP_001971522.1
melanogaster subgroup 2 2
Homo_sapiens_human_Dipnotetrapodomorpha_Hominidae_XP_005262724.1
Mus_musculus_house_mouse_Dipnotetrapodomorpha_Muridae_XP_006503925.1
0.50

Figure S10

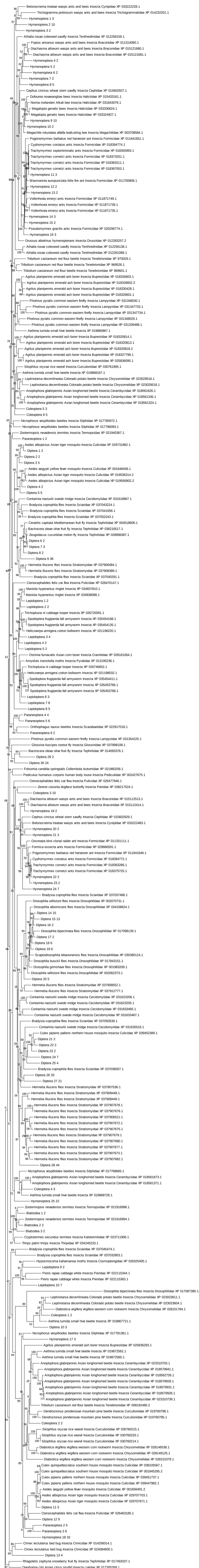

Figure S11

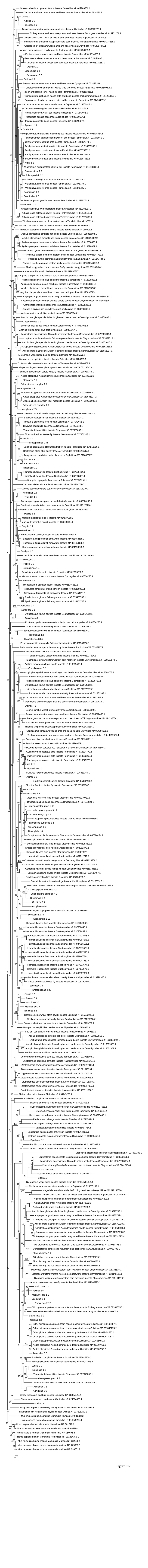

Figure S12

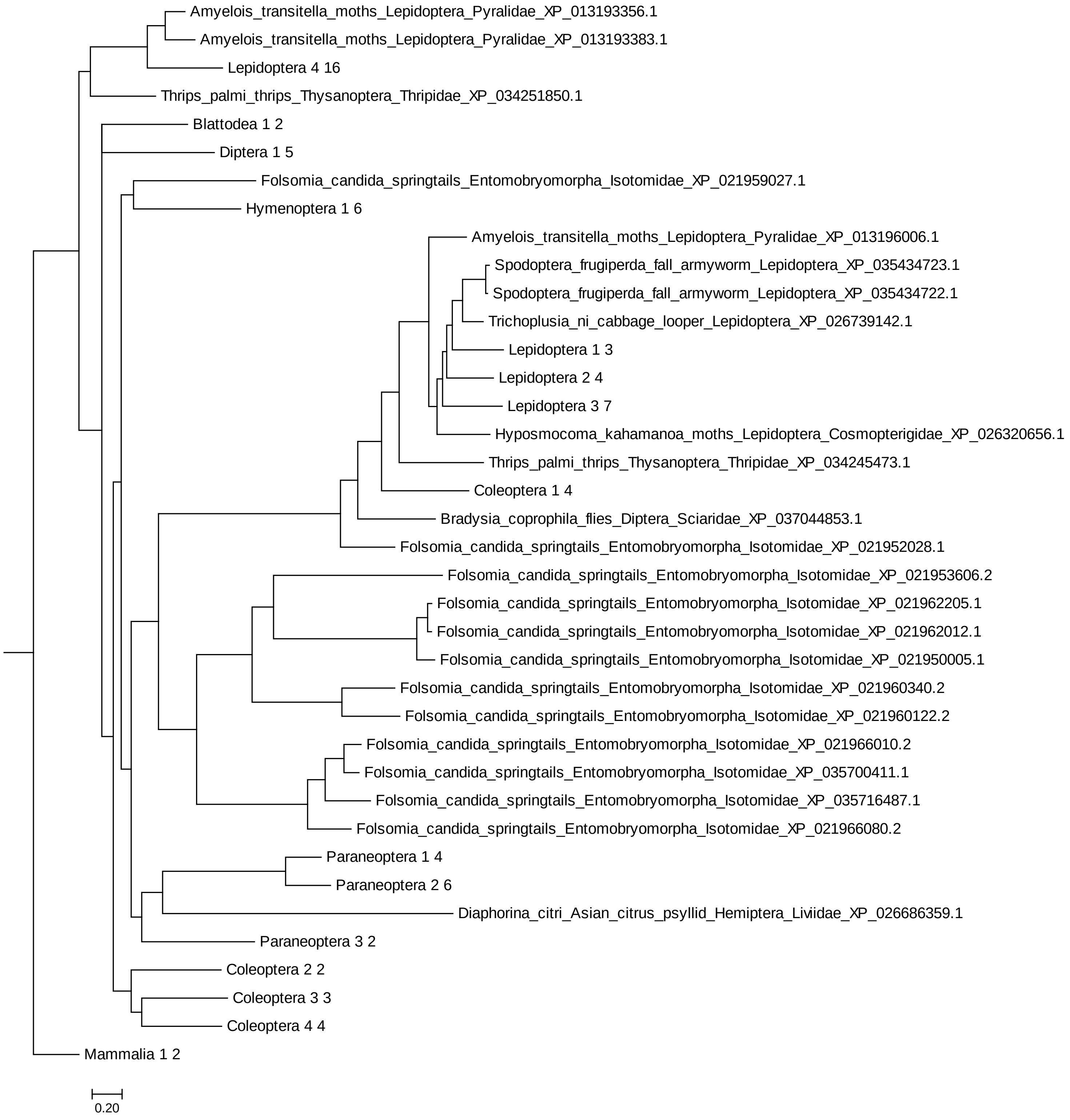
Amyelois_transitella_moths_Lepidoptera_Pyralidae_XP_013193356.1
Amyelois_transitella_moths_Lepidoptera_Pyralidae_XP_013193383.1
Lepidoptera 4 16
Thrips_palmi_thrips_Thysanoptera_Thripidae_XP_034251850.1
Blattodea 1 2
Diptera 1 5
Folsomia_candida_springtails_Entomobryomorpha_Isotomidae_XP_021959027.1
Hymenoptera 1 6
Amyelois_transitella_moths_Lepidoptera_Pyralidae_XP_013196006.1
Spodoptera_frugiperda_fall_armyworm_Lepidoptera_XP_035434723.1
Spodoptera_frugiperda_fall_armyworm_Lepidoptera_XP_035434722.1
Trichoplusia_ni_cabbage_looper_Lepidoptera_XP_026739142.1
Lepidoptera 1 3
Lepidoptera 2 4
Lepidoptera 3 7
Hyposmocoma_kahamanoa_moths_Lepidoptera_Cosmopterigidae_XP_026320656.1
Thrips_palmi_thrips_Thysanoptera_Thripidae_XP_034245473.1
Coleoptera 1 4
Bradysia_coprophila_flies_Diptera_Sciaridae_XP_037044853.1
Folsomia_candida_springtails_Entomobryomorpha_Isotomidae_XP_021952028.1
Folsomia_candida_springtails_Entomobryomorpha_Isotomidae_XP_021953606.2
Folsomia_candida_springtails_Entomobryomorpha_Isotomidae_XP_021962205.1
Folsomia_candida_springtails_Entomobryomorpha_Isotomidae_XP_021962012.1
Folsomia_candida_springtails_Entomobryomorpha_Isotomidae_XP_021950005.1
Folsomia_candida_springtails_Entomobryomorpha_Isotomidae_XP_021960340.2
Folsomia_candida_springtails_Entomobryomorpha_Isotomidae_XP_021960122.2
Folsomia_candida_springtails_Entomobryomorpha_Isotomidae_XP_021966010.2
Folsomia_candida_springtails_Entomobryomorpha_Isotomidae_XP_035700411.1
Folsomia_candida_springtails_Entomobryomorpha_Isotomidae_XP_035716487.1
Folsomia_candida_springtails_Entomobryomorpha_Isotomidae_XP_021966080.2
Paraneoptera 1 4
Paraneoptera 2 6
Diaphorina_citri_Asian_citrus_psyllid_Hemiptera_Liviidae_XP_026686359.1
Paraneoptera 3 2
Coleoptera 2 2
Coleoptera 3 3
Coleoptera 4 4
Mammalia 1 2
0.20

Figure S13

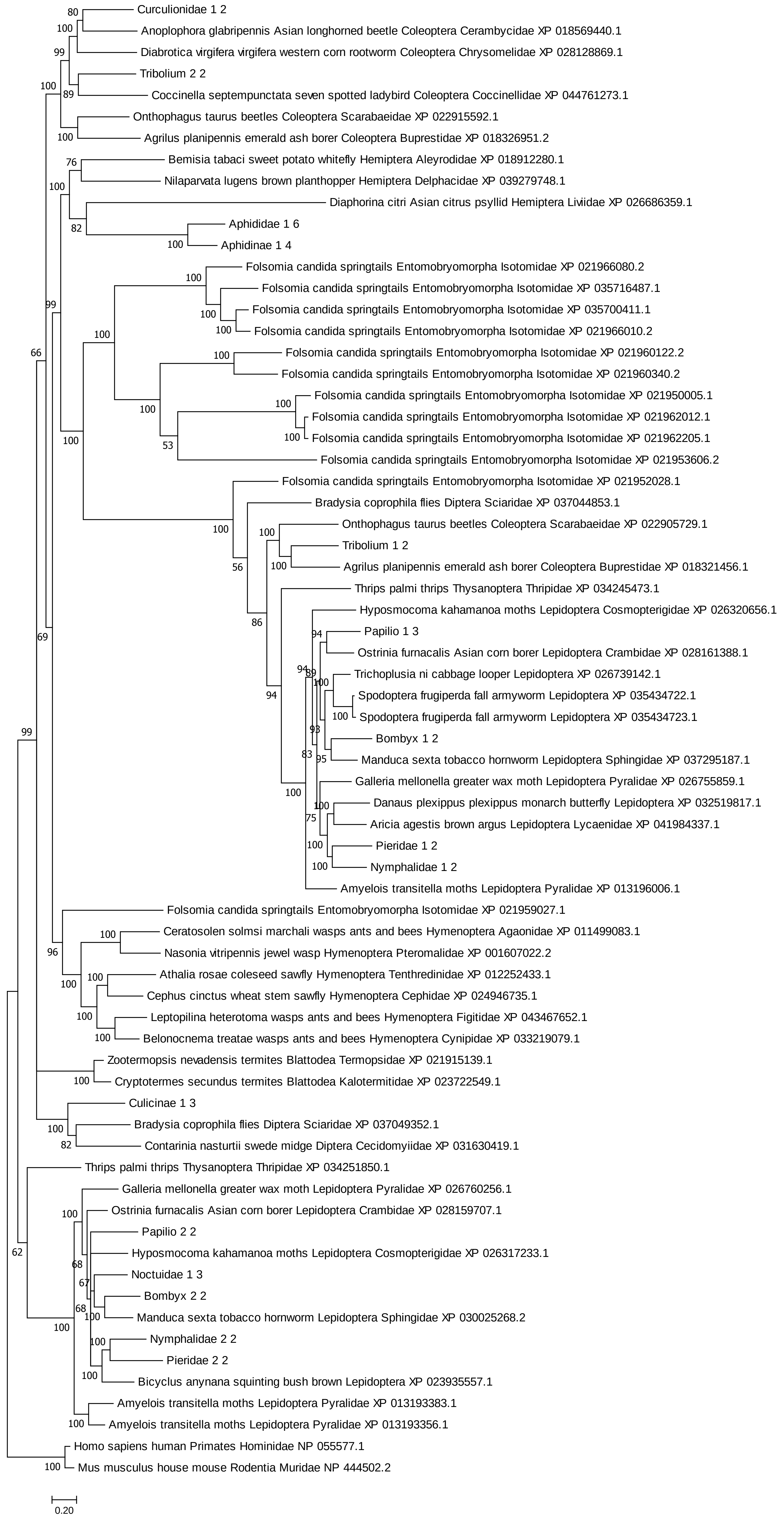
Curculionidae 1 2
Anoplophora glabripennis Asian longhorned beetle Coleoptera Cerambycidae XP 018569440.1
Diabrotica virgifera virgifera western corn rootworm Coleoptera Chrysomelidae XP 028128869.1
Tribolium 2 2
Coccinella septempunctata seven spotted ladybird Coleoptera Coccinellidae XP 044761273.1
Onthophagus taurus beetles Coleoptera Scarabaeidae XP 022915592.1
Agrilus planipennis emerald ash borer Coleoptera Buprestidae XP 018326951.2
Bemisia tabaci sweet potato whitefly Hemiptera Aleyrodidae XP 018912280.1
Nilaparvata lugens brown planthopper Hemiptera Delphacidae XP 039279748.1
Diaphorina citri Asian citrus psyllid Hemiptera Liviidae XP 026686359.1
Aphididae 1 6
Aphidinae 1 4
Folsomia candida springtails Entomobryomorpha Isotomidae XP 021966080.2
Folsomia candida springtails Entomobryomorpha Isotomidae XP 035716487.1
Folsomia candida springtails Entomobryomorpha Isotomidae XP 035700411.1
Folsomia candida springtails Entomobryomorpha Isotomidae XP 021966010.2
Folsomia candida springtails Entomobryomorpha Isotomidae XP 021960122.2
Folsomia candida springtails Entomobryomorpha Isotomidae XP 021960340.2
Folsomia candida springtails Entomobryomorpha Isotomidae XP 021950005.1
Folsomia candida springtails Entomobryomorpha Isotomidae XP 021962012.1
Folsomia candida springtails Entomobryomorpha Isotomidae XP 021962205.1
Folsomia candida springtails Entomobryomorpha Isotomidae XP 021953606.2
Folsomia candida springtails Entomobryomorpha Isotomidae XP 021952028.1
Bradysia coprophila flies Diptera Sciaridae XP 037044853.1
Onthophagus taurus beetles Coleoptera Scarabaeidae XP 022905729.1
Tribolium 1 2
Agrilus planipennis emerald ash borer Coleoptera Buprestidae XP 018321456.1
Thrips palmi thrips Thysanoptera Thripidae XP 034245473.1
Hyposmocoma kahamanoa moths Lepidoptera Cosmopterigidae XP 026320656.1
Papilio 1 3
Ostrinia furnacalis Asian corn borer Lepidoptera Crambidae XP 028161388.1
Trichoplusia ni cabbage looper Lepidoptera XP 026739142.1
Spodoptera frugiperda fall armyworm Lepidoptera XP 035434722.1
Spodoptera frugiperda fall armyworm Lepidoptera XP 035434723.1
Bombyx 1 2
Manduca sexta tobacco hornworm Lepidoptera Sphingidae XP 037295187.1
Galleria mellonella greater wax moth Lepidoptera Pyralidae XP 026755859.1
Danaus plexippus plexippus monarch butterfly Lepidoptera XP 032519817.1
Aricia agestis brown argus Lepidoptera Lycaenidae XP 041984337.1
Pieridae 1 2
Nymphalidae 1 2
Amyelois transitella moths Lepidoptera Pyralidae XP 013196006.1
Folsomia candida springtails Entomobryomorpha Isotomidae XP 021959027.1
Ceratosolen solmsi marchali wasps ants and bees Hymenoptera Agaonidae XP 011499083.1
Nasonia vitripennis jewel wasp Hymenoptera Pteromalidae XP 001607022.2
Athalia rosae coleseed sawfly Hymenoptera Tenthredinidae XP 012252433.1
Cephus cinctus wheat stem sawfly Hymenoptera Cephidae XP 024946735.1
Leptopilina heterotoma wasps ants and bees Hymenoptera Figitidae XP 043467652.1
Belonocnema treatae wasps ants and bees Hymenoptera Cynipidae XP 033219079.1
Zootermopsis nevadensis termites Blattodea Termopsidae XP 021915139.1
Cryptotermes secundus termites Blattodea Kalotermitidae XP 023722549.1
Culicinae 1 3
Bradysia coprophila flies Diptera Sciaridae XP 037049352.1
Contarinia nasturtii swede midge Diptera Cecidomyiidae XP 031630419.1
Thrips palmi thrips Thysanoptera Thripidae XP 034251850.1
Galleria mellonella greater wax moth Lepidoptera Pyralidae XP 026760256.1
Ostrinia furnacalis Asian corn borer Lepidoptera Crambidae XP 028159707.1
Papilio 2 2
Hyposmocoma kahamanoa moths Lepidoptera Cosmopterigidae XP 026317233.1
Noctuidae 1 3
Bombyx 2 2
Manduca sexta tobacco hornworm Lepidoptera Sphingidae XP 030025268.2
Nymphalidae 2 2
Pieridae 2 2
Bicyclus anynana squinting bush brown Lepidoptera XP 023935557.1
Amyelois transitella moths Lepidoptera Pyralidae XP 013193383.1
Amyelois transitella moths Lepidoptera Pyralidae XP 013193356.1
Homo sapiens human Primates Hominidae NP 055577.1
Mus musculus house mouse Rodentia Muridae NP 444502.2
0.20

Figure S14
