# Supplementary material for: Inferences on the evolution of the ascorbic acid synthesis pathway in insects using Phylogenetic Tree Collapser (PTC), a tool for the automated collapsing of phylogenetic trees using taxonomic information
Source: J Integr Bioinform. 2024 Jul 24;21(2):20230051. doi: 10.1515/jib-2023-0051 (PMC11377030; doi:10.1515/jib-2023-0051)
Supplement: Supplementary file 1 — Supplementary Material Details [file j_jib-2023-0051_suppl_001.zip › Supplementary_File_10_UGP2.con_PDF.pdf]

```
#NEXUS

[ID: 6408711078]
begin taxa;
	dimensions ntax=159;
	taxlabels
		Drosophila_hydei_flies_Insecta_Drosophilidae_XP_023178712.2
		Drosophila_yakuba_flies_Insecta_Drosophilidae_XP_015049447.1
		Fopius_arisanus_wasps_ants_and_bees_Insecta_Braconidae_XP_011300568.1
		Teleopsis_dalmanni_flies_Insecta_Diopsidae_XP_037940363.1
		Trichogramma_pretiosum_wasps_ants_and_bees_Insecta_Trichogrammatidae_XP_014229789.1
		Pseudomyrmex_gracilis_ants_Insecta_Formicidae_XP_020298835.1
		Cyphomyrmex_costatus_ants_Insecta_Formicidae_XP_018398292.1
		Sitophilus_oryzae_rice_weevil_Insecta_Curculionidae_XP_030765094.1
		Atta_cephalotes_ants_Insecta_Formicidae_XP_012056589.1
		Apis_dorsata_giant_honeybee_Insecta_Apidae_XP_006608699.1
		Vespa_mandarinia_Asian_giant_hornet_Insecta_Vespidae_XP_035725177.1
		Drosophila_albomicans_flies_Insecta_Drosophilidae_XP_034110876.1
		Papilio_xuthus_Asian_swallowtail_Insecta_Papilionidae_XP_013173498.1
		Maniola_hyperantus_ringlet_Insecta_XP_034836142.1
		Cimex_lectularius_bed_bug_Insecta_Cimicidae_XP_014251469.1
		Apis_mellifera_honey_bee_Insecta_Apidae_XP_006568568.1
		Drosophila_melanogaster_fruit_fly_Insecta_Drosophilidae_NP_001163399.1
		Drosophila_sechellia_flies_Insecta_Drosophilidae_XP_032575790.1
		Vollenhovia_emeryi_ants_Insecta_Formicidae_XP_011872903.1
		Drosophila_novamexicana_flies_Insecta_Drosophilidae_XP_030570830.1
		Spodoptera_frugiperda_fall_armyworm_Insecta_XP_035452866.1
		Scaptodrosophila_lebanonensis_flies_Insecta_Drosophilidae_XP_030373408.1
		Pieris_rapae_cabbage_white_Insecta_Pieridae_XP_022123025.1
		Atta_colombica_ants_Insecta_Formicidae_XP_018053896.1
		Rhagoletis_zephyria_snowberry_fruit_fly_Insecta_Tephritidae_XP_017460796.1
		Rhagoletis_zephyria_snowberry_fruit_fly_Insecta_Tephritidae_XP_017490037.1
		Drosophila_navojoa_flies_Insecta_Drosophilidae_XP_017956458.1
		Acyrthosiphon_pisum_pea_aphid_Insecta_Aphididae_XP_008186898.1
		Acyrthosiphon_pisum_pea_aphid_Insecta_Aphididae_NP_001153814.1
		Anopheles_coluzzii_mosquitos_Insecta_Culicidae_XP_040226373.1
		Drosophila_mauritiana_flies_Insecta_Drosophilidae_XP_033158601.1
		Megachile_rotundata_alfalfa_leafcutting_bee_Insecta_Megachilidae_XP_003705526.1
		Trachymyrmex_cornetzi_ants_Insecta_Formicidae_XP_018361844.1
		Agrilus_planipennis_emerald_ash_borer_Insecta_Buprestidae_XP_018319603.1
		Anoplophora_glabripennis_Asian_longhorned_beetle_Insecta_Cerambycidae_XP_018575662.1
		Drosophila_serrata_flies_Insecta_Drosophilidae_XP_020817134.1
		Anopheles_albimanus_mosquitos_Insecta_Culicidae_XP_035777774.1
		Ooceraea_biroi_clonal_raider_ant_Insecta_Formicidae_XP_011346949.1
		Drosophila_rhopaloa_flies_Insecta_Drosophilidae_XP_016971638.1
		Ceratina_calcarata_bees_Insecta_Apidae_XP_017888851.1
		Melanaphis_sacchari_aphids_Insecta_Aphididae_XP_025200047.1
		Diuraphis_noxia_Russian_wheat_aphid_Insecta_Aphididae_XP_015371727.1
		Nomia_melanderi_Alkali_bee_Insecta_Halictidae_XP_031829852.1
		Drosophila_eugracilis_flies_Insecta_Drosophilidae_XP_017085243.1
		Osmia_bicornis_bicornis_red_mason_bee_Insecta_Megachilidae_XP_029035107.1
		Lucilia_cuprina_Australian_sheep_blowfly_Insecta_Calliphoridae_XP_023307777.1
		Drosophila_biarmipes_flies_Insecta_Drosophilidae_XP_016958606.1
		Drosophila_erecta_flies_Insecta_Drosophilidae_XP_015013253.1
		Drosophila_simulans_flies_Insecta_Drosophilidae_XP_016031154.1
		Nicrophorus_vespilloides_beetles_Insecta_Silphidae_XP_017784124.1
		Thrips_palmi_thrips_Insecta_Thripidae_XP_034248703.1
		Thrips_palmi_thrips_Insecta_Thripidae_XP_034234671.1
		Bactrocera_latifrons_flies_Insecta_Tephritidae_XP_018804140.1
		Bombyx_mori_domestic_silkworm_Insecta_Bombycidae_XP_004925791.1
		Drosophila_suzukii_flies_Insecta_Drosophilidae_XP_016934410.1
		Cryptotermes_secundus_termites_Insecta_Kalotermitidae_XP_023706496.1
		Bradysia_coprophila_flies_Insecta_Sciaridae_XP_037037114.1
		Anopheles_stephensi_Asian_malaria_mosquito_Insecta_Culicidae_XP_035895526.1
		Zootermopsis_nevadensis_termites_Insecta_Termopsidae_XP_021926972.1
```

66 Copidosoma_floridanum_wasps_ants_and_bees_Insecta_Encyrtidae_XP_023246482.1
67 Bactrocera_dorsalis_oriental_fruit_fly_Insecta_Tephritidae_XP_011201671.1
68 Eufriesea_mexicana_bees_Insecta_Apidae_XP_017759602.1
69 Homo_sapiens_human_Dipnotetrapodomorpha_Hominidae_NP_006750.3
70 Papilio_machaon_common_yellow_swallowtail_Insecta_Papilionidae_XP_014357932.1
71 Pogonomyrmex_barbatus_red_harvester_ant_Insecta_Formicidae_XP_011633863.2
72 Drosophila_miranda_flies_Insecta_Drosophilidae_XP_017136674.1
73 Pediculus_humanus_corporis_human_body_louse_Insecta_Pediculidae_XP_002427960.1
74 Ceratosolen_solmsi_marchali_wasps_ants_and_bees_Insecta_Agaonidae_XP_011505453.1
75 Aedes_aegypti_yellow_fever_mosquito_Insecta_Culicidae_XP_021709650.1
76 Temnothorax_curvispinosus_ants_Insecta_Formicidae_XP_024875189.1
77 Danaus_plexippus_plexippus_monarch_butterfly_Insecta_XP_032522728.1
78 Drosophila_takahashii_flies_Insecta_Drosophilidae_XP_017005504.1
79 Chelonus_insularis_wasps_ants_and_bees_Insecta_Braconidae_XP_034950805.1
80 Formica_exsecta_ants_Insecta_Formicidae_XP_029668907.1
81 Rhopalosiphum_maidis_corn_leaf_aphid_Insecta_Aphididae_XP_026806423.1
82 Drosophila_ananassae_flies_Insecta_Drosophilidae_XP_014764726.1
83 Ceratitis_capitata_Mediterranean_fruit_fly_Insecta_Tephritidae_XP_004525568.1
84 Bombus_bifarius_bees_Insecta_Apidae_XP_033320330.1
85 Athalia_rosae_coleseed_sawfly_Insecta_Tenthredinidae_XP_012254496.1
86 Drosophila_busckii_flies_Insecta_Drosophilidae_XP_033149476.1
87 Megalopta_genalis_bees_Insecta_Halictidae_XP_033327637.1
88 Rhagoletis_pomonella_apple_maggot_Insecta_Tephritidae_XP_036337980.1
89 Rhagoletis_pomonella_apple_maggot_Insecta_Tephritidae_XP_036337988.1
90 Drosophila_subpulchrella_flies_Insecta_Drosophilidae_XP_037718116.1
91 Nylanderia_fulva_ants_Insecta_Formicidae_XP_029158886.1
92 Linepithema_humile_Argentine_ant_Insecta_Formicidae_XP_012233216.1
93 Osmia_lignaria_orchard_mason_bee_Insecta_Megachilidae_XP_034185632.1
94 Bombus_impatiens_common_eastern_bumble_bee_Insecta_Apidae_XP_012246165.1
95 Belonocnema_treatae_wasps_ants_and_bees_Insecta_Cynipidae_XP_033207281.1
96 Amyelois_transitella_moths_Insecta_Pyralidae_XP_013191066.1
97 Bicyclus_anynana_squinting_bush_brown_Insecta_XP_023953994.1
98 Musca_domestica_house_fly_Insecta_Muscidae_XP_005178509.1
99 Bombus_vosnesenskii_bees_Insecta_Apidae_XP_033355449.1
100 Dufourea_novaeangliae_bees_Insecta_Halictidae_XP_015428827.1
101 Glossina_fuscipes_tsetse_fly_Insecta_Glossinidae_XP_037890285.1
102 Trachymyrmex_septentrionalis_ants_Insecta_Formicidae_XP_018349678.1
103 Manduca_sexta_tobacco_hornworm_Insecta_Sphingidae_XP_030025930.1
104 Drosophila_subobscura_flies_Insecta_Drosophilidae_XP_034655381.1
105 Dendroctonus_ponderosae_mountain_pine_beetle_Insecta_Curculionidae_XP_019767521.1
106 Anopheles_arabiensis_mosquitos_Insecta_Culicidae_XP_040175343.1
107 Wasmannia_auropunctata_little_fire_ant_Insecta_Formicidae_XP_011691853.1
108 Harpegnathos_saltator_Jerdon_s_jumping_ant_Insecta_Formicidae_XP_025162682.1
109 Contarinia_nasturtii_swede_midge_Insecta_Cecidomyiidae_XP_031626256.1
110 Drosophila_elegans_flies_Insecta_Drosophilidae_XP_017122019.1
111 Drosophila_pseudoobscura_flies_Insecta_Drosophilidae_XP_015042491.1
112 Bombus_terrestris_buff_tailed_bumblebee_Insecta_Apidae_XP_012173187.1
113 Monomorium_pharaonis_pharaoh_ant_Insecta_Formicidae_XP_012529385.1
114 Hermetia_illucens_flies_Insecta_Stratiomyidae_XP_037904968.1
115 Ostrinia_furnacalis_Asian_corn_borer_Insecta_Crambidae_XP_028160524.1
116 Drosophila_persimilis_flies_Insecta_Drosophilidae_XP_026849460.1
117 Vanessa_tameamea_butterflies_Insecta_XP_026500095.1
118 Aphis_gossypii_cotton_aphid_Insecta_Aphididae_XP_027836620.1
119 Camponotus_floridanus_Florida_carpenter_ant_Insecta_Formicidae_XP_011267731.1
120 Bactrocera_tryoni_Queensland_fruit_fly_Insecta_Tephritidae_XP_039963640.1
121 Microplitis_demolitor_wasps_ants_and_bees_Insecta_Braconidae_XP_008558395.1
122 Polistes_canadensis_wasps_ants_and_bees_Insecta_Vespidae_XP_014606336.1
123 Photinus_pyralis_common_eastern_firefly_Insecta_Lampyridae_XP_031348028.1
124 Zerene_cesonia_dogface_butterfly_Insecta_Pieridae_XP_038221254.1
125 Folsomia_candida_springtails_Collembola_Isotomidae_XP_021951716.1
126 Orussus_abietinus_hymenopterans_Insecta_Orussidae_XP_012284624.1

```
		Drosophila_obscura_flies_Insecta_Drosophilidae_XP_022213818.1
		Drosophila_ficusphila_flies_Insecta_Drosophilidae_XP_017053403.1
		Solenopsis_invicta_red_fire_ant_Insecta_Formicidae_XP_011169660.1
		Apis_florea_little_honeybee_Insecta_Apidae_XP_012341511.1
		Diachasma_alloeum_wasps_ants_and_bees_Insecta_Braconidae_XP_015119005.1
		Trichoplusia_ni_cabbage_looper_Insecta_XP_026738640.1
		Helicoverpa_armigera_cotton_bollworm_Insecta_XP_021191029.1
		Culex_pipiens_pallens_northern_house_mosquito_Insecta_Culicidae_XP_039437936.1
		Bactrocera_oleae_olive_fruit_fly_Insecta_Tephritidae_XP_036226870.1
		Drosophila_bipectinata_flies_Insecta_Drosophilidae_XP_017101471.1
		Mus_musculus_house_mouse_Dipnotetrapodomorpha_Muridae_NP_647458.1
		Onthophagus_taurus_beetles_Insecta_Scarabaeidae_XP_022909765.1
		Myzus_persicae_green_peach_aphid_Insecta_Aphididae_XP_022181825.1
		Dinoponera_quadriceps_ants_Insecta_Formicidae_XP_014479406.1
		Bemisia_tabaci_sweet_potato_whitefly_Insecta_Aleyrodidae_XP_018899458.1
		Aethina_tumida_small_hive_beetle_Insecta_XP_019881433.1
		Nasonia_vitripennis_jewel_wasp_Insecta_Pteromalidae_XP_001606199.1
		Cephus_cinctus_wheat_stem_sawfly_Insecta_Cephidae_XP_015598067.1
		Drosophila_kikkawai_flies_Insecta_Drosophilidae_XP_017028486.1
		Drosophila_mojavensis_flies_Insecta_Drosophilidae_XP_015017821.1
		Sipha_flava_yellow_sugarcane_aphid_Insecta_Aphididae_XP_025408720.1
		Drosophila_grimshawi_flies_Insecta_Drosophilidae_XP_001984129.1
		Tribolium_castaneum_red_flour_beetle_Insecta_Tenebrionidae_XP_008195624.1
		Drosophila_santomea_flies_Insecta_Drosophilidae_XP_039486073.1
		Habropoda_laboriosa_bees_Insecta_Apidae_XP_017798052.1
		Anopheles_gambiae_str._PEST_African_malaria_mosquito_Insecta_Culicidae_XP_003435790.1
		Polistes_dominula_European_paper_wasp_Insecta_Vespidae_XP_015185668.1
		Papilio_polytes_common_Mormon_Insecta_Papilionidae_XP_013147348.1
		Zeugodacus_cucurbitae_melon_fly_Insecta_Tephritidae_XP_011196158.1
		Drosophila_arizonae_flies_Insecta_Drosophilidae_XP_017861488.1
		Lucilia_sericata_common_green_bottle_fly_Insecta_Calliphoridae_XP_037815600.1
		Acromyrmex_echinatior_Panamanian_leafcutter_ant_Insecta_Formicidae_XP_011066558.1
		Drosophila_guanche_flies_Insecta_Drosophilidae_XP_034133434.1
		Drosophila_willistoni_flies_Insecta_Drosophilidae_XP_015033065.1
		Aedes_albopictus_Asian_tiger_mosquito_Insecta_Culicidae_XP_019534747.2
		Culex_quinquefasciatus_southern_house_mosquito_Insecta_Culicidae_XP_001847482.1
		Odontomachus_brunneus_ants_Insecta_Formicidae_XP_032683898.1
		Pararge_aegeria_specked_wood_butterfly_Insecta_XP_039759906.1
		Drosophila_virilis_flies_Insecta_Drosophilidae_XP_015030693.1
		;
end;
begin trees;
	translate
		1	Drosophila_hydei_flies_Insecta_Drosophilidae_XP_023178712.2,
		2	Drosophila_yakuba_flies_Insecta_Drosophilidae_XP_015049447.1,
		3	Fopius_arisanus_wasps_ants_and_bees_Insecta_Braconidae_XP_011300568.1,
		4	Teleopsis_dalmanni_flies_Insecta_Diopsidae_XP_037940363.1,
		5	Trichogramma_pretiosum_wasps_ants_and_bees_Insecta_Trichogrammatidae_XP_014229789.1,
		6	Pseudomyrmex_gracilis_ants_Insecta_Formicidae_XP_020298835.1,
		7	Cyphomyrmex_costatus_ants_Insecta_Formicidae_XP_018398292.1,
		8	Sitophilus_oryzae_rice_weevil_Insecta_Curculionidae_XP_030765094.1,
		9	Atta_cephalotes_ants_Insecta_Formicidae_XP_012056589.1,
		10	Apis_dorsata_giant_honeybee_Insecta_Apidae_XP_006608699.1,
		11	Vespa_mandarinia_Asian_giant_hornet_Insecta_Vespidae_XP_035725177.1,
		12	Drosophila_albomicans_flies_Insecta_Drosophilidae_XP_034110876.1,
		13	Papilio_xuthus_Asian_swallowtail_Insecta_Papilionidae_XP_013173498.1,
		14	Maniola_hyperantus_ringlet_Insecta_XP_034836142.1,
		15	Cimex_lectularius_bed_bug_Insecta_Cimicidae_XP_014251469.1,
		16	Apis_mellifera_honey_bee_Insecta_Apidae_XP_006568568.1,
		17	Drosophila_melanogaster_fruit_fly_Insecta_Drosophilidae_NP_001163399.1,
		18	Drosophila_sechellia_flies_Insecta_Drosophilidae_XP_032575790.1,
```

```
→→19→Vollenhovia_emeryi_ants_Insecta_Formicidae_XP_011872903.1,LF
→→20→Drosophila_novamexicana_flies_Insecta_Drosophilidae_XP_030570830.1,LF
→→21→Spodoptera_frugiperda_fall_armyworm_Insecta_XP_035452866.1,LF
→→22→Scaptodrosophila_lebanonensis_flies_Insecta_Drosophilidae_XP_030373408.1,LF
→→23→Pieris_rapae_cabbage_white_Insecta_Pieridae_XP_022123025.1,LF
→→24→Atta_colombica_ants_Insecta_Formicidae_XP_018053896.1,LF
→→25→Rhagoletis_zephyria_snowberry_fruit_fly_Insecta_Tephritidae_XP_017460796.1,LF
→→26→Rhagoletis_zephyria_snowberry_fruit_fly_Insecta_Tephritidae_XP_017490037.1,LF
→→27→Drosophila_navojoa_flies_Insecta_Drosophilidae_XP_017956458.1,LF
→→28→Acyrthosiphon_pisum_pea_aphid_Insecta_Aphididae_XP_008186898.1,LF
→→29→Acyrthosiphon_pisum_pea_aphid_Insecta_Aphididae_NP_001153814.1,LF
→→30→Anopheles_coluzzii_mosquitos_Insecta_Culicidae_XP_040226373.1,LF
→→31→Drosophila_mauritiana_flies_Insecta_Drosophilidae_XP_033158601.1,LF
→→32→Megachile_rotundata_alfalfa_leafcutting_bee_Insecta_Megachilidae_XP_003705526.1,LF
→→33→Trachymyrmex_cornetzi_ants_Insecta_Formicidae_XP_018361844.1,LF
→→34→Agrilus_planipennis_emerald_ash_borer_Insecta_Buprestidae_XP_018319603.1,LF
→→35→Anoplophora_glabripennis_Asian_longhorned_beetle_Insecta_Cerambycidae_XP_018575662.1,LF
→→36→Drosophila_serrata_flies_Insecta_Drosophilidae_XP_020817134.1,LF
→→37→Anopheles_albimanus_mosquitos_Insecta_Culicidae_XP_035777774.1,LF
→→38→Ooceraea_biroi_clonal_raider_ant_Insecta_Formicidae_XP_011346949.1,LF
→→39→Drosophila_rhopaloa_flies_Insecta_Drosophilidae_XP_016971638.1,LF
→→40→Ceratina_calcarata_bees_Insecta_Apidae_XP_017888851.1,LF
→→41→Melanaphis_sacchari_aphids_Insecta_Aphididae_XP_025200047.1,LF
→→42→Diuraphis_noxia_Russian_wheat_aphid_Insecta_Aphididae_XP_015371727.1,LF
→→43→Nomia_melanderi_Alkali_bee_Insecta_Halictidae_XP_031829852.1,LF
→→44→Drosophila_eugracilis_flies_Insecta_Drosophilidae_XP_017085243.1,LF
→→45→Osmia_bicornis_bicornis_red_mason_bee_Insecta_Megachilidae_XP_029035107.1,LF
→→46→Lucilia_cuprina_Australian_sheep_blowfly_Insecta_Calliphoridae_XP_023307777.1,LF
→→47→Drosophila_biarmipes_flies_Insecta_Drosophilidae_XP_016958606.1,LF
→→48→Drosophila_erecta_flies_Insecta_Drosophilidae_XP_015013253.1,LF
→→49→Drosophila_simulans_flies_Insecta_Drosophilidae_XP_016031154.1,LF
→→50→Nicrophorus_vespilloides_beetles_Insecta_Silphidae_XP_017784124.1,LF
→→51→Thrips_palmi_thrips_Insecta_Thripidae_XP_034248703.1,LF
→→52→Thrips_palmi_thrips_Insecta_Thripidae_XP_034234671.1,LF
→→53→Bactrocera_latifrons_flies_Insecta_Tephritidae_XP_018804140.1,LF
→→54→Bombyx_mori_domestic_silkworm_Insecta_Bombycidae_XP_004925791.1,LF
→→55→Drosophila_suzukii_flies_Insecta_Drosophilidae_XP_016934410.1,LF
→→56→Cryptotermes_secundus_termites_Insecta_Kalotermitidae_XP_023706496.1,LF
→→57→Bradysia_coprophila_flies_Insecta_Sciaridae_XP_037037114.1,LF
→→58→Anopheles_stephensi_Asian_malaria_mosquito_Insecta_Culicidae_XP_035895526.1,LF
→→59→Zootermopsis_nevadensis_termites_Insecta_Termopsidae_XP_021926972.1,LF
→→60→Copidosoma_floridanum_wasps_ants_and_bees_Insecta_Encyrtidae_XP_023246482.1,LF
→→61→Bactrocera_dorsalis_oriental_fruit_fly_Insecta_Tephritidae_XP_011201671.1,LF
→→62→Eufriesea_mexicana_bees_Insecta_Apidae_XP_017759602.1,LF
→→63→Homo_sapiens_human_Dipnotetrapodomorpha_Hominidae_NP_006750.3,LF
→→64→Papilio_machaon_common_yellow_swallowtail_Insecta_Papilionidae_XP_014357932.1,LF
→→65→Pogonomyrmex_barbatus_red_harvester_ant_Insecta_Formicidae_XP_011633863.2,LF
→→66→Drosophila_miranda_flies_Insecta_Drosophilidae_XP_017136674.1,LF
→→67→Pediculus_humanus_corporis_human_body_louse_Insecta_Pediculidae_XP_002427960.1,LF
→→68→Ceratosolen_solmsi_marchali_wasps_ants_and_bees_Insecta_Agaonidae_XP_011505453.1,LF
→→69→Aedes_aegypti_yellow_fever_mosquito_Insecta_Culicidae_XP_021709650.1,LF
→→70→Temnothorax_curvispinosus_ants_Insecta_Formicidae_XP_024875189.1,LF
```

```
→→71→Danaus_plexippus_plexippus_monarch_butterfly_Insecta_XP_032522728.1,LF
→→72→Drosophila_takahashii_flies_Insecta_Drosophilidae_XP_017005504.1,LF
→→73→Chelonus_insularis_wasps_ants_and_bees_Insecta_Braconidae_XP_034950805.1,LF
→→74→Formica_exsecta_ants_Insecta_Formicidae_XP_029668907.1,LF
→→75→Rhopalosiphum_maidis_corn_leaf_aphid_Insecta_Aphididae_XP_026806423.1,LF
→→76→Drosophila_ananassae_flies_Insecta_Drosophilidae_XP_014764726.1,LF
→→77→Ceratitis_capitata_Mediterranean_fruit_fly_Insecta_Tephritidae_XP_004525568.1,LF
→→78→Bombus_bifarius_bees_Insecta_Apidae_XP_033320330.1,LF
→→79→Athalia_rosae_coleseed_sawfly_Insecta_Tenthredinidae_XP_012254496.1,LF
→→80→Drosophila_busckii_flies_Insecta_Drosophilidae_XP_033149476.1,LF
→→81→Megalopta_genalis_bees_Insecta_Halictidae_XP_033327637.1,LF
→→82→Rhagoletis_pomonella_apple_maggot_Insecta_Tephritidae_XP_036337980.1,LF
→→83→Rhagoletis_pomonella_apple_maggot_Insecta_Tephritidae_XP_036337988.1,LF
→→84→Drosophila_subpulchrella_flies_Insecta_Drosophilidae_XP_037718116.1,LF
→→85→Nylanderia_fulva_ants_Insecta_Formicidae_XP_029158886.1,LF
→→86→Linepithema_humile_Argentine_ant_Insecta_Formicidae_XP_012233216.1,LF
→→87→Osmia_lignaria_orchard_mason_bee_Insecta_Megachilidae_XP_034185632.1,LF
→→88→Bombus_impatiens_common_eastern_bumble_bee_Insecta_Apidae_XP_012246165.1,LF
→→89→Belonocnema_treatae_wasps_ants_and_bees_Insecta_Cynipidae_XP_033207281.1,LF
→→90→Amyelois_transitella_moths_Insecta_Pyralidae_XP_013191066.1,LF
→→91→Bicyclus_anynana_squinting_bush_brown_Insecta_XP_023953994.1,LF
→→92→Musca_domestica_house_fly_Insecta_Muscidae_XP_005178509.1,LF
→→93→Bombus_vosnesenskii_bees_Insecta_Apidae_XP_033355449.1,LF
→→94→Dufourea_novaeangliae_bees_Insecta_Halictidae_XP_015428827.1,LF
→→95→Glossina_fuscipes_tsetse_fly_Insecta_Glossinidae_XP_037890285.1,LF
→→96→Trachymyrmex_septentrionalis_ants_Insecta_Formicidae_XP_018349678.1,LF
→→97→Manduca_sexta_tobacco_hornworm_Insecta_Sphingidae_XP_030025930.1,LF
→→98→Drosophila_subobscura_flies_Insecta_Drosophilidae_XP_034655381.1,LF
→→99→Dendroctonus_ponderosae_mountain_pine_beetle_Insecta_Curculionidae_XP_019767521.1,LF
→→100→Anopheles_arabiensis_mosquitos_Insecta_Culicidae_XP_040175343.1,LF
→→101→Wasmannia_auropunctata_little_fire_ant_Insecta_Formicidae_XP_011691853.1,LF
→→102→Harpegnathos_saltator_Jerdon_s_jumping_ant_Insecta_Formicidae_XP_025162682.1,LF
→→103→Contarinia_nasturtii_swede_midge_Insecta_Cecidomyiidae_XP_031626256.1,LF
→→104→Drosophila_elegans_flies_Insecta_Drosophilidae_XP_017122019.1,LF
→→105→Drosophila_pseudoobscura_flies_Insecta_Drosophilidae_XP_015042491.1,LF
→→106→Bombus_terrestris_buff_tailed_bumblebee_Insecta_Apidae_XP_012173187.1,LF
→→107→Monomorium_pharaonis_pharaoh_ant_Insecta_Formicidae_XP_012529385.1,LF
→→108→Hermetia_illucens_flies_Insecta_Stratiomyidae_XP_037904968.1,LF
→→109→Ostrinia_furnacalis_Asian_corn_borer_Insecta_Crambidae_XP_028160524.1,LF
→→110→Drosophila_persimilis_flies_Insecta_Drosophilidae_XP_026849460.1,LF
→→111→Vanessa_tameamea_butterflies_Insecta_XP_026500095.1,LF
→→112→Aphis_gossypii_cotton_aphid_Insecta_Aphididae_XP_027836620.1,LF
→→113→Camponotus_floridanus_Florida_carpenter_ant_Insecta_Formicidae_XP_011267731.1,LF
→→114→Bactrocera_tryoni_Queensland_fruit_fly_Insecta_Tephritidae_XP_039963640.1,LF
115→Microplitis_demolitor_wasps_ants_and_bees_Insecta_Braconidae_XP_008558395.1,LF
→→116→Polistes_canadensis_wasps_ants_and_bees_Insecta_Vespidae_XP_014606336.1,LF
→→117→Photinus_pyralis_common_eastern_firefly_Insecta_Lampyridae_XP_031348028.1,LF
→→118→Zerene_cesonia_dogface_butterfly_Insecta_Pieridae_XP_038221254.1,LF
→→119→Folsomia_candida_springtails_Collembola_Isotomidae_XP_021951716.1,LF
→→120→Orussus_abietinus_hymenopterans_Insecta_Orussidae_XP_012284624.1,LF
→→121→Drosophila_obscura_flies_Insecta_Drosophilidae_XP_022213818.1,LF
→→122→Drosophila_ficusphila_flies_Insecta_Drosophilidae_XP_017053403.1,LF
→→123→Solenopsis_invicta_red_fire_ant_Insecta_Formicidae_XP_011169660.1,LF
→→124→Apis_florea_little_honeybee_Insecta_Apidae_XP_012341511.1,LF
→→125→Diachasma_alloeum_wasps_ants_and_bees_Insecta_Braconidae_XP_015119005.1,LF
→→126→Trichoplusia_ni_cabbage_looper_Insecta_XP_026738640.1,LF
→→127→Helicoverpa_armigera_cotton_bollworm_Insecta_XP_021191029.1,LF
→→128→
```

```
Culex_pipiens_pallens_northern_house_mosquito_Insecta_Culicidae_XP_039437936.1,
		129	Bactrocera_oleae_olive_fruit_fly_Insecta_Tephritidae_XP_036226870.1,
		130	Drosophila_bipectinata_flies_Insecta_Drosophilidae_XP_017101471.1,
		131	Mus_musculus_house_mouse_Dipnotetrapodomorpha_Muridae_NP_647458.1,
		132	Onthophagus_taurus_beetles_Insecta_Scarabaeidae_XP_022909765.1,
		133	Myzus_persicae_green_peach_aphid_Insecta_Aphididae_XP_022181825.1,
		134	Dinoponera_quadriceps_ants_Insecta_Formicidae_XP_014479406.1,
		135	Bemisia_tabaci_sweet_potato_whitefly_Insecta_Aleyrodidae_XP_018899458.1,
		136	Aethina_tumida_small_hive_beetle_Insecta_XP_019881433.1,
		137	Nasonia_vitripennis_jewel_wasp_Insecta_Pteromalidae_XP_001606199.1,
		138	Cephus_cinctus_wheat_stem_sawfly_Insecta_Cephidae_XP_015598067.1,
		139	Drosophila_kikkawai_flies_Insecta_Drosophilidae_XP_017028486.1,
		140	Drosophila_mojavensis_flies_Insecta_Drosophilidae_XP_015017821.1,
		141	Sipha_flava_yellow_sugarcane_aphid_Insecta_Aphididae_XP_025408720.1,
		142	Drosophila_grimshawi_flies_Insecta_Drosophilidae_XP_001984129.1,
		143	Tribolium_castaneum_red_flour_beetle_Insecta_Tenebrionidae_XP_008195624.1,
		144	Drosophila_santomea_flies_Insecta_Drosophilidae_XP_039486073.1,
		145	Habropoda_laboriosa_bees_Insecta_Apidae_XP_017798052.1,
		146	Anopheles_gambiae_str._PEST_African_malaria_mosquito_Insecta_Culicidae_XP_003435790.1,
		147	Polistes_dominula_European_paper_wasp_Insecta_Vespidae_XP_015185668.1,
		148	Papilio_polytes_common_Mormon_Insecta_Papilionidae_XP_013147348.1,
		149	Zeugodacus_cucurbitae_melon_fly_Insecta_Tephritidae_XP_011196158.1,
		150	Drosophila_arizonae_flies_Insecta_Drosophilidae_XP_017861488.1,
		151	Lucilia_sericata_common_green_bottle_fly_Insecta_Calliphoridae_XP_037815600.1,
		152	Acromyrmex_echinatior_Panamanian_leafcutter_ant_Insecta_Formicidae_XP_011066558.1,
		153	Drosophila_guanche_flies_Insecta_Drosophilidae_XP_034133434.1,
		154	Drosophila_willistoni_flies_Insecta_Drosophilidae_XP_015033065.1,
		155	Aedes_albopictus_Asian_tiger_mosquito_Insecta_Culicidae_XP_019534747.2,
		156	Culex_quinquefasciatus_southern_house_mosquito_Insecta_Culicidae_XP_001847482.1,
		157	Odontomachus_brunneus_ants_Insecta_Formicidae_XP_032683898.1,
		158	Pararge_aegeria_specked_wood_butterfly_Insecta_XP_039759906.1,
		159	Drosophila_virilis_flies_Insecta_Drosophilidae_XP_015030693.1
		;
   [Note: This tree contains information on the topology, 
          branch lengths (if present), and the probability
          of the partition indicated by the branch.]
   tree con_50_majrule = (1:0.0657787,(27:0.02117463,(140:0.007609639,150:0.008623101)1.000:0.01509481)1.000:0.04273493,(((((((((((((2:0.006601177,144:0.004632995)1.000:0.02013764,48:0.0276354)1.000:0.01122303,(17:0.02107317,(18:0.01303996,(31:0.006905337,49:0.00992081)0.516:0.0024341)1.000:0.008205118)1.000:0.01069832)1.000:0.06625725,((47:0.02641525,(55:0.01973351,84:0.008739523)1.000:0.01829109)1.000:0.01351527,72:0.05271124)1.000:0.0168893)0.992:0.01205779,39:0.06708798,(44:0.1113872,122:0.06463681)0.636:0.008419946)0.597:0.009232086,104:0.04247433)1.000:0.0276514,(76:0.02910409,130:0.04653392)1.000:0.06907836)0.978:0.01595202,(36:0.04039739,139:0.04058114)1.000:0.04032075)1.000:0.06372527,(((66:0.005896397,(105:0.004963274,110:0.005383807)1.000:0.006309706)1.000:0.06669772,121:0.02601648)1.000:0.01964468,(98:0.03086452,153:0.01846951)1.000:0.02087522)1.000:0.07958018)0.995:0.03594286,(((((((((((((((3:0.1440993,125:0.2330026)1.000:0.4427141,(73:0.2442269,115:0.265137)1.000:0.2890393)1.000:0.3509091,((((5:0.4590355,((60:0.3589348,68:0.3260729)0.683:0.07550416,137:0.2766136)0.834:0.07793061)1.000:0.2909802,89:0.5486636)1.000:0.1464328,((((((6:0.2062305,86:0.1481852)0.988:0.03591195,((((((7:0.05917219,((((9:0.007940232,24:0.004211631)1.000:0.02559282,152:0.01361971)1.000:0.008436702,96:0.01883794)0.974:0.005631434,33:0.01820879)1.000:0.0288024)1.000:0.06112682,101:0.06148263)1.000:0.02149437,(19:0.08827367,70:0.04451495)0.999:0.0161762)0.999:0.01786041,(107:0.08679522,123:0.04988241)1.000:0.03496571)1.000:0.03313255,65:0.1495376)1.000:0.04263419,38:0.1639141)0.569:0.01200913)0.874:0.02368124,((74:0.04611052,113:0.09094274)1.000:0.03408386,85:0.07272525)1.000:0.05717731)1.000:0.07307499,((102:0.08768841,157:0.08919829)0.596:0.01424795,134:0.08542058)1.000:0.05029479)1.000:0.2541316,((((((((10:0.01369493,16:0.01840823)0.814:0.01041273,124:0.01119216)1.000:0.07549003,62:0.0804432)0.792:0.02607449,(((78:7.045653E-4,93:0.002626257)0.995:0.002621651,88:0.002512576)0.993:0.006492723,106:0.01093569)1.000:0.09353716)1.000:0.04248882,145:0.1108907)0.980:0.02494238,40:0.3778342)0.996:0.03486453,(32:0.08343512,(45:0.005499742,87:0.004110683)1.000:0.0974338
```

```
    2)1.000:0.07604319)0.935:0.03823317,((43:0.1502872,81:0.1927049)0.985:0.03709952,94
    :0.1325538)1.000:0.06683523)1.000:0.1411924)0.998:0.07650734,(11:0.09090334,(116:0.
    02214571,147:0.03946852)1.000:0.04256823)1.000:0.308102)1.000:0.1380025)0.958:0.068
    74108,120:0.6585696)0.589:0.03876118)0.988:0.05892734,138:0.4848531)0.878:0.0566689
    6,79:0.4512672)1.000:0.2402693,((((((((8:0.4892849,99:0.5912626)1.000:0.139739,132
    :0.7684381)0.968:0.07864673,35:0.6414739)0.657:0.05990821,117:0.6079684)0.740:0.072
    2875,136:0.5071429)0.578:0.05166173,143:0.4426521)0.760:0.05285254,(34:0.6994537,50
    :0.5811089)0.751:0.1199426)1.000:0.123926,((((28:0.05745823,((((41:0.05242745,75:0.
    04046876)0.578:0.005494339,112:0.06128346)1.000:0.03083596,42:0.04209378)0.624:0.00
    964598,133:0.05485759)0.996:0.02425035)0.865:0.02109519,29:0.01495096)1.000:0.12694
    15,141:0.1778768)1.000:0.675804,135:0.681301)0.988:0.1217914)0.524:0.03110681,((15:
    0.842446,119:0.8753722)1.000:0.1978812,((63:0.061047,131:0.07499675)1.000:1.391574,
    67:0.8561689)0.985:0.1711961)0.875:0.07598083)0.990:0.06752224)1.000:0.1792792,(56:
    0.1043502,59:0.1719067)1.000:0.3029224)0.993:0.10084,(25:1.020129,51:0.3536266)0.95
    6:0.1409031)0.732:0.02987963,(((((((13:0.07605337,64:0.05634414)1.000:0.04601575,14
    8:0.08399694)1.000:0.1777712,((54:0.3281847,109:0.1941417)0.738:0.04347226,97:0.218
    8926)0.816:0.02888443)0.795:0.01578744,((21:0.1581301,127:0.1937701)1.000:0.0557342
    ,126:0.1327458)1.000:0.06577546)0.830:0.03208194,(((14:0.1797515,(91:0.1877753,158:
    0.1464976)1.000:0.06604159)1.000:0.07405605,(71:0.2920092,111:0.2098348)0.987:0.058
    93012)1.000:0.06245821,(23:0.2634754,118:0.1626194)1.000:0.08473444)0.900:0.0309424
    2)0.769:0.03502747,90:0.2299283)1.000:0.3225834,52:0.6498841)0.947:0.1035077)1.000:
    0.2126387,(((((30:0.002480472,146:0.003125657)0.773:0.002729262,100:0.00423795)1.00
    0:0.06003595,58:0.09147681)1.000:0.06048621,37:0.1514716)1.000:0.06775858,((69:0.16
    31471,155:0.1149392)1.000:0.1692317,(128:0.01303172,156:0.0277157)1.000:0.1306377)1
    .000:0.06545697)1.000:0.1153087)1.000:0.1321073,(57:0.4276476,103:0.4921112)0.994:0
    .08890668)1.000:0.1013732,108:0.4365114)1.000:0.1561661,((4:0.2917916,(((46:0.05501
    136,151:0.01848129)1.000:0.1676104,92:0.1628226)0.940:0.0415339,95:0.3822785)0.998:
    0.04818525)0.996:0.0686409,((((26:0.01570907,82:0.01828857)1.000:0.01956677,83:0.00
    2553783)1.000:0.1622156,((((53:0.02395456,61:0.03029109)0.896:0.005846779,114:0.021
    59051)1.000:0.04621781,129:0.07227771)1.000:0.02061156,149:0.06073583)1.000:0.05579
    659)0.954:0.03565924,77:0.1201236)1.000:0.1459391)1.000:0.1032979)1.000:0.1170644,2
    2:0.154886)1.000:0.0387578,154:0.2356313)1.000:0.05760825)0.737:0.02559572,(12:0.11
    89217,80:0.1771734)0.825:0.03001797)0.999:0.02904732,142:0.0855883)1.000:0.02325451
    ,(20:0.01981259,159:0.01219629)1.000:0.05743935)1.000:0.0425396);
end;
```
