# Supplementary material for: Inferences on the evolution of the ascorbic acid synthesis pathway in insects using Phylogenetic Tree Collapser (PTC), a tool for the automated collapsing of phylogenetic trees using taxonomic information
Source: J Integr Bioinform. 2024 Jul 24;21(2):20230051. doi: 10.1515/jib-2023-0051 (PMC11377030; doi:10.1515/jib-2023-0051)
Supplement: Supplementary file 1 — Supplementary Material Details [file j_jib-2023-0051_suppl_001.zip › Supplementary_File_2_Stop_terms_PDF.pdf]

BlattodeaLF
ParaneopteraLF
ColeopteraLF
LepidopteraLF
DipteraLF
CollembolaLF
HymenopteraLF
MammaliaLF
SiphonapteraLF
