# Supplementary material for: Inferences on the evolution of the ascorbic acid synthesis pathway in insects using Phylogenetic Tree Collapser (PTC), a tool for the automated collapsing of phylogenetic trees using taxonomic information
Source: J Integr Bioinform. 2024 Jul 24;21(2):20230051. doi: 10.1515/jib-2023-0051 (PMC11377030; doi:10.1515/jib-2023-0051)
Supplement: Supplementary file 1 — Supplementary Material Details [file j_jib-2023-0051_suppl_001.zip › Supplementary_File_3_Commands_used_PDF.pdf]

```bash
#!/bin/bash

DATA_DIR=/your/data/dir

if [ ! -d "${DATA_DIR}" ]; then
    echo "Error: the specified data directory (${DATA_DIR}) does not exist."
    echo 'Did you set the DATA_DIR variable?'
fi

mkdir -p "${DATA_DIR}/out"

#####################################
#                                   #
#           Gene: AKR1B1            #
#                                   #
#####################################

#
# Case_1
#

docker run --rm -it \
    -v /var/run/docker.sock:/var/run/docker.sock \
    -v "${DATA_DIR}:/data" -v ~/.ptc-cache:/ptc-cache \
    pegi3s/phylogenetic-tree-collapser collapse-tree.py \
        --input /data/Supplementary_file_4_AKR1B1_tree \
        --input-format newick \
        --output /data/out/Supplementary_file_4_AKR1B1_tree_Case_1.nwk \
        --output-type phylogram \
        --output-collapsed-nodes /data/out/Supplementary_file_4_AKR1B1_tree_Case_1.tsv \
        --input-path-host "${DATA_DIR}"

#
# Case_2
#

docker run --rm -it \
    -v /var/run/docker.sock:/var/run/docker.sock \
    -v "${DATA_DIR}:/data" -v ~/.ptc-cache:/ptc-cache \
    pegi3s/phylogenetic-tree-collapser collapse-tree.py \
        --input /data/Supplementary_file_4_AKR1B1_tree \
        --input-format newick \
        --taxonomy-stop-terms /data/Supplementary_file_2_Stop_terms \
        --output /data/out/Supplementary_file_4_AKR1B1_tree_Case_2.nwk \
        --output-type phylogram \
        --output-collapsed-nodes /data/out/Supplementary_file_4_AKR1B1_tree_Case_2.tsv \
        --input-path-host "${DATA_DIR}" \
        --flatten-taxonomy-with-stop-terms

#####################################
#                                   #
#         Remaining genes           #
#                                   #
#####################################

#
# Put in "GENES" the list of genes to analyze:
# - Supplementary_file_5_DHR24
# - Supplementary_file_6_PGM1
# - Supplementary_file_7_PGM2
# - Supplementary_file_8_UGDH_Pri
# - Supplementary_file_9_UGDH_Sec
# - Supplementary_file_10_UGP2
#

GENES="Supplementary_file_5_DHR24 Supplementary_file_6_PGM1 Supplementary_file_7_PGM2 Supplementary_file_8_UGDH_Pri Supplementary_file_9_UGDH_Sec Supplementary_file_10_UGP2"

# GENES="Supplementary_file_5_DHR24 Supplementary_file_6_PGM1"

```

```
for GENE in $(echo ${GENES} | tr ' ' '\n'); do
    #
    # Case_1
    #

    docker run --rm -it \
        -v /var/run/docker.sock:/var/run/docker.sock \
        -v "${DATA_DIR}:/data" -v ~/.ptc-cache:/ptc-cache \
        pegi3s/phylogenetic-tree-collapser collapse-tree.py \
            --input /data/${GENE}.con \
            --input-format nexus \
            --output /data/out/${GENE}.con.Case_1.nwk \
            --output-type phylogram \
            --output-collapsed-nodes /data/out/${GENE}.con.Case_1.tsv \
            --input-path-host "${DATA_DIR}"

    #
    # Case_2
    #

    docker run --rm -it \
        -v /var/run/docker.sock:/var/run/docker.sock \
        -v "${DATA_DIR}:/data" -v ~/.ptc-cache:/ptc-cache \
        pegi3s/phylogenetic-tree-collapser collapse-tree.py \
            --input /data/${GENE}.con \
            --input-format nexus \
            --taxonomy-stop-terms /data/Supplementary_file_2_Stop_terms \
            --output /data/out/${GENE}.con.Case_2.nwk \
            --output-type phylogram \
            --output-collapsed-nodes /data/out/${GENE}.con.Case_2.tsv \
            --input-path-host "${DATA_DIR}" \
            --flatten-taxonomy-with-stop-terms
done
```
