# Supplementary material for: Inferences on the evolution of the ascorbic acid synthesis pathway in insects using Phylogenetic Tree Collapser (PTC), a tool for the automated collapsing of phylogenetic trees using taxonomic information
Source: J Integr Bioinform. 2024 Jul 24;21(2):20230051. doi: 10.1515/jib-2023-0051 (PMC11377030; doi:10.1515/jib-2023-0051)
Supplement: Supplementary file 1 — Supplementary Material Details [file j_jib-2023-0051_suppl_001.zip › Supplementary_File_4_AKR1B1_tree_PDF.pdf]

((((Orussus_abietinus_hymenopterans_Insecta_Orussidae_XP_012283356.1:0.22666,Diachasma
_alloeum_wasps_ants_and_bees_Insecta_Braconidae_XP_015114231.1:0.46710)0.976:0.09901,(
((Nomia_melanderi_Alkali_bee_Insecta_Halictidae_XP_031843075.1:0.20229,Dufourea_novaea
ngliae_bees_Insecta_Halictidae_XP_015433138.1:0.19347)0.998:0.12783,((Osmia_bicornis_b
icornis_red_mason_bee_Insecta_Megachilidae_XP_029056468.1:0.03842,Osmia_lignaria_orcha
rd_mason_bee_Insecta_Megachilidae_XP_034179280.1:0.02087)1.000:0.13998,(Ceratina_calca
rata_bees_Insecta_Apidae_XP_017889598.1:0.22345,(Bombus_impatiens_common_eastern_bumbl
e_bee_Insecta_Apidae_XP_033176022.1:0.00062,(Bombus_terrestris_buff_tailed_bumblebee_I
nsecta_Apidae_XP_020722624.1:0.02487,(Bombus_vosnesenskii_bees_Insecta_Apidae_XP_03336
1947.1:0.00124,(Bombus_bifarius_bees_Insecta_Apidae_XP_033317103.1:0.00124,Bombus_vanc
ouverensis_nearcticus_bees_Insecta_Apidae_XP_033203326.1:0.00064)0.909:0.00245)0.806:0
.00119)0.646:0.00221)1.000:0.21983)0.995:0.09882)0.079:0.02880)1.000:0.23013,(Belonocn
ema_treatae_wasps_ants_and_bees_Insecta_Cynipidae_XP_033222228.1:0.36098,(Trichogramma
_pretiosum_wasps_ants_and_bees_Insecta_Trichogrammatidae_XP_014232201.1:0.73713,(Cerat
osolen_solmsi_marchali_wasps_ants_and_bees_Insecta_Agaonidae_XP_011500527.1:0.26407,(T
richogramma_pretiosum_wasps_ants_and_bees_Insecta_Trichogrammatidae_XP_014237688.1:0.1
9519,Copidosoma_floridanum_wasps_ants_and_bees_Insecta_Encyrtidae_XP_014204972.1:0.235
16)0.943:0.06155)0.893:0.06734)0.994:0.15222)0.968:0.09351)0.087:0.02285)0.979:0.07007
,((Athalia_rosae_coleseed_sawfly_Insecta_Tenthredinidae_XP_012256159.1:0.27936,((((((C
helonus_insularis_wasps_ants_and_bees_Insecta_Braconidae_XP_034940264.1:0.22022,Microp
litis_demolitor_wasps_ants_and_bees_Insecta_Braconidae_XP_008549774.1:0.27969)1.000:0.
22404,((Chelonus_insularis_wasps_ants_and_bees_Insecta_Braconidae_XP_034940265.1:0.190
25,Microplitis_demolitor_wasps_ants_and_bees_Insecta_Braconidae_XP_008549770.1:0.20500
)0.997:0.10815,((Fopius_arisanus_wasps_ants_and_bees_Insecta_Braconidae_XP_011314057.1
:0.15061,Diachasma_alloeum_wasps_ants_and_bees_Insecta_Braconidae_XP_015121682.1:0.079
52)1.000:0.28393,(Fopius_arisanus_wasps_ants_and_bees_Insecta_Braconidae_XP_011314060.
1:0.12226,(Diachasma_alloeum_wasps_ants_and_bees_Insecta_Braconidae_XP_015121680.1:0.0
6170,Diachasma_alloeum_wasps_ants_and_bees_Insecta_Braconidae_XP_015121681.1:0.17376)0
.904:0.04590)1.000:0.15625)0.529:0.06529)0.590:0.03476)0.428:0.03142,(Fopius_arisanus_
wasps_ants_and_bees_Insecta_Braconidae_XP_011307499.1:0.11210,Diachasma_alloeum_wasps_
ants_and_bees_Insecta_Braconidae_XP_015114250.1:0.09186)1.000:0.20954)0.995:0.08628,(B
elonocnema_treatae_wasps_ants_and_bees_Insecta_Cynipidae_XP_033223159.1:0.25270,(Cerat
osolen_solmsi_marchali_wasps_ants_and_bees_Insecta_Agaonidae_XP_011500526.1:0.35013,(N
asonia_vitripennis_jewel_wasp_Insecta_Pteromalidae_NP_001153411.1:0.17337,(Trichogramm
a_pretiosum_wasps_ants_and_bees_Insecta_Trichogrammatidae_XP_014232551.1:0.16925,Copid
osoma_floridanum_wasps_ants_and_bees_Insecta_Encyrtidae_XP_014204959.1:0.19971)0.340:0
.04987)0.417:0.03727)0.967:0.09204)0.434:0.07211)0.458:0.03933,(Cephus_cinctus_wheat_s
tem_sawfly_Insecta_Cephidae_XP_015602927.1:0.26550,(((((Dufourea_novaeangliae_bees_Ins
ecta_Halictidae_XP_015433161.1:0.11629,(Nomia_melanderi_Alkali_bee_Insecta_Halictidae_
XP_031843076.1:0.10523,(Megalopta_genalis_bees_Insecta_Halictidae_XP_033330624.1:0.079
69,Megalopta_genalis_bees_Insecta_Halictidae_XP_033324927.1:0.03579)1.000:0.08543)0.89
8:0.03273)1.000:0.06674,(Habropoda_laboriosa_bees_Insecta_Apidae_XP_017792917.1:0.1170
6,((Bombus_terrestris_buff_tailed_bumblebee_Insecta_Apidae_XP_012172367.1:0.01370,(Bom
bus_impatiens_common_eastern_bumble_bee_Insecta_Apidae_XP_003484790.1:0.00356,(Bombus_
bifarius_bees_Insecta_Apidae_XP_033317096.1:0.00064,Bombus_vancouverensis_nearcticus_b
ees_Insecta_Apidae_XP_033203324.1:0.00123)0.769:0.00131)0.946:0.01246)0.999:0.05196,(E
ufriesea_mexicana_bees_Insecta_Apidae_XP_017764961.1:0.12838,((Apis_mellifera_honey_be
e_Insecta_Apidae_XP_624353.1:0.01922,Apis_cerana_Asiatic_honeybee_Insecta_Apidae_XP_01
6909705.1:0.00706)0.276:0.00352,(Apis_dorsata_giant_honeybee_Insecta_Apidae_XP_0066203
37.1:0.02545,Apis_florea_little_honeybee_Insecta_Apidae_XP_031773914.1:0.04339)0.243:0
.00693)1.000:0.13631)0.954:0.03463)0.929:0.02630)0.984:0.03654)0.948:0.04005,(Osmia_bi
cornis_bicornis_red_mason_bee_Insecta_Megachilidae_XP_029056467.1:0.01192,Osmia_lignar
ia_orchard_mason_bee_Insecta_Megachilidae_XP_034179279.1:0.01649)1.000:0.06455)0.000:0
.03692,Megachile_rotundata_alfalfa_leafcutting_bee_Insecta_Megachilidae_XP_003708584.1
:0.09668)1.000:0.12132,((Dinoponera_quadriceps_ants_Insecta_Formicidae_XP_014485092.1:
0.07416,(Harpegnathos_saltator_Jerdon_s_jumping_ant_Insecta_Formicidae_XP_011148177.1:
0.11807,Odontomachus_brunneus_ants_Insecta_Formicidae_XP_032672316.1:0.09428)0.402:0.0
1498)1.000:0.13187,(((Ooceraea_biroi_clonal_raider_ant_Insecta_Formicidae_XP_011331106
.1:0.17292,Linepithema_humile_Argentine_ant_Insecta_Formicidae_XP_012219929.1:0.08844)
0.958:0.03580,((Pogonomyrmex_barbatus_red_harvester_ant_Insecta_Formicidae_XP_01164165
1.1:0.16834,((((Cyphomyrmex_costatus_ants_Insecta_Formicidae_XP_018394774.1:0.06039,((
Trachymyrmex_septentrionalis_ants_Insecta_Formicidae_XP_018355993.1:0.03664,(Trachymyr
mex_cornetzi_ants_Insecta_Formicidae_XP_018373251.1:0.03266,(Trachymyrmex_cornetzi_ant
s_Insecta_Formicidae_XP_018363211.1:0.01637,Trachymyrmex_cornetzi_ants_Insecta_Formici
dae_XP_018367053.1:0.01570)0.699:0.00544)0.399:0.00411)0.485:0.00262,(Acromyrmex_echin
atior_Panamanian_leafcutter_ant_Insecta_Formicidae_XP_011055126.1:0.02869,(Atta_cephal
otes_ants_Insecta_Formicidae_XP_012062916.1:0.00727,Atta_colombica_ants_Insecta_Formic
idae_XP_018044275.1:0.00369)1.000:0.02728)0.885:0.00639)0.916:0.01441)1.000:0.07464,Wa
smannia_auropunctata_little_fire_ant_Insecta_Formicidae_XP_011700808.1:0.08022)0.824:0
.01852,((Monomorium_pharaonis_pharaoh_ant_Insecta_Formicidae_XP_012539616.1:0.09734,So
lenopsis_invicta_red_fire_ant_Insecta_Formicidae_XP_025989498.1:0.08509)0.978:0.03179,
(Monomorium_pharaonis_pharaoh_ant_Insecta_Formicidae_XP_012539617.1:0.10138,Solenopsis
_invicta_red_fire_ant_Insecta_Formicidae_XP_011170042.2:0.10660)1.000:0.06240)0.101:0.

01700)0.876:0.01276,(Vollenhovia_emeryi_ants_Insecta_Formicidae_XP_011871749.1:0.08915
,(Vollenhovia_emeryi_ants_Insecta_Formicidae_XP_011871739.1:0.01701,Vollenhovia_emeryi
_ants_Insecta_Formicidae_XP_011871735.1:0.02277)1.000:0.13603)0.929:0.02311)0.988:0.03
868)0.949:0.02727,(Formica_exsecta_ants_Insecta_Formicidae_XP_029670341.1:0.05973,(Nyl
anderia_fulva_ants_Insecta_Formicidae_XP_029157253.1:0.11323,Camponotus_floridanus_Flo
rida_carpenter_ant_Insecta_Formicidae_XP_019882849.1:0.06195)0.516:0.01051)1.000:0.072
76)0.412:0.01025)0.549:0.01370,Pseudomyrmex_gracilis_ants_Insecta_Formicidae_XP_020295
774.1:0.15709)0.737:0.03104)1.000:0.12434)0.976:0.07076)0.403:0.03659)0.294:0.03604,Or
ussus_abietinus_hymenopterans_Insecta_Orussidae_XP_012283257.2:0.28004)0.964:0.06867)0
.987:0.08680,(Athalia_rosae_coleseed_sawfly_Insecta_Tenthredinidae_XP_012256138.1:0.11
901,Athalia_rosae_coleseed_sawfly_Insecta_Tenthredinidae_XP_012261089.1:0.11277)1.000:
0.37306)0.689:0.04204)0.994:0.07812,(Tribolium_castaneum_red_flour_beetle_Insecta_Tene
brionidae_XP_970029.1:0.55543,(Tribolium_castaneum_red_flour_beetle_Insecta_Tenebrioni
dae_XP_969526.1:0.16943,Tribolium_castaneum_red_flour_beetle_Insecta_Tenebrionidae_XP_
969601.1:0.29579)0.887:0.07888)0.983:0.09359,(((((Zootermopsis_nevadensis_termites_Ins
ecta_Termopsidae_XP_021940367.1:0.30751,(Nilaparvata_lugens_brown_planthopper_Insecta_
Delphacidae_XP_022196473.1:0.33193,Bemisia_tabaci_sweet_potato_whitefly_Insecta_Aleyro
didae_XP_018917746.1:0.41024)0.603:0.05308)0.936:0.05855,(((((Anopheles_albimanus_mosq
uitos_Insecta_Culicidae_XP_035789842.1:0.10481,(Anopheles_stephensi_Asian_malaria_mosq
uito_Insecta_Culicidae_XP_035893342.1:0.07460,(Anopheles_coluzzii_mosquitos_Insecta_Cu
licidae_XP_040231319.1:0.00277,(Anopheles_arabiensis_mosquitos_Insecta_Culicidae_XP_04
0161422.1:0.00823,Anopheles_gambiae_str._PEST_African_malaria_mosquito_Insecta_Culicid
ae_XP_308086.3:0.00350)0.860:0.00432)1.000:0.09313)0.969:0.04159)0.989:0.05075,((Culex
_pipiens_pallens_northern_house_mosquito_Insecta_Culicidae_XP_039450000.1:0.00723,Cule
x_quinquefasciatus_southern_house_mosquito_Insecta_Culicidae_XP_038111824.1:0.02575)1.
000:0.12820,(Aedes_albopictus_Asian_tiger_mosquito_Insecta_Culicidae_XP_029731882.1:0.
00489,(Aedes_albopictus_Asian_tiger_mosquito_Insecta_Culicidae_XP_029731786.1:0.01896,
Aedes_aegypti_yellow_fever_mosquito_Insecta_Culicidae_XP_001648463.1:0.17051)0.847:0.0
3540)1.000:0.16461)0.951:0.05179)0.998:0.09829,(((Anopheles_gambiae_str._PEST_African_
malaria_mosquito_Insecta_Culicidae_XP_308085.4:0.01638,(Anopheles_coluzzii_mosquitos_I
nsecta_Culicidae_XP_040231421.1:0.01306,Anopheles_arabiensis_mosquitos_Insecta_Culicid
ae_XP_040161420.1:0.01070)0.806:0.00524)0.992:0.07228,(Anopheles_stephensi_Asian_malar
ia_mosquito_Insecta_Culicidae_XP_035893333.1:0.10062,Anopheles_albimanus_mosquitos_Ins
ecta_Culicidae_XP_035789841.1:0.17160)0.099:0.04363)1.000:0.14048,((Culex_pipiens_pall
ens_northern_house_mosquito_Insecta_Culicidae_XP_039449999.1:0.02438,Culex_quinquefasc
iatus_southern_house_mosquito_Insecta_Culicidae_XP_001844838.2:0.03763)1.000:0.18338,(
Aedes_aegypti_yellow_fever_mosquito_Insecta_Culicidae_XP_001648458.1:0.11042,(Aedes_al
bopictus_Asian_tiger_mosquito_Insecta_Culicidae_XP_019536314.2:0.04576,Aedes_albopictu
s_Asian_tiger_mosquito_Insecta_Culicidae_XP_019556902.2:0.07495)0.924:0.03820)1.000:0.
15668)0.988:0.08775)1.000:0.14178)0.995:0.13364,((Contarinia_nasturtii_swede_midge_Ins
ecta_Cecidomyiidae_XP_031618867.1:0.32791,(Bradysia_coprophila_flies_Insecta_Sciaridae
_XP_037043224.1:0.33641,(Bradysia_coprophila_flies_Insecta_Sciaridae_XP_037041656.1:0.
30155,Bradysia_coprophila_flies_Insecta_Sciaridae_XP_037052243.1:0.21699)0.566:0.06187
)0.994:0.11633)0.955:0.07906,(((Teleopsis_dalmanni_flies_Insecta_Diopsidae_XP_03793569
3.1:0.26469,((Glossina_fuscipes_tsetse_fly_Insecta_Glossinidae_XP_037901348.1:0.25659,
(Lucilia_cuprina_Australian_sheep_blowfly_Insecta_Calliphoridae_XP_023300428.1:0.02375
,Lucilia_sericata_common_green_bottle_fly_Insecta_Calliphoridae_XP_037813686.1:0.05303
)0.998:0.09888)0.894:0.04736,(Drosophila_busckii_flies_Insecta_Drosophilidae_XP_017840
508.2:0.20815,(Scaptodrosophila_lebanonensis_flies_Insecta_Drosophilidae_XP_030373294.
1:0.18875,((Drosophila_albomicans_flies_Insecta_Drosophilidae_XP_034103183.1:0.16956,(
Drosophila_grimshawi_flies_Insecta_Drosophilidae_XP_032599133.1:0.16045,((Drosophila_n
ovamexicana_flies_Insecta_Drosophilidae_XP_030567470.1:0.01179,Drosophila_virilis_flie
s_Insecta_Drosophilidae_XP_015030403.1:0.01226)1.000:0.09631,(Drosophila_hydei_flies_I
nsecta_Drosophilidae_XP_023160938.2:0.07809,Drosophila_navojoa_flies_Insecta_Drosophil
idae_XP_017955833.1:0.08809)1.000:0.09288)0.121:0.03635)0.906:0.02581)1.000:0.07655,(D
rosophila_willistoni_flies_Insecta_Drosophilidae_XP_015034555.1:0.11733,(((Drosophila_
ananassae_flies_Insecta_Drosophilidae_XP_014765084.1:0.04366,Drosophila_bipectinata_fl
ies_Insecta_Drosophilidae_XP_017090156.1:0.07188)1.000:0.07310,((Drosophila_serrata_fl
ies_Insecta_Drosophilidae_XP_020804588.1:0.08137,Drosophila_kikkawai_flies_Insecta_Dro
sophilidae_XP_017021104.1:0.05509)1.000:0.09524,((Drosophila_rhopaloa_flies_Insecta_Dr
osophilidae_XP_016973554.1:0.11202,Drosophila_elegans_flies_Insecta_Drosophilidae_XP_0
17112002.1:0.04119)0.981:0.02564,((Drosophila_eugracilis_flies_Insecta_Drosophilidae_X
P_017080934.1:0.07618,((((Drosophila_santomea_flies_Insecta_Drosophilidae_XP_039485252
.1:0.00472,Drosophila_yakuba_flies_Insecta_Drosophilidae_XP_015050240.1:0.03378)0.968:
0.01113,(Drosophila_melanogaster_fruit_fly_Insecta_Drosophilidae_NP_001261717.1:0.0098
1,(Drosophila_mauritiana_flies_Insecta_Drosophilidae_XP_033159688.1:0.00237,(Drosophil
a_sechellia_flies_Insecta_Drosophilidae_XP_032574143.1:0.00827,Drosophila_simulans_fli
es_Insecta_Drosophilidae_XP_016031451.1:0.00593)0.936:0.00467)0.994:0.01156)0.989:0.01
522)0.588:0.01356,Drosophila_erecta_flies_Insecta_Drosophilidae_XP_015013198.1:0.01163
)1.000:0.04835,Drosophila_ficusphila_flies_Insecta_Drosophilidae_XP_017060172.1:0.0876
4)0.902:0.01280)1.000:0.03678,(Drosophila_takahashii_flies_Insecta_Drosophilidae_XP_01
6992516.1:0.04042,(Drosophila_biarmipes_flies_Insecta_Drosophilidae_XP_016959135.1:0.0
3076,(Drosophila_suzukii_flies_Insecta_Drosophilidae_XP_016936128.2:0.00694,Drosophila

,(Ostrinia_furnacalis_Asian_corn_borer_Insecta_Crambidae_XP_028160456.1:0.29183,(Amyel
ois_transitella_moths_Insecta_Pyralidae_XP_013184189.1:0.26221,Galleria_mellonella_gre
ater_wax_moth_Insecta_Pyralidae_XP_031764408.1:0.35716)0.987:0.13076)0.816:0.06440)0.0
00:0.03288,(Papilio_xuthus_Asian_swallowtail_Insecta_Papilionidae_XP_013167983.1:0.460
58,Danaus_plexippus_plexippus_monarch_butterfly_Insecta_XP_032527598.1:0.34045)0.383:0
.05977)0.972:0.16332)0.917:0.13017)1.000:0.38661,((Drosophila_bipectinata_flies_Insect
a_Drosophilidae_XP_017087385.1:3.73907,((Anopheles_stephensi_Asian_malaria_mosquito_In
secta_Culicidae_XP_035913020.1:0.12458,(Anopheles_coluzzii_mosquitos_Insecta_Culicidae
_XP_040234579.1:0.00977,Anopheles_arabiensis_mosquitos_Insecta_Culicidae_XP_040168684.
1:0.00904)0.995:0.15965)1.000:0.56987,((((Leptinotarsa_decemlineata_Colorado_potato_be
etle_Insecta_Chrysomelidae_XP_023023611.1:0.20501,Leptinotarsa_decemlineata_Colorado_p
otato_beetle_Insecta_Chrysomelidae_XP_023023604.1:0.29384)0.930:0.09834,Diabrotica_vir
gifera_virgifera_western_corn_rootworm_Insecta_Chrysomelidae_XP_028151784.1:0.55814)0.
836:0.08742,(Sitophilus_oryzae_rice_weevil_Insecta_Curculionidae_XP_030759890.1:0.2790
7,Dendroctonus_ponderosae_mountain_pine_beetle_Insecta_Curculionidae_XP_019770659.1:0.
31963)0.982:0.11331)0.295:0.08299,Aethina_tumida_small_hive_beetle_Insecta_XP_01986772
1.1:0.36289)0.993:0.30604)0.525:0.33817)0.899:0.55922,((Nicrophorus_vespilloides_beetl
es_Insecta_Silphidae_XP_017781361.1:0.41350,(Cephus_cinctus_wheat_stem_sawfly_Insecta_
Cephidae_XP_015585197.1:0.24024,(Megachile_rotundata_alfalfa_leafcutting_bee_Insecta_M
egachilidae_XP_012150300.1:0.73085,Ceratosolen_solmsi_marchali_wasps_ants_and_bees_Ins
ecta_Agaonidae_XP_011501251.1:0.67922)1.000:0.47861)1.000:0.37127)0.537:0.06689,((Agri
lus_planipennis_emerald_ash_borer_Insecta_Buprestidae_XP_025836293.1:0.88905,((((Diabr
otica_virgifera_virgifera_western_corn_rootworm_Insecta_Chrysomelidae_XP_028149126.1:0
.48435,Leptinotarsa_decemlineata_Colorado_potato_beetle_Insecta_Chrysomelidae_XP_02301
8756.1:0.44757)0.077:0.09713,((((Aethina_tumida_small_hive_beetle_Insecta_XP_019872562
.1:0.26544,Aethina_tumida_small_hive_beetle_Insecta_XP_019872560.1:0.18833)1.000:0.288
28,(Anoplophora_glabripennis_Asian_longhorned_beetle_Insecta_Cerambycidae_XP_023310703
.1:0.32479,(Anoplophora_glabripennis_Asian_longhorned_beetle_Insecta_Cerambycidae_XP_0
18579941.1:0.24756,(Anoplophora_glabripennis_Asian_longhorned_beetle_Insecta_Cerambyci
dae_XP_018562726.1:0.05424,(Anoplophora_glabripennis_Asian_longhorned_beetle_Insecta_C
erambycidae_XP_018579928.1:0.02313,(Anoplophora_glabripennis_Asian_longhorned_beetle_I
nsecta_Cerambycidae_XP_018579931.1:0.01982,(Anoplophora_glabripennis_Asian_longhorned_
beetle_Insecta_Cerambycidae_XP_018579926.1:0.03108,Anoplophora_glabripennis_Asian_long
horned_beetle_Insecta_Cerambycidae_XP_023310739.1:0.03251)0.779:0.01979)0.999:0.04147)
0.849:0.01916)1.000:0.21294)1.000:0.25503)0.946:0.08368)0.680:0.02505,Tribolium_castan
eum_red_flour_beetle_Insecta_Tenebrionidae_XP_008192480.2:0.44934)0.295:0.04825,(Dendr
octonus_ponderosae_mountain_pine_beetle_Insecta_Curculionidae_XP_019760796.1:0.22796,D
endroctonus_ponderosae_mountain_pine_beetle_Insecta_Curculionidae_XP_019760795.1:0.150
59)1.000:0.40229)0.094:0.03413)0.950:0.07366,((Sitophilus_oryzae_rice_weevil_Insecta_C
urculionidae_XP_030760215.1:0.23025,(Sitophilus_oryzae_rice_weevil_Insecta_Curculionid
ae_XP_030760233.1:0.01520,Sitophilus_oryzae_rice_weevil_Insecta_Curculionidae_XP_03076
0214.1:0.01161)1.000:0.18805)1.000:0.38013,(Diabrotica_virgifera_virgifera_western_cor
n_rootworm_Insecta_Chrysomelidae_XP_028146538.1:0.08075,Diabrotica_virgifera_virgifera
_western_corn_rootworm_Insecta_Chrysomelidae_XP_028149125.1:0.11330)1.000:0.39097)0.69
5:0.06338)0.569:0.03526,Diabrotica_virgifera_virgifera_western_corn_rootworm_Insecta_C
hrysomelidae_XP_028151079.1:0.78914)0.955:0.12461)0.910:0.07895,(((Fopius_arisanus_was
ps_ants_and_bees_Insecta_Braconidae_XP_011312425.1:0.13701,Diachasma_alloeum_wasps_ant
s_and_bees_Insecta_Braconidae_XP_015109232.1:0.20710)1.000:0.33779,((Chelonus_insulari
s_wasps_ants_and_bees_Insecta_Braconidae_XP_034940913.1:0.28306,Microplitis_demolitor_
wasps_ants_and_bees_Insecta_Braconidae_XP_008558516.1:0.17531)0.962:0.08780,(Athalia_r
osae_coleseed_sawfly_Insecta_Tenthredinidae_XP_012268795.1:0.36850,(((((Megalopta_gen
alis_bees_Insecta_Halictidae_XP_033337109.1:0.22104,Nomia_melanderi_Alkali_bee_Insecta
_Halictidae_XP_031843675.1:0.26595)0.962:0.07342,Dufourea_novaeangliae_bees_Insecta_Ha
lictidae_XP_015431087.1:0.14946)0.993:0.10325,(Habropoda_laboriosa_bees_Insecta_Apidae
_XP_017793841.1:0.16256,(Eufriesea_mexicana_bees_Insecta_Apidae_XP_017762758.1:0.13826
,(Apis_florea_little_honeybee_Insecta_Apidae_XP_003694620.1:0.01703,(Apis_dorsata_gian
t_honeybee_Insecta_Apidae_XP_006619712.1:0.01325,Apis_mellifera_honey_bee_Insecta_Apid
ae_XP_394676.2:0.02653)0.952:0.01509)1.000:0.11190)0.992:0.05600)0.926:0.03683)0.926:0
.03050,(Megachile_rotundata_alfalfa_leafcutting_bee_Insecta_Megachilidae_XP_012149520.
1:0.10270,Osmia_bicornis_bicornis_red_mason_bee_Insecta_Megachilidae_XP_029051864.1:0.
10701)1.000:0.08784)1.000:0.16874,((Vespa_mandarinia_Asian_giant_hornet_Insecta_Vespid
ae_XP_035733253.1:0.10381,(Polistes_canadensis_wasps_ants_and_bees_Insecta_Vespidae_XP
_014603614.1:0.03857,Polistes_dominula_European_paper_wasp_Insecta_Vespidae_XP_0151916
23.1:0.04103)0.999:0.08609)1.000:0.15265,((Dinoponera_quadriceps_ants_Insecta_Formicid
ae_XP_014489230.1:0.13193,Odontomachus_brunneus_ants_Insecta_Formicidae_XP_032674810.1
:0.12452)0.967:0.05487,((Nylanderia_fulva_ants_Insecta_Formicidae_XP_029170832.1:0.240
73,(Formica_exsecta_ants_Insecta_Formicidae_XP_029679359.1:0.11351,Camponotus_floridan
us_Florida_carpenter_ant_Insecta_Formicidae_XP_011252996.1:0.11889)0.181:0.02549)0.987
:0.05903,(Pogonomyrmex_barbatus_red_harvester_ant_Insecta_Formicidae_XP_011638777.1:0.
15187,(Monomorium_pharaonis_pharaoh_ant_Insecta_Formicidae_XP_012530236.1:0.11945,((Tr
achymyrmex_septentrionalis_ants_Insecta_Formicidae_XP_018352761.1:0.07441,Acromyrmex_e
chinatior_Panamanian_leafcutter_ant_Insecta_Formicidae_XP_011055561.1:0.10758)1.000:0.
12371,(Wasmannia_auropunctata_little_fire_ant_Insecta_Formicidae_XP_011685682.1:0.1848

ura_flies_Insecta_Drosophilidae_XP_022225312.1:0.04254,(Drosophila_pseudoobscura_flies
_Insecta_Drosophilidae_XP_015042817.2:0.00506,(Drosophila_miranda_flies_Insecta_Drosop
hilidae_XP_017135378.1:0.00590,Drosophila_persimilis_flies_Insecta_Drosophilidae_XP_02
6845243.1:0.00353)0.863:0.00317)1.000:0.06020)0.948:0.04959)1.000:0.13612,((((Drosophi
la_yakuba_flies_Insecta_Drosophilidae_XP_002094593.2:0.00504,Drosophila_santomea_flies
_Insecta_Drosophilidae_XP_039486177.1:0.01502)0.959:0.01473,(Drosophila_erecta_flies_I
nsecta_Drosophilidae_XP_026832506.1:0.05212,(Drosophila_melanogaster_fruit_fly_Insecta
_Drosophilidae_NP_729808.1:0.02764,(Drosophila_mauritiana_flies_Insecta_Drosophilidae_
XP_033158344.1:0.00492,(Drosophila_sechellia_flies_Insecta_Drosophilidae_XP_032575275.
1:0.01490,Drosophila_simulans_flies_Insecta_Drosophilidae_XP_016031606.1:0.00747)0.296
:0.00138)0.962:0.01068)1.000:0.03615)0.248:0.00941)0.983:0.03296,((Drosophila_ficusphi
la_flies_Insecta_Drosophilidae_XP_017043461.1:0.06894,((Drosophila_rhopaloa_flies_Inse
cta_Drosophilidae_XP_016978086.1:0.05310,Drosophila_elegans_flies_Insecta_Drosophilida
e_XP_017128579.1:0.03447)0.972:0.02696,Drosophila_eugracilis_flies_Insecta_Drosophilid
ae_XP_017071623.1:0.08721)0.832:0.01460)0.763:0.01154,(Drosophila_takahashii_flies_Ins
ecta_Drosophilidae_XP_016994031.1:0.05504,(Drosophila_biarmipes_flies_Insecta_Drosophi
lidae_XP_016959216.1:0.04513,(Drosophila_suzukii_flies_Insecta_Drosophilidae_XP_036670
949.1:0.01599,Drosophila_subpulchrella_flies_Insecta_Drosophilidae_XP_037720337.1:0.01
828)0.657:0.00686)0.997:0.03472)0.960:0.02425)0.951:0.02134)0.925:0.02968,((Drosophila
_serrata_flies_Insecta_Drosophilidae_XP_020817113.1:0.06265,Drosophila_kikkawai_flies_
Insecta_Drosophilidae_XP_017016425.1:0.04019)1.000:0.07168,(Drosophila_ananassae_flies
_Insecta_Drosophilidae_XP_001957474.2:0.04690,Drosophila_bipectinata_flies_Insecta_Dro
sophilidae_XP_017100150.1:0.05571)1.000:0.07644)0.724:0.02471)0.997:0.09339)0.979:0.07
416)0.954:0.05863,Drosophila_willistoni_flies_Insecta_Drosophilidae_XP_015033360.1:0.1
5076)1.000:0.14093)0.976:0.10031)0.755:0.05530,Hermetia_illucens_flies_Insecta_Stratio
myidae_XP_037907536.1:0.50992)0.798:0.02545)0.911:0.07054,(((Hermetia_illucens_flies_I
nsecta_Stratiomyidae_XP_037908448.1:0.11817,Hermetia_illucens_flies_Insecta_Stratiomyi
dae_XP_037908449.1:0.11516)1.000:0.27417,((Hermetia_illucens_flies_Insecta_Stratiomyid
ae_XP_037907678.1:0.01152,Hermetia_illucens_flies_Insecta_Stratiomyidae_XP_037907676.1
:0.02704)1.000:0.37902,((Hermetia_illucens_flies_Insecta_Stratiomyidae_XP_037906521.1:
0.17857,(Hermetia_illucens_flies_Insecta_Stratiomyidae_XP_037907672.1:0.10648,Hermetia
_illucens_flies_Insecta_Stratiomyidae_XP_037907675.1:0.12038)0.580:0.06120)1.000:0.193
47,((Hermetia_illucens_flies_Insecta_Stratiomyidae_XP_037907679.1:0.10218,Hermetia_ill
ucens_flies_Insecta_Stratiomyidae_XP_037907680.1:0.15770)1.000:0.17032,(Hermetia_illuc
ens_flies_Insecta_Stratiomyidae_XP_037907677.1:0.21848,(Hermetia_illucens_flies_Insect
a_Stratiomyidae_XP_037907673.1:0.16709,Hermetia_illucens_flies_Insecta_Stratiomyidae_X
P_037907682.1:0.18711)0.860:0.04725)0.998:0.09272)0.840:0.04880)0.299:0.02899)0.996:0.
14038)0.990:0.12708,((Scaptodrosophila_lebanonensis_flies_Insecta_Drosophilidae_XP_030
386968.1:0.20681,(Drosophila_willistoni_flies_Insecta_Drosophilidae_XP_002066047.1:0.1
4917,((((Drosophila_subobscura_flies_Insecta_Drosophilidae_XP_034650957.1:0.00960,Dros
ophila_guanche_flies_Insecta_Drosophilidae_XP_034119871.1:0.01331)0.998:0.03666,(Droso
phila_obscura_flies_Insecta_Drosophilidae_XP_022229026.1:0.03655,(Drosophila_miranda_f
lies_Insecta_Drosophilidae_XP_017149075.1:0.00847,(Drosophila_pseudoobscura_flies_Inse
cta_Drosophilidae_XP_001360154.1:0.00064,Drosophila_persimilis_flies_Insecta_Drosophil
idae_XP_002015519.1:0.00238)0.837:0.00220)1.000:0.04336)0.575:0.01753)0.855:0.03520,((
Drosophila_ananassae_flies_Insecta_Drosophilidae_XP_001961288.1:0.03148,Drosophila_bip
ectinata_flies_Insecta_Drosophilidae_XP_017106724.1:0.03639)1.000:0.07654,((Drosophila
_serrata_flies_Insecta_Drosophilidae_XP_020815046.1:0.04188,Drosophila_kikkawai_flies_
Insecta_Drosophilidae_XP_017016528.1:0.02924)1.000:0.05815,(((((Drosophila_melanogaste
r_fruit_fly_Insecta_Drosophilidae_NP_610235.1:0.02547,(Drosophila_mauritiana_flies_Ins
ecta_Drosophilidae_XP_033155836.1:0.01121,(Drosophila_sechellia_flies_Insecta_Drosophi
lidae_XP_032571656.1:0.01148,Drosophila_simulans_flies_Insecta_Drosophilidae_XP_002080
329.1:0.00261)0.655:0.00272)0.960:0.00915)0.983:0.01586,(Drosophila_erecta_flies_Insec
ta_Drosophilidae_XP_001970728.1:0.02513,(Drosophila_yakuba_flies_Insecta_Drosophilidae
_XP_002089374.1:0.00793,Drosophila_santomea_flies_Insecta_Drosophilidae_XP_039479345.1
:0.00063)1.000:0.04458)0.946:0.01269)1.000:0.03577,((Drosophila_biarmipes_flies_Insect
a_Drosophilidae_XP_016958418.1:0.02822,(Drosophila_suzukii_flies_Insecta_Drosophilidae
_XP_016940456.1:0.03437,Drosophila_subpulchrella_flies_Insecta_Drosophilidae_XP_037717
232.1:0.03236)0.991:0.01761)0.977:0.01514,Drosophila_eugracilis_flies_Insecta_Drosophi
lidae_XP_017069220.1:0.10185)0.575:0.00887)0.805:0.00990,(Drosophila_ficusphila_flies_
Insecta_Drosophilidae_XP_017050277.1:0.05484,(Drosophila_rhopaloa_flies_Insecta_Drosop
hilidae_XP_016991591.1:0.04263,Drosophila_elegans_flies_Insecta_Drosophilidae_XP_01712
5823.1:0.02888)0.947:0.01136)0.411:0.00361)0.479:0.00526,Drosophila_takahashii_flies_I
nsecta_Drosophilidae_XP_017003853.1:0.03659)0.958:0.02792)0.975:0.03924)1.000:0.12063)
0.981:0.06668,(Drosophila_albomicans_flies_Insecta_Drosophilidae_XP_034110759.1:0.1611
0,((Drosophila_busckii_flies_Insecta_Drosophilidae_XP_017836296.1:0.15751,((Drosophila
_novamexicana_flies_Insecta_Drosophilidae_XP_030573913.1:0.00726,Drosophila_virilis_fl
ies_Insecta_Drosophilidae_XP_002050480.1:0.01472)1.000:0.07625,(Drosophila_hydei_flies
_Insecta_Drosophilidae_XP_023173859.1:0.08419,(Drosophila_navojoa_flies_Insecta_Drosop
hilidae_XP_017960108.1:0.03325,(Drosophila_mojavensis_flies_Insecta_Drosophilidae_XP_0
02004998.1:0.00529,Drosophila_arizonae_flies_Insecta_Drosophilidae_XP_017867367.1:0.01
429)0.953:0.01531)0.990:0.04069)0.999:0.07809)0.033:0.02498)0.810:0.03585,Drosophila_g
rimshawi_flies_Insecta_Drosophilidae_XP_001987140.1:0.19024)0.459:0.02585)0.984:0.0612
