# Supplementary material for: Inferences on the evolution of the ascorbic acid synthesis pathway in insects using Phylogenetic Tree Collapser (PTC), a tool for the automated collapsing of phylogenetic trees using taxonomic information
Source: J Integr Bioinform. 2024 Jul 24;21(2):20230051. doi: 10.1515/jib-2023-0051 (PMC11377030; doi:10.1515/jib-2023-0051)
Supplement: Supplementary file 1 — Supplementary Material Details [file j_jib-2023-0051_suppl_001.zip › Supplementary_File_5_DHR24.con_PDF.pdf]

```
#NEXUS

[ID: 6165140891]
begin taxa;
	dimensions ntax=93;
	taxlabels
		Diabrotica_virgifera_virgifera_western_corn_rootworm_Coleoptera_Chrysomelidae_XP_028128869.1
		Belonocnema_treatae_wasps_ants_and_bees_Hymenoptera_Cynipidae_XP_033219079.1
		Amyelois_transitella_moths_Lepidoptera_Pyralidae_XP_013196006.1
		Amyelois_transitella_moths_Lepidoptera_Pyralidae_XP_013193356.1
		Amyelois_transitella_moths_Lepidoptera_Pyralidae_XP_013193383.1
		Bicyclus_anynana_squinting_bush_brown_Lepidoptera_XP_023936591.1
		Bicyclus_anynana_squinting_bush_brown_Lepidoptera_XP_023935557.1
		Galleria_mellonella_greater_wax_moth_Lepidoptera_Pyralidae_XP_026755859.1
		Galleria_mellonella_greater_wax_moth_Lepidoptera_Pyralidae_XP_026760256.1
		Sitophilus_oryzae_rice_weevil_Coleoptera_Curculionidae_XP_030746318.1
		Manduca_sexta_tobacco_hornworm_Lepidoptera_Sphingidae_XP_037295187.1
		Manduca_sexta_tobacco_hornworm_Lepidoptera_Sphingidae_XP_030025268.2
		Dendroctonus_ponderosae_mountain_pine_beetle_Coleoptera_Curculionidae_XP_019754799.1
		Papilio_xuthus_Asian_swallowtail_Lepidoptera_Papilionidae_XP_013163008.1
		Papilio_xuthus_Asian_swallowtail_Lepidoptera_Papilionidae_XP_013174719.1
		Contarinia_nasturtii_swede_midge_Diptera_Cecidomyiidae_XP_031630419.1
		Coccinella_septempunctata_seven_spotted_ladybird_Coleoptera_Coccinellidae_XP_044761273.1
		Ostrinia_furnacalis_Asian_corn_borer_Lepidoptera_Crambidae_XP_028161388.1
		Ostrinia_furnacalis_Asian_corn_borer_Lepidoptera_Crambidae_XP_028159707.1
		Spodoptera_frugiperda_fall_armyworm_Lepidoptera_XP_035434723.1
		Spodoptera_frugiperda_fall_armyworm_Lepidoptera_XP_035434722.1
		Spodoptera_frugiperda_fall_armyworm_Lepidoptera_XP_035456981.1
		Pieris_rapae_cabbage_white_Lepidoptera_Pieridae_XP_022118425.1
		Pieris_rapae_cabbage_white_Lepidoptera_Pieridae_XP_022118448.1
		Vanessa_tameamea_butterflies_Lepidoptera_XP_026492295.1
		Vanessa_tameamea_butterflies_Lepidoptera_XP_026486819.1
		Aphis_gossypii_cotton_aphid_Hemiptera_Aphididae_XP_027848709.1
		Aphis_gossypii_cotton_aphid_Hemiptera_Aphididae_XP_027838422.1
		Acyrthosiphon_pisum_pea_aphid_Hemiptera_Aphididae_XP_001951836.2
		Zerene_cesonia_dogface_butterfly_Lepidoptera_Pieridae_XP_038211373.1
		Zerene_cesonia_dogface_butterfly_Lepidoptera_Pieridae_XP_038219324.1
		Folsomia_candida_springtails_Entomobryomorpha_Isotomidae_XP_021952028.1
		Folsomia_candida_springtails_Entomobryomorpha_Isotomidae_XP_021959027.1
		Folsomia_candida_springtails_Entomobryomorpha_Isotomidae_XP_021953606.2
		Folsomia_candida_springtails_Entomobryomorpha_Isotomidae_XP_021960340.2
		Folsomia_candida_springtails_Entomobryomorpha_Isotomidae_XP_021966010.2
		Folsomia_candida_springtails_Entomobryomorpha_Isotomidae_XP_035716487.1
		Folsomia_candida_springtails_Entomobryomorpha_Isotomidae_XP_021966080.2
		Folsomia_candida_springtails_Entomobryomorpha_Isotomidae_XP_035700411.1
		Folsomia_candida_springtails_Entomobryomorpha_Isotomidae_XP_021962205.1
		Folsomia_candida_springtails_Entomobryomorpha_Isotomidae_XP_021962012.1
		Folsomia_candida_springtails_Entomobryomorpha_Isotomidae_XP_021950005.1
		Folsomia_candida_springtails_Entomobryomorpha_Isotomidae_XP_021960122.2
		Agrilus_planipennis_emerald_ash_borer_Coleoptera_Buprestidae_XP_018321456.1
		Agrilus_planipennis_emerald_ash_borer_Coleoptera_Buprestidae_XP_018326951.2
		Anoplophora_glabripennis_Asian_longhorned_beetle_Coleoptera_Cerambycidae_XP_018569440.1
		Hyposmocoma_kahamanoa_moths_Lepidoptera_Cosmopterigidae_XP_026320656.1
		Hyposmocoma_kahamanoa_moths_Lepidoptera_Cosmopterigidae_XP_026317233.1
		Trichoplusia_ni_cabbage_looper_Lepidoptera_XP_026739142.1
		Trichoplusia_ni_cabbage_looper_Lepidoptera_XP_026733822.1
		Nilaparvata_lugens_brown_planthopper_Hemiptera_Delphacidae_XP_039279748.1
		Melanaphis_sacchari_aphids_Hemiptera_Aphididae_XP_025206571.1
		Melanaphis_sacchari_aphids_Hemiptera_Aphididae_XP_025207550.1
		Helicoverpa_armigera_cotton_bollworm_Lepidoptera_XP_021184790.1
		Culex_pipiens_pallens_northern_house_mosquito_Diptera_Culicidae_XP_039444131.1
		Mus_musculus_house_mouse_Rodentia_Muridae_NP_444502.2
		Aricia_agestis_brown_argus_Lepidoptera_Lycaenidae_XP_041984337.1
```

```
		Onthophagus_taurus_beetles_Coleoptera_Scarabaeidae_XP_022905729.1
		Onthophagus_taurus_beetles_Coleoptera_Scarabaeidae_XP_022915592.1
		Leptopilina_heterotoma_wasps_ants_and_bees_Hymenoptera_Figitidae_XP_043467652.1
		Thrips_palmi_thrips_Thysanoptera_Thripidae_XP_034245473.1
		Thrips_palmi_thrips_Thysanoptera_Thripidae_XP_034251850.1
		Myzus_persicae_green_peach_aphid_Hemiptera_Aphididae_XP_022165326.1
		Myzus_persicae_green_peach_aphid_Hemiptera_Aphididae_XP_022169098.1
		Tribolium_madens_black_flour_beetle_Coleoptera_Tenebrionidae_XP_044263767.1
		Tribolium_madens_black_flour_beetle_Coleoptera_Tenebrionidae_XP_044256861.1
		Bombyx_mori_domestic_silkworm_Lepidoptera_Bombycidae_XP_004926866.1
		Bombyx_mori_domestic_silkworm_Lepidoptera_Bombycidae_XP_004926210.1
		Bemisia_tabaci_sweet_potato_whitefly_Hemiptera_Aleyrodidae_XP_018912280.1
		Nasonia_vitripennis_jewel_wasp_Hymenoptera_Pteromalidae_XP_001607022.2
		Bradysia_coprophila_flies_Diptera_Sciaridae_XP_037044853.1
		Bradysia_coprophila_flies_Diptera_Sciaridae_XP_037049352.1
		Cryptotermes_secundus_termites_Blattodea_Kalotermitidae_XP_023722549.1
		Cephus_cinctus_wheat_stem_sawfly_Hymenoptera_Cephidae_XP_024946735.1
		Zootermopsis_nevadensis_termites_Blattodea_Termopsidae_XP_021915139.1
		Papilio_machaon_common_yellow_swallowtail_Lepidoptera_Papilionidae_XP_014368567.1
		Sipha_flava_yellow_sugarcane_aphid_Hemiptera_Aphididae_XP_025418787.1
		Homo_sapiens_human_Primates_Hominidae_NP_055577.1
		Tribolium_castaneum_red_flour_beetle_Coleoptera_Tenebrionidae_XP_966520.1
		Tribolium_castaneum_red_flour_beetle_Coleoptera_Tenebrionidae_XP_001810773.1
		Ceratosolen_solmsi_marchali_wasps_ants_and_bees_Hymenoptera_Agaonidae_XP_011499083.1
		Danaus_plexippus_plexippus_monarch_butterfly_Lepidoptera_XP_032519817.1
		Danaus_plexippus_plexippus_monarch_butterfly_Lepidoptera_XP_032524720.1
		Aedes_aegypti_yellow_fever_mosquito_Diptera_Culicidae_XP_001655874.2
		Papilio_polytes_common_Mormon_Lepidoptera_Papilionidae_XP_013137479.1
		Papilio_polytes_common_Mormon_Lepidoptera_Papilionidae_XP_013148851.1
		Bombyx_mandarina_wild_silkworm_Lepidoptera_Bombycidae_XP_028038301.1
		Bombyx_mandarina_wild_silkworm_Lepidoptera_Bombycidae_XP_028025733.1
		Diaphorina_citri_Asian_citrus_psyllid_Hemiptera_Liviidae_XP_026686359.1
		Aedes_albopictus_Asian_tiger_mosquito_Diptera_Culicidae_XP_019563273.2
		Rhopalosiphum_maidis_corn_leaf_aphid_Hemiptera_Aphididae_XP_026818618.1
		Rhopalosiphum_maidis_corn_leaf_aphid_Hemiptera_Aphididae_XP_026812544.1
		Athalia_rosae_coleseed_sawfly_Hymenoptera_Tenthredinidae_XP_012252433.1
		;
end;
begin trees;
	translate
		1	Diabrotica_virgifera_virgifera_western_corn_rootworm_Coleoptera_Chrysomelidae_XP_028128869.1,
		2	Belonocnema_treatae_wasps_ants_and_bees_Hymenoptera_Cynipidae_XP_033219079.1,
		3	Amyelois_transitella_moths_Lepidoptera_Pyralidae_XP_013196006.1,
		4	Amyelois_transitella_moths_Lepidoptera_Pyralidae_XP_013193356.1,
		5	Amyelois_transitella_moths_Lepidoptera_Pyralidae_XP_013193383.1,
		6	Bicyclus_anynana_squinting_bush_brown_Lepidoptera_XP_023936591.1,
		7	Bicyclus_anynana_squinting_bush_brown_Lepidoptera_XP_023935557.1,
		8	Galleria_mellonella_greater_wax_moth_Lepidoptera_Pyralidae_XP_026755859.1,
		9	Galleria_mellonella_greater_wax_moth_Lepidoptera_Pyralidae_XP_026760256.1,
		10	Sitophilus_oryzae_rice_weevil_Coleoptera_Curculionidae_XP_030746318.1,
		11	Manduca_sexta_tobacco_hornworm_Lepidoptera_Sphingidae_XP_037295187.1,
		12	Manduca_sexta_tobacco_hornworm_Lepidoptera_Sphingidae_XP_030025268.2,
		13	Dendroctonus_ponderosae_mountain_pine_beetle_Coleoptera_Curculionidae_XP_019754799.1,
		14	Papilio_xuthus_Asian_swallowtail_Lepidoptera_Papilionidae_XP_013163008.1,
		15	Papilio_xuthus_Asian_swallowtail_Lepidoptera_Papilionidae_XP_013174719.1,
		16	Contarinia_nasturtii_swede_midge_Diptera_Cecidomyiidae_XP_031630419.1,
		17	
```

```
Coccinella_septempunctata_seven_spotted_ladybird_Coleoptera_Coccinellidae_XP_044761273.1,
		18	Ostrinia_furnacalis_Asian_corn_borer_Lepidoptera_Crambidae_XP_028161388.1,
		19	Ostrinia_furnacalis_Asian_corn_borer_Lepidoptera_Crambidae_XP_028159707.1,
		20	Spodoptera_frugiperda_fall_armyworm_Lepidoptera_XP_035434723.1,
		21	Spodoptera_frugiperda_fall_armyworm_Lepidoptera_XP_035434722.1,
		22	Spodoptera_frugiperda_fall_armyworm_Lepidoptera_XP_035456981.1,
		23	Pieris_rapae_cabbage_white_Lepidoptera_Pieridae_XP_022118425.1,
		24	Pieris_rapae_cabbage_white_Lepidoptera_Pieridae_XP_022118448.1,
		25	Vanessa_tameamea_butterflies_Lepidoptera_XP_026492295.1,
		26	Vanessa_tameamea_butterflies_Lepidoptera_XP_026486819.1,
		27	Aphis_gossypii_cotton_aphid_Hemiptera_Aphididae_XP_027848709.1,
		28	Aphis_gossypii_cotton_aphid_Hemiptera_Aphididae_XP_027838422.1,
		29	Acyrthosiphon_pisum_pea_aphid_Hemiptera_Aphididae_XP_001951836.2,
		30	Zerene_cesonia_dogface_butterfly_Lepidoptera_Pieridae_XP_038211373.1,
		31	Zerene_cesonia_dogface_butterfly_Lepidoptera_Pieridae_XP_038219324.1,
		32	Folsomia_candida_springtails_Entomobryomorpha_Isotomidae_XP_021952028.1,
		33	Folsomia_candida_springtails_Entomobryomorpha_Isotomidae_XP_021959027.1,
		34	Folsomia_candida_springtails_Entomobryomorpha_Isotomidae_XP_021953606.2,
		35	Folsomia_candida_springtails_Entomobryomorpha_Isotomidae_XP_021960340.2,
		36	Folsomia_candida_springtails_Entomobryomorpha_Isotomidae_XP_021966010.2,
		37	Folsomia_candida_springtails_Entomobryomorpha_Isotomidae_XP_035716487.1,
		38	Folsomia_candida_springtails_Entomobryomorpha_Isotomidae_XP_021966080.2,
		39	Folsomia_candida_springtails_Entomobryomorpha_Isotomidae_XP_035700411.1,
		40	Folsomia_candida_springtails_Entomobryomorpha_Isotomidae_XP_021962205.1,
		41	Folsomia_candida_springtails_Entomobryomorpha_Isotomidae_XP_021962012.1,
		42	Folsomia_candida_springtails_Entomobryomorpha_Isotomidae_XP_021950005.1,
		43	Folsomia_candida_springtails_Entomobryomorpha_Isotomidae_XP_021960122.2,
		44	Agrilus_planipennis_emerald_ash_borer_Coleoptera_Buprestidae_XP_018321456.1,
		45	Agrilus_planipennis_emerald_ash_borer_Coleoptera_Buprestidae_XP_018326951.2,
		46	Anoplophora_glabripennis_Asian_longhorned_beetle_Coleoptera_Cerambycidae_XP_018569440.1,
		47	Hyposmocoma_kahamanoa_moths_Lepidoptera_Cosmopterigidae_XP_026320656.1,
		48	Hyposmocoma_kahamanoa_moths_Lepidoptera_Cosmopterigidae_XP_026317233.1,
		49	Trichoplusia_ni_cabbage_looper_Lepidoptera_XP_026739142.1,
		50	Trichoplusia_ni_cabbage_looper_Lepidoptera_XP_026733822.1,
		51	Nilaparvata_lugens_brown_planthopper_Hemiptera_Delphacidae_XP_039279748.1,
		52	Melanaphis_sacchari_aphids_Hemiptera_Aphididae_XP_025206571.1,
		53	Melanaphis_sacchari_aphids_Hemiptera_Aphididae_XP_025207550.1,
		54	Helicoverpa_armigera_cotton_bollworm_Lepidoptera_XP_021184790.1,
		55	Culex_pipiens_pallens_northern_house_mosquito_Diptera_Culicidae_XP_039444131.1,
		56	Mus_musculus_house_mouse_Rodentia_Muridae_NP_444502.2,
		57	Aricia_agestis_brown_argus_Lepidoptera_Lycaenidae_XP_041984337.1,
		58	Onthophagus_taurus_beetles_Coleoptera_Scarabaeidae_XP_022905729.1,
		59	Onthophagus_taurus_beetles_Coleoptera_Scarabaeidae_XP_022915592.1,
		60	Leptopilina_heterotoma_wasps_ants_and_bees_Hymenoptera_Figitidae_XP_043467652.1,
		61	Thrips_palmi_thrips_Thysanoptera_Thripidae_XP_034245473.1,
		62	Thrips_palmi_thrips_Thysanoptera_Thripidae_XP_034251850.1,
		63	Myzus_persicae_green_peach_aphid_Hemiptera_Aphididae_XP_022165326.1,
		64	Myzus_persicae_green_peach_aphid_Hemiptera_Aphididae_XP_022169098.1,
		65	Tribolium_madens_black_flour_beetle_Coleoptera_Tenebrionidae_XP_044263767.1,
		66	Tribolium_madens_black_flour_beetle_Coleoptera_Tenebrionidae_XP_044256861.1,
		67	Bombyx_mori_domestic_silkworm_Lepidoptera_Bombycidae_XP_004926866.1,
		68	Bombyx_mori_domestic_silkworm_Lepidoptera_Bombycidae_XP_004926210.1,
		69	Bemisia_tabaci_sweet_potato_whitefly_Hemiptera_Aleyrodidae_XP_018912280.1,
		70	Nasonia_vitripennis_jewel_wasp_Hymenoptera_Pteromalidae_XP_001607022.2,
		71	Bradysia_coprophila_flies_Diptera_Sciaridae_XP_037044853.1,
		72	Bradysia_coprophila_flies_Diptera_Sciaridae_XP_037049352.1,
		73	Cryptotermes_secundus_termites_Blattodea_Kalotermitidae_XP_023722549.1,
		74	Cephus_cinctus_wheat_stem_sawfly_Hymenoptera_Cephidae_XP_024946735.1,
```

```
		75	Zootermopsis_nevadensis_termites_Blattodea_Termopsidae_XP_021915139.1,
		76	Papilio_machaon_common_yellow_swallowtail_Lepidoptera_Papilionidae_XP_014368567.1,
		77	Sipha_flava_yellow_sugarcane_aphid_Hemiptera_Aphididae_XP_025418787.1,
		78	Homo_sapiens_human_Primates_Hominidae_NP_055577.1,
		79	Tribolium_castaneum_red_flour_beetle_Coleoptera_Tenebrionidae_XP_966520.1,
		80	Tribolium_castaneum_red_flour_beetle_Coleoptera_Tenebrionidae_XP_001810773.1,
		81	Ceratosolen_solmsi_marchali_wasps_ants_and_bees_Hymenoptera_Agaonidae_XP_011499083.1,
		82	Danaus_plexippus_plexippus_monarch_butterfly_Lepidoptera_XP_032519817.1,
		83	Danaus_plexippus_plexippus_monarch_butterfly_Lepidoptera_XP_032524720.1,
		84	Aedes_aegypti_yellow_fever_mosquito_Diptera_Culicidae_XP_001655874.2,
		85	Papilio_polytes_common_Mormon_Lepidoptera_Papilionidae_XP_013137479.1,
		86	Papilio_polytes_common_Mormon_Lepidoptera_Papilionidae_XP_013148851.1,
		87	Bombyx_mandarina_wild_silkworm_Lepidoptera_Bombycidae_XP_028038301.1,
		88	Bombyx_mandarina_wild_silkworm_Lepidoptera_Bombycidae_XP_028025733.1,
		89	Diaphorina_citri_Asian_citrus_psyllid_Hemiptera_Liviidae_XP_026686359.1,
		90	Aedes_albopictus_Asian_tiger_mosquito_Diptera_Culicidae_XP_019563273.2,
		91	Rhopalosiphum_maidis_corn_leaf_aphid_Hemiptera_Aphididae_XP_026818618.1,
		92	Rhopalosiphum_maidis_corn_leaf_aphid_Hemiptera_Aphididae_XP_026812544.1,
		93	Athalia_rosae_coleseed_sawfly_Hymenoptera_Tenthredinidae_XP_012252433.1
		;
   [Note: This tree contains information on the topology, 
          branch lengths (if present), and the probability
          of the partition indicated by the branch.]
   tree con_50_majrule = (1:0.4820624,((((((((2:0.1952471,60:0.2572391)1.000:0.1464791,(74:0.2864884,93:0.3894537)1.000:0.08685935)1.000:0.1296868,(70:0.3253865,81:0.3183798)1.000:0.3187864)1.000:0.1505213,33:0.8176387)0.956:0.08209509,((((((3:0.2509037,((((((6:0.1956423,25:0.2383883)1.000:0.06029972,(23:0.2896059,30:0.2059861)1.000:0.07295506)0.999:0.03913275,(57:0.2628515,82:0.2905419)0.999:0.05216194)1.000:0.05924736,8:0.2534227)0.749:0.02519623,(((11:0.2126263,(68:0.003951581,87:0.01075097)1.000:0.322658)0.955:0.05041839,((20:0.02313419,21:0.0124026)1.000:0.1554686,49:0.136907)1.000:0.06882259)0.932:0.03766579,(((14:0.05540712,76:0.04953504)1.000:0.05421477,85:0.05832763)1.000:0.1823831,18:0.2188281)0.944:0.05272852)0.885:0.02808654)0.829:0.03720269,47:0.3529047)0.936:0.05507305)0.998:0.1974777,61:0.5628573)0.945:0.1186073,((44:0.3945495,(65:0.1098838,79:0.1393594)1.000:0.258369)1.000:0.09686927,58:0.4765958)0.999:0.1022995)0.859:0.1582556,71:0.5177863)0.560:0.1164642,32:0.364209)1.000:1.218382,(((34:1.129376,((40:0.02780484,41:0.02632553)1.000:0.0722729,42:0.1190655)1.000:0.9582606)0.532:0.1441894,(35:0.353296,43:0.3864925)1.000:0.6003656)1.000:0.3709534,(((36:0.1146787,39:0.09980908)1.000:0.1259205,37:0.3034682)1.000:0.1174723,38:0.2905167)1.000:0.7433809)1.000:0.2548757)1.000:0.182569,((((((27:0.06165485,91:0.0593845)0.747:0.007841746,53:0.0700827)0.999:0.03102219,63:0.08093416)1.000:0.1421369,((((28:0.06379449,52:0.07552833)0.880:0.006891083,92:0.05690525)1.000:0.02925863,(29:0.05101238,64:0.05512306)1.000:0.03752828)1.000:0.1107493,77:0.2067445)0.996:0.09476037)1.000:0.823697,89:1.943209)0.816:0.1386297,(51:0.6432864,69:0.6747181)0.760:0.0960275)0.997:0.07155272)0.994:0.06631156)0.695:0.05244324,(((((4:0.1308213,5:0.1990061)1.000:0.118889,((((7:0.2587519,((24:0.3120771,31:0.2770049)1.000:0.1329302,(26:0.2072648,83:0.2632766)0.996:0.05753775)1.000:0.06476827)1.000:0.09281864,((12:0.22813,(67:0.00360072,88:0.009247078)1.000:0.2809756)1.000:0.08516706,((22:0.1186183,54:0.1531356)1.000:0.06236373,50:0.2019055)1.000:0.06859834)0.674:0.02888355,(15:0.1099585,86:0.09584099)1.000:0.280443,48:0.3009304)0.681:0.02982663,19:0.1660482)0.680:0.03922459,9:0.2927167)1.000:0.06584945)1.000:0.3804657,62:0.4358135)0.625:0.0766566,(56:0.07314227,78:0.03902846)1.000:0.5532554)0.995:0.1527249,((16:0.5246387,72:0.4397108)0.818:0.06709955,(55:0.1652702,(84:0.09464424,90:0.09912371)1.000:0.08232629)1.000:0.2881698)1.000:0.2544013,(73:0.1367995,75:0.07698882)1.000:0.4672741)0.663:0.07611425)1.000:0.1200143,(45:0.5077817,59:0.4181331)1.000:0.1374023)0.990:0.06986706,(17:0.5631971,(66:0.09473933,80:0.06559632)1.000:0.386854)0.891:0.07401242)0.999:0.06541536,((10:0.2972265,13:0.3770667)0.998:0.0738356,46:0.4403061)0.801:0.0448079);

   [Note: This tree contains information only on the topology
          and branch lengths (median of the posterior probability density).]
   tree con_50_majrule = (1:0.4820624,((((((((2:0.1952471,60:0.2572391):0.1464791,(74:0.2864884,93:0.3894537):0.08685935):0.1296868,(70:0.3253865,81:0.3183798):0.3187864):0.1505213,33:0.8176387):0.08209509,((((((3:0.2509037,((((((6:0.1956423,25:0.2383883):0.06029972,(23:0.2896059,30:0.2059861):0.07295506):0.03913275,(57:0.2628515,82:0.2905419):0.05216194
```

```
):0.05924736,8:0.2534227):0.02519623,(((11:0.2126263,(68:0.003951581,87:0.01075097)
:0.322658):0.05041839,((20:0.02313419,21:0.0124026):0.1554686,49:0.136907):0.068822
59):0.03766579,(((14:0.05540712,76:0.04953504):0.05421477,85:0.05832763):0.1823831,
18:0.2188281):0.05272852):0.02808654):0.03720269,47:0.3529047):0.05507305):0.197477
7,61:0.5628573):0.1186073,((44:0.3945495,(65:0.1098838,79:0.1393594):0.258369):0.09
686927,58:0.4765958):0.1022995):0.1582556,71:0.5177863):0.1164642,32:0.364209):1.21
8382,(((34:1.129376,((40:0.02780484,41:0.02632553):0.0722729,42:0.1190655):0.958260
6):0.1441894,(35:0.353296,43:0.3864925):0.6003656):0.3709534,(((36:0.1146787,39:0.0
9980908):0.1259205,37:0.3034682):0.1174723,38:0.2905167):0.7433809):0.2548757):0.18
2569,((((((27:0.06165485,91:0.0593845):0.007841746,53:0.0700827):0.03102219,63:0.08
093416):0.1421369,((((28:0.06379449,52:0.07552833):0.006891083,92:0.05690525):0.029
25863,(29:0.05101238,64:0.05512306):0.03752828):0.1107493,77:0.2067445):0.09476037)
:0.823697,89:1.943209):0.1386297,(51:0.6432864,69:0.6747181):0.0960275):0.07155272)
:0.06631156):0.05244324,(((((4:0.1308213,5:0.1990061):0.118889,((((7:0.2587519,((24
:0.3120771,31:0.2770049):0.1329302,(26:0.2072648,83:0.2632766):0.05753775):0.064768
27):0.09281864,((12:0.22813,(67:0.00360072,88:0.009247078):0.2809756):0.08516706,((
22:0.1186183,54:0.1531356):0.06236373,50:0.2019055):0.06859834):0.02888355,(15:0.10
99585,86:0.09584099):0.280443,48:0.3009304):0.02982663,19:0.1660482):0.03922459,9:0
.2927167):0.06584945):0.3804657,62:0.4358135):0.0766566,(56:0.07314227,78:0.0390284
6):0.5532554):0.1527249,((16:0.5246387,72:0.4397108):0.06709955,(55:0.1652702,(84:0
.09464424,90:0.09912371):0.08232629):0.2881698):0.2544013,(73:0.1367995,75:0.076988
82):0.4672741):0.07611425):0.1200143,(45:0.5077817,59:0.4181331):0.1374023):0.06986
706,(17:0.5631971,(66:0.09473933,80:0.06559632):0.386854):0.07401242):0.06541536,((
10:0.2972265,13:0.3770667):0.0738356,46:0.4403061):0.0448079);LF
end;LF
```
