# Supplementary material for: Inferences on the evolution of the ascorbic acid synthesis pathway in insects using Phylogenetic Tree Collapser (PTC), a tool for the automated collapsing of phylogenetic trees using taxonomic information
Source: J Integr Bioinform. 2024 Jul 24;21(2):20230051. doi: 10.1515/jib-2023-0051 (PMC11377030; doi:10.1515/jib-2023-0051)
Supplement: Supplementary file 1 — Supplementary Material Details [file j_jib-2023-0051_suppl_001.zip › Supplementary_File_6_PGM1.con_PDF.pdf]

```
#NEXUS

[ID: 6929825472]
begin taxa;
	dimensions ntax=157;
	taxlabels
		Homo_sapiens_human_Mammalia_Hominidae_NP_002624.2
		Stomoxys_calcitrans_stable_fly_Insecta_Muscidae_XP_013103995.1
		Nomia_melanderi_Alkali_bee_Insecta_Halictidae_XP_031842685.1
		Folsomia_candida_springtails_Collembola_Isotomidae_XP_021955268.1
		Harpegnathos_saltator_Jerdons_jumping_ant_Insecta_Formicidae_XP_011140815.1
		Apis_dorsata_giant_honeybee_Insecta_Apidae_XP_006609201.1
		Linepithema_humile_Argentine_ant_Insecta_Formicidae_XP_012224343.1
		Acromyrmex_echinatior_Panamanian_leafcutter_ant_Insecta_Formicidae_XP_011055210.1
		Mus_musculus_house_mouse_Mammalia_Muridae_NP_082408.3
		Sitophilus_oryzae_Insecta_Curculionidae_XP_030749057.1
		Nylanderia_fulva_ants_Insecta_Formicidae_XP_029173334.1
		Pseudomyrmex_gracilis_ants_Insecta_Formicidae_XP_020289773.1
		Pediculus_humanus_corporis_human_body_louse_Insecta_Pediculidae_XP_002424860.1
		Pogonomyrmex_barbatus_red_harvester_ant_Insecta_Formicidae_XP_011641679.1
		Monomorium_pharaonis_pharaoh_ant_Insecta_Formicidae_XP_012536222.1
		Polistes_canadensis_wasps_ants_and_bees_Insecta_Vespidae_XP_014610425.1
		Camponotus_floridanus_Florida_carpenter_ant_Insecta_Formicidae_XP_011254716.1
		Zootermopsis_nevadensis_termites_Insecta_Termopsidae_XP_021927166.1
		Papilio_xuthus_Asian_swallowtail_Insecta_Papilionidae_XP_013166807.1
		Dendroctonus_ponderosae_Insecta_Curculionidae_XP_019759060.1
		Vollenhovia_emeryi_ants_Insecta_Formicidae_XP_011864933.1
		Vespa_mandarinia_Asian_giant_hornet_Insecta_Vespidae_XP_035737949.1
		Polistes_dominula_European_paper_wasp_Insecta_Vespidae_XP_015187969.1
		Microplitis_demolitor_wasps_ants_and_bees_Insecta_Braconidae_XP_014296476.1
		Dinoponera_quadriceps_ants_Insecta_Formicidae_XP_014488664.1
		Trachymyrmex_zeteki_ants_Insecta_Formicidae_XP_018308784.1
		Cyphomyrmex_costatus_ants_Insecta_Formicidae_XP_018405593.1
		Trachymyrmex_cornetzi_ants_Insecta_Formicidae_XP_018364082.1
		Temnothorax_curvispinosus_ants_Insecta_Formicidae_XP_024879566.1
		Wasmannia_auropunctata_little_fire_ant_Insecta_Formicidae_XP_011693239.1
		Trachymyrmex_septentrionalis_ants_Insecta_Formicidae_XP_018344009.1
		Atta_colombica_ants_Insecta_Formicidae_XP_018044394.1
		Athalia_rosae_Insecta_Tenthredinidae_XP_012267628.1
		Papilio_machaon_common_yellow_swallowtail_Insecta_Papilionidae_XP_014361343.1
		Anoplophora_glabripennis_Asian_longhorned_beetle_Insecta_Cerambycidae_XP_023309822.1
		Dufourea_novaeangliae_Insecta_Halictidae_XP_015433181.1
		Ooceraea_biroi_clonal_raider_ant_Insecta_Formicidae_XP_011340302.2
		Habropoda_laboriosa_bees_Insecta_Apidae_XP_017794782.1
		Ceratina_calcarata_bees_Insecta_Apidae_XP_026673966.1
		Osmia_bicornis_bicornis_red_mason_bee_Insecta_Megachilidae_XP_029036921.1
		Osmia_lignaria_orchard_mason_bee_Insecta_Megachilidae_XP_034193789.1
		Cephus_cinctus_wheat_stem_sawfly_Insecta_Cephidae_XP_015597724.1
		Apis_florea_little_honeybee_Insecta_Apidae_XP_003695955.1
		Apis_cerana_Asiatic_honeybee_Insecta_Apidae_XP_016912531.1
		Eufriesea_mexicana_bees_Insecta_Apidae_XP_017753798.1
		Bombus_terrestris_bufftailed_bumblebee_Insecta_Apidae_XP_003401536.1
		Bombus_bifarius_bees_Insecta_Apidae_XP_033300165.1
		Bombus_vosnesenskii_bees_Insecta_Apidae_XP_033344194.1
		Diabrotica_virgifera_virgifera_western_corn_rootworm_Insecta_Chrysomelidae_XP_028143837.1
		Apis_mellifera_honey_bee_Insecta_Apidae_XP_395366.2
		Belonocnema_treatae_Insecta_Cynipidae_XP_033214709.1
		Ceratosolen_solmsi_marchali_wasps_ants_and_bees_Insecta_Agaonidae_XP_011498014.1
		Copidosoma_floridanum_wasps_ants_and_bees_Insecta_Encyrtidae_XP_014206533.1
```

60 Trichogramma_pretiosum_wasps_ants_and_bees_Insecta_Trichogrammatidae_XP_014236333.1
61 Nasonia_vitripennis_jewel_wasp_Insecta_Pteromalidae_XP_001608147.1
62 Odontomachus_brunneus_ants_Insecta_Formicidae_XP_032684609.1
63 Drosophila_grimshawi_flies_Insecta_Drosophilidae_XP_001984409.1
64 Megachile_rotundata_alfalfa_leafcutting_bee_Insecta_Megachilidae_XP_003699636.1
65 Cryptotermes_secundus_termites_Insecta_Kalotermitidae_XP_023719654.1
66 Bemisia_tabaci_sweet_potato_whitefly_Insecta_Aleyrodidae_XP_018903307.1
67 Photinus_pyralis_common_eastern_firefly_Insecta_Lampyridae_XP_031330233.1
68 Drosophila_busckii_flies_Insecta_Drosophilidae_XP_017844450.1
69 Nicrophorus_vespilloides_beetles_Insecta_Silphidae_XP_017786364.1
70 Aedes_albopictus_Asian_tiger_mosquito_Insecta_Culicidae_XP_019543107.2
71 Nilaparvata_lugens_brown_planthopper_Insecta_Delphacidae_XP_039286781.1
72 Culex_quinquefasciatus_southern_house_mosquito_Insecta_Culicidae_XP_001847230.1
73 Culex_pipiens_pallens_northern_house_mosquito_Insecta_Culicidae_XP_039444992.1
74 Aedes_aegypti_yellow_fever_mosquito_Insecta_Culicidae_XP_001660577.1
75 Anopheles_stephensi_Asian_malaria_mosquito_Insecta_Culicidae_XP_035910436.1
76 Anopheles_coluzzii_mosquitos_Insecta_Culicidae_XP_040223562.1
77 Anopheles_arabiensis_mosquitos_Insecta_Culicidae_XP_040154970.1
78 Agrilus_planipennis_emerald_ash_borer_Insecta_Buprestidae_XP_018323607.1
79 Anopheles_albimanus_mosquitos_Insecta_Culicidae_XP_035793012.1
80 Glossina_fuscipes_tsetse_fly_Insecta_Glossinidae_XP_037879789.1
81 Drosophila_ficusphila_flies_Insecta_Drosophilidae_XP_017043330.1
82 Drosophila_albomicans_flies_Insecta_Drosophilidae_XP_034106294.1
83 Drosophila_virilis_flies_Insecta_Drosophilidae_XP_002046538.1
84 Drosophila_novamexicana_flies_Insecta_Drosophilidae_XP_030567850.1
85 Drosophila_obscura_flies_Insecta_Drosophilidae_XP_022215327.1
86 Drosophila_miranda_flies_Insecta_Drosophilidae_XP_017137713.1
87 Drosophila_pseudoobscura_flies_Insecta_Drosophilidae_XP_001352531.1
88 Drosophila_persimilis_flies_Insecta_Drosophilidae_XP_002024975.1
89 Drosophila_subobscura_flies_Insecta_Drosophilidae_XP_034655421.1
90 Drosophila_guanche_flies_Insecta_Drosophilidae_XP_034136271.1
91 Drosophila_hydei_flies_Insecta_Drosophilidae_XP_023167159.1
92 Drosophila_navojoa_flies_Insecta_Drosophilidae_XP_030244352.1
93 Drosophila_arizonae_Insecta_Drosophilidae_XP_017863552.1
94 Drosophila_mojavensis_flies_Insecta_Drosophilidae_XP_002011961.1
95 Scaptodrosophila_lebanonensis_flies_Insecta_Drosophilidae_XP_030384942.1
96 Drosophila_willistoni_flies_Insecta_Drosophilidae_XP_002062067.1
97 Drosophila_serrata_flies_Insecta_Drosophilidae_XP_020817122.1
98 Drosophila_kikkawai_flies_Insecta_Drosophilidae_XP_017028155.1
99 Drosophila_elegans_flies_Insecta_Drosophilidae_XP_017127571.1
100 Drosophila_ananassae_Insecta_Drosophilidae_XP_001957236.1
101 Drosophila_bipectinata_flies_Insecta_Drosophilidae_XP_017098422.1
102 Drosophila_biarmipes_flies_Insecta_Drosophilidae_XP_016966257.1
103 Drosophila_melanogaster_fruit_fly_Insecta_Drosophilidae_NP_524675.1
104 Drosophila_mauritiana_flies_Insecta_Drosophilidae_XP_033157475.1
105 Drosophila_sechellia_flies_Insecta_Drosophilidae_XP_032573482.1
106 Drosophila_simulans_flies_Insecta_Drosophilidae_XP_016032127.1
107 Drosophila_takahashii_flies_Insecta_Drosophilidae_XP_017015766.1
108 Drosophila_suzukii_flies_Insecta_Drosophilidae_XP_016926671.2
109 Drosophila_subpulchrella_flies_Insecta_Drosophilidae_XP_037721855.1
110 Drosophila_rhopaloa_flies_Insecta_Drosophilidae_XP_016971773.1
111 Drosophila_erecta_flies_Insecta_Drosophilidae_XP_001973181.1
112 Drosophila_eugracilis_flies_Insecta_Drosophilidae_XP_017081105.1
113 Drosophila_santomea_flies_Insecta_Drosophilidae_XP_039484920.1
114 Drosophila_yakuba_flies_Insecta_Drosophilidae_XP_002095770.2
115 Diachasma_alloeum_wasps_ants_and_bees_Insecta_Braconidae_XP_015116816.1
116 Fopius_arisanus_wasps_ants_and_bees_Insecta_Braconidae_XP_011300092.1
117 Ctenocephalides_felis_cat_flea_Insecta_Pulicidae_XP_026466515.1
118 Musca_domestica_house_fly_Insecta_Muscidae_XP_005188107.1
119 Lucilia_cuprina_Australian_sheep_blowfly_Insecta_Calliphoridae_XP_023300726.1
120 Lucilia_sericata_common_green_bottle_fly_Insecta_Calliphoridae_XP_037817741.1

```
		Melanaphis_sacchari_aphids_Insecta_Aphididae_XP_025208423.1
		Rhopalosiphum_maidis_corn_leaf_aphid_Insecta_Aphididae_XP_026815263.1
		Acyrthosiphon_pisum_pea_aphid_Insecta_Aphididae_XP_008182079.1
		Myzus_persicae_Insecta_Aphididae_XP_022164622.1
		Aphis_gossypii_cotton_aphid_Insecta_Aphididae_XP_027838628.1
		Contarinia_nasturtii_swede_midge_Insecta_Cecidomyiidae_XP_031638990.1
		Teleopsis_dalmanni_flies_Insecta_Diopsidae_XP_037938999.1
		Hermetia_illucens_flies_Insecta_Stratiomyidae_XP_037907221.1
		Orussus_abietinus_hymenopterans_Insecta_Orussidae_XP_012284196.1
		Sipha_flava_yellow_sugarcane_aphid_Insecta_Aphididae_XP_025415472.1
		Diuraphis_noxia_Russian_wheat_aphid_Insecta_Aphididae_XP_015363507.1
		Maniola_hyperantus_ringlet_Insecta_Nymphalidae_XP_034834687.1
		Pieris_rapae_Insecta_Pieridae_XP_022119342.1
		Pararge_aegeria_specked_wood_butterfly_Insecta_Nymphalidae_XP_039756098.1
		Vanessa_tameamea_butterflies_Insecta_Nymphalidae_XP_026495996.1
		Bombyx_mori_domestic_silkworm_Insecta_Bombycidae_XP_004923967.2
		Bombyx_mandarina_wild_silkworm_Insecta_Bombycidae_XP_028040643.1
		Megalopta_genalis_bees_Insecta_Halictidae_XP_033324989.1
		Rhagoletis_pomonella_apple_maggot_Insecta_Tephritidae_XP_036338294.1
		Rhagoletis_zephyria_snowberry_fruit_fly_Insecta_Tephritidae_XP_017477828.1
		Bactrocera_oleae_Insecta_Tephritidae_XP_014098751.1
		Bactrocera_dorsalis_oriental_fruit_fly_Insecta_Tephritidae_XP_011207420.1
		Bactrocera_tryoni_Queensland_fruit_fly_Insecta_Tephritidae_XP_039963487.1
		Zeugodacus_cucurbitae_Insecta_Tephritidae_XP_011195917.1
		Bactrocera_latifrons_flies_Insecta_Tephritidae_XP_018804350.1
		Ceratitis_capitata_Mediterranean_fruit_fly_Insecta_Tephritidae_XP_004525421.1
		Bicyclus_anynana_squinting_bush_brown_Insecta_Nymphalidae_XP_023942779.1
		Amyelois_transitella_moths_Insecta_Pyralidae_XP_013190181.1
		Zerene_cesonia_dogface_butterfly_Insecta_Pieridae_XP_038222544.1
		Trichoplusia_ni_cabbage_looper_Insecta_Noctuidae_XP_026741493.1
		Galleria_mellonella_greater_wax_moth_Insecta_Pyralidae_XP_026756211.1
		Helicoverpa_armigera_cotton_bollworm_Insecta_Noctuidae_XP_021197447.1
		Spodoptera_frugiperda_fall_armyworm_Insecta_Noctuidae_XP_035443593.1
		Hyposmocoma_kahamanoa_moths_Insecta_Cosmopterigidae_XP_026321351.1
		Onthophagus_taurus_beetles_Insecta_Scarabaeidae_XP_022904580.1
		Thrips_palmi_thrips_Insecta_Thripidae_XP_034232969.1
		Manduca_sexta_tobacco_hornworm_Insecta_Sphingidae_XP_037292989.1
		Cimex_lectularius_bed_bug_Insecta_Cimicidae_XP_014239452.1
		Bradysia_coprophila_flies_Insecta_Sciaridae_XP_037039073.1
		Chelonus_insularis_wasps_ants_and_bees_Insecta_Braconidae_XP_034946971.1
		Tribolium_castaneum_red_flour_beetle_Insecta_Tenebrionidae_XP_966394.2
		Formica_exsecta_ants_Insecta_Formicidae_XP_029670960.1
		Leptinotarsa_decemlineata_Colorado_potato_beetle_Insecta_Chrysomelidae_XP_023015182.1
		;
end;
begin trees;
	translate
		1	Homo_sapiens_human_Mammalia_Hominidae_NP_002624.2,
		2	Stomoxys_calcitrans_stable_fly_Insecta_Muscidae_XP_013103995.1,
		3	Nomia_melanderi_Alkali_bee_Insecta_Halictidae_XP_031842685.1,
		4	Folsomia_candida_springtails_Collembola_Isotomidae_XP_021955268.1,
		5	Harpegnathos_saltator_Jerdons_jumping_ant_Insecta_Formicidae_XP_011140815.1,
		6	Apis_dorsata_giant_honeybee_Insecta_Apidae_XP_006609201.1,
		7	Linepithema_humile_Argentine_ant_Insecta_Formicidae_XP_012224343.1,
		8	Acromyrmex_echinatior_Panamanian_leafcutter_ant_Insecta_Formicidae_XP_011055210.1,
		9	Mus_musculus_house_mouse_Mammalia_Muridae_NP_082408.3,
		10	Sitophilus_oryzae_Insecta_Curculionidae_XP_030749057.1,
		11	Nylanderia_fulva_ants_Insecta_Formicidae_XP_029173334.1,
		12	Pseudomyrmex_gracilis_ants_Insecta_Formicidae_XP_020289773.1,
		13	Pediculus_humanus_corporis_human_body_louse_Insecta_Pediculidae_XP_002424860.1,
		14	Pogonomyrmex_barbatus_red_harvester_ant_Insecta_Formicidae_XP_011641679.1,
		15	Monomorium_pharaonis_pharaoh_ant_Insecta_Formicidae_XP_012536222.1,
		16	Polistes_canadensis_wasps_ants_and_bees_Insecta_Vespidae_XP_014610425.1,
```

```
→→17→Camponotus_floridanus_Florida_carpenter_ant_Insecta_Formicidae_XP_011254716.1,
→→18→Zootermopsis_nevadensis_termites_Insecta_Termopsidae_XP_021927166.1,
→→19→Papilio_xuthus_Asian_swallowtail_Insecta_Papilionidae_XP_013166807.1,
→→20→Dendroctonus_ponderosae_Insecta_Curculionidae_XP_019759060.1,
→→21→Vollenhovia_emeryi_ants_Insecta_Formicidae_XP_011864933.1,
→→22→Vespa_mandarinia_Asian_giant_hornet_Insecta_Vespidae_XP_035737949.1,
→→23→Polistes_dominula_European_paper_wasp_Insecta_Vespidae_XP_015187969.1,
→→24→Microplitis_demolitor_wasps_ants_and_bees_Insecta_Braconidae_XP_014296476.1,
→→25→Dinoponera_quadriceps_ants_Insecta_Formicidae_XP_014488664.1,
→→26→Trachymyrmex_zeteki_ants_Insecta_Formicidae_XP_018308784.1,
→→27→Cyphomyrmex_costatus_ants_Insecta_Formicidae_XP_018405593.1,
→→28→Trachymyrmex_cornetzi_ants_Insecta_Formicidae_XP_018364082.1,
→→29→Temnothorax_curvispinosus_ants_Insecta_Formicidae_XP_024879566.1,
→→30→Wasmannia_auropunctata_little_fire_ant_Insecta_Formicidae_XP_011693239.1,
→→31→Trachymyrmex_septentrionalis_ants_Insecta_Formicidae_XP_018344009.1,
→→32→Atta_colombica_ants_Insecta_Formicidae_XP_018044394.1,
→→33→Athalia_rosae_Insecta_Tenthredinidae_XP_012267628.1,
→→34→Papilio_machaon_common_yellow_swallowtail_Insecta_Papilionidae_XP_014361343.1,
→→35→Anoplophora_glabripennis_Asian_longhorned_beetle_Insecta_Cerambycidae_XP_023309822.1,
→→36→Dufourea_novaeangliae_Insecta_Halictidae_XP_015433181.1,
→→37→Ooceraea_biroi_clonal_raider_ant_Insecta_Formicidae_XP_011340302.2,
→→38→Habropoda_laboriosa_bees_Insecta_Apidae_XP_017794782.1,
→→39→Ceratina_calcarata_bees_Insecta_Apidae_XP_026673966.1,
→→40→Osmia_bicornis_bicornis_red_mason_bee_Insecta_Megachilidae_XP_029036921.1,
→→41→Osmia_lignaria_orchard_mason_bee_Insecta_Megachilidae_XP_034193789.1,
→→42→Cephus_cinctus_wheat_stem_sawfly_Insecta_Cephidae_XP_015597724.1,
→→43→Apis_florea_little_honeybee_Insecta_Apidae_XP_003695955.1,
→→44→Apis_cerana_Asiatic_honeybee_Insecta_Apidae_XP_016912531.1,
→→45→Eufriesea_mexicana_bees_Insecta_Apidae_XP_017753798.1,
→→46→Bombus_terrestris_bufftailed_bumblebee_Insecta_Apidae_XP_003401536.1,
→→47→Bombus_bifarius_bees_Insecta_Apidae_XP_033300165.1,
→→48→Bombus_vosnesenskii_bees_Insecta_Apidae_XP_033344194.1,
→→49→Diabrotica_virgifera_virgifera_western_corn_rootworm_Insecta_Chrysomelidae_XP_028143837.1,
→→50→Apis_mellifera_honey_bee_Insecta_Apidae_XP_395366.2,
→→51→Belonocnema_treatae_Insecta_Cynipidae_XP_033214709.1,
→→52→Ceratosolen_solmsi_marchali_wasps_ants_and_bees_Insecta_Agaonidae_XP_011498014.1,
→→53→Copidosoma_floridanum_wasps_ants_and_bees_Insecta_Encyrtidae_XP_014206533.1,
→→54→Trichogramma_pretiosum_wasps_ants_and_bees_Insecta_Trichogrammatidae_XP_014236333.1,
→→55→Nasonia_vitripennis_jewel_wasp_Insecta_Pteromalidae_XP_001608147.1,
→→56→Odontomachus_brunneus_ants_Insecta_Formicidae_XP_032684609.1,
→→57→Drosophila_grimshawi_flies_Insecta_Drosophilidae_XP_001984409.1,
→→58→Megachile_rotundata_alfalfa_leafcutting_bee_Insecta_Megachilidae_XP_003699636.1,
→→59→Cryptotermes_secundus_termites_Insecta_Kalotermitidae_XP_023719654.1,
→→60→Bemisia_tabaci_sweet_potato_whitefly_Insecta_Aleyrodidae_XP_018903307.1,
→→61→Photinus_pyralis_common_eastern_firefly_Insecta_Lampyridae_XP_031330233.1,
→→62→Drosophila_busckii_flies_Insecta_Drosophilidae_XP_017844450.1,
→→63→Nicrophorus_vespilloides_beetles_Insecta_Silphidae_XP_017786364.1,
→→64→Aedes_albopictus_Asian_tiger_mosquito_Insecta_Culicidae_XP_019543107.2,
→→65→Nilaparvata_lugens_brown_planthopper_Insecta_Delphacidae_XP_039286781.1,
→→66→Culex_quinquefasciatus_southern_house_mosquito_Insecta_Culicidae_XP_001847230.1,
→→67→Culex_pipiens_pallens_northern_house_mosquito_Insecta_Culicidae_XP_039444992.1
```

```
,
		68	Aedes_aegypti_yellow_fever_mosquito_Insecta_Culicidae_XP_001660577.1,
		69	Anopheles_stephensi_Asian_malaria_mosquito_Insecta_Culicidae_XP_035910436.1,
		70	Anopheles_coluzzii_mosquitos_Insecta_Culicidae_XP_040223562.1,
		71	Anopheles_arabiensis_mosquitos_Insecta_Culicidae_XP_040154970.1,
		72	Agrilus_planipennis_emerald_ash_borer_Insecta_Buprestidae_XP_018323607.1,
		73	Anopheles_albimanus_mosquitos_Insecta_Culicidae_XP_035793012.1,
		74	Glossina_fuscipes_tsetse_fly_Insecta_Glossinidae_XP_037879789.1,
		75	Drosophila_ficusphila_flies_Insecta_Drosophilidae_XP_017043330.1,
		76	Drosophila_albomicans_flies_Insecta_Drosophilidae_XP_034106294.1,
		77	Drosophila_virilis_flies_Insecta_Drosophilidae_XP_002046538.1,
		78	Drosophila_novamexicana_flies_Insecta_Drosophilidae_XP_030567850.1,
		79	Drosophila_obscura_flies_Insecta_Drosophilidae_XP_022215327.1,
		80	Drosophila_miranda_flies_Insecta_Drosophilidae_XP_017137713.1,
		81	Drosophila_pseudoobscura_flies_Insecta_Drosophilidae_XP_001352531.1,
		82	Drosophila_persimilis_flies_Insecta_Drosophilidae_XP_002024975.1,
		83	Drosophila_subobscura_flies_Insecta_Drosophilidae_XP_034655421.1,
		84	Drosophila_guanche_flies_Insecta_Drosophilidae_XP_034136271.1,
		85	Drosophila_hydei_flies_Insecta_Drosophilidae_XP_023167159.1,
		86	Drosophila_navojoa_flies_Insecta_Drosophilidae_XP_030244352.1,
		87	Drosophila_arizonae_Insecta_Drosophilidae_XP_017863552.1,
		88	Drosophila_mojavensis_flies_Insecta_Drosophilidae_XP_002011961.1,
		89	Scaptodrosophila_lebanonensis_flies_Insecta_Drosophilidae_XP_030384942.1,
		90	Drosophila_willistoni_flies_Insecta_Drosophilidae_XP_002062067.1,
		91	Drosophila_serrata_flies_Insecta_Drosophilidae_XP_020817122.1,
		92	Drosophila_kikkawai_flies_Insecta_Drosophilidae_XP_017028155.1,
		93	Drosophila_elegans_flies_Insecta_Drosophilidae_XP_017127571.1,
		94	Drosophila_ananassae_Insecta_Drosophilidae_XP_001957236.1,
		95	Drosophila_bipectinata_flies_Insecta_Drosophilidae_XP_017098422.1,
		96	Drosophila_biarmipes_flies_Insecta_Drosophilidae_XP_016966257.1,
		97	Drosophila_melanogaster_fruit_fly_Insecta_Drosophilidae_NP_524675.1,
		98	Drosophila_mauritiana_flies_Insecta_Drosophilidae_XP_033157475.1,
		99	Drosophila_sechellia_flies_Insecta_Drosophilidae_XP_032573482.1,
		100	Drosophila_simulans_flies_Insecta_Drosophilidae_XP_016032127.1,
		101	Drosophila_takahashii_flies_Insecta_Drosophilidae_XP_017015766.1,
		102	Drosophila_suzukii_flies_Insecta_Drosophilidae_XP_016926671.2,
		103	Drosophila_subpulchrella_flies_Insecta_Drosophilidae_XP_037721855.1,
		104	Drosophila_rhopaloa_flies_Insecta_Drosophilidae_XP_016971773.1,
		105	Drosophila_erecta_flies_Insecta_Drosophilidae_XP_001973181.1,
		106	Drosophila_eugracilis_flies_Insecta_Drosophilidae_XP_017081105.1,
		107	Drosophila_santomea_flies_Insecta_Drosophilidae_XP_039484920.1,
		108	Drosophila_yakuba_flies_Insecta_Drosophilidae_XP_002095770.2,
		109	Diachasma_alloeum_wasps_ants_and_bees_Insecta_Braconidae_XP_015116816.1,
		110	Fopius_arisanus_wasps_ants_and_bees_Insecta_Braconidae_XP_011300092.1,
		111	Ctenocephalides_felis_cat_flea_Insecta_Pulicidae_XP_026466515.1,
		112	Musca_domestica_house_fly_Insecta_Muscidae_XP_005188107.1,
		113	Lucilia_cuprina_Australian_sheep_blowfly_Insecta_Calliphoridae_XP_023300726.1,
		114	Lucilia_sericata_common_green_bottle_fly_Insecta_Calliphoridae_XP_037817741.1,
		115	Melanaphis_sacchari_aphids_Insecta_Aphididae_XP_025208423.1,
		116	Rhopalosiphum_maidis_corn_leaf_aphid_Insecta_Aphididae_XP_026815263.1,
		117	Acyrthosiphon_pisum_pea_aphid_Insecta_Aphididae_XP_008182079.1,
		118	Myzus_persicae_Insecta_Aphididae_XP_022164622.1,
		119	Aphis_gossypii_cotton_aphid_Insecta_Aphididae_XP_027838628.1,
		120	Contarinia_nasturtii_swede_midge_Insecta_Cecidomyiidae_XP_031638990.1,
		121	Teleopsis_dalmanni_flies_Insecta_Diopsidae_XP_037938999.1,
		122	Hermetia_illucens_flies_Insecta_Stratiomyidae_XP_037907221.1,
		123	Orussus_abietinus_hymenopterans_Insecta_Orussidae_XP_012284196.1,
		124	Sipha_flava_yellow_sugarcane_aphid_Insecta_Aphididae_XP_025415472.1,
		125	Diuraphis_noxia_Russian_wheat_aphid_Insecta_Aphididae_XP_015363507.1,
		126	Maniola_hyperantus_ringlet_Insecta_Nymphalidae_XP_034834687.1,
		127	Pieris_rapae_Insecta_Pieridae_XP_022119342.1,
		128	Pararge_aegeria_specked_wood_butterfly_Insecta_Nymphalidae_XP_039756098.1,
		129	Vanessa_tameamea_butterflies_Insecta_Nymphalidae_XP_026495996.1,
		130	Bombyx_mori_domestic_silkworm_Insecta_Bombycidae_XP_004923967.2,
		131	Bombyx_mandarina_wild_silkworm_Insecta_Bombycidae_XP_028040643.1,
```

```
		132	Megalopta_genalis_bees_Insecta_Halictidae_XP_033324989.1,
		133	Rhagoletis_pomonella_apple_maggot_Insecta_Tephritidae_XP_036338294.1,
		134	Rhagoletis_zephyria_snowberry_fruit_fly_Insecta_Tephritidae_XP_017477828.1,
		135	Bactrocera_oleae_Insecta_Tephritidae_XP_014098751.1,
		136	Bactrocera_dorsalis_oriental_fruit_fly_Insecta_Tephritidae_XP_011207420.1,
		137	Bactrocera_tryoni_Queensland_fruit_fly_Insecta_Tephritidae_XP_039963487.1,
		138	Zeugodacus_cucurbitae_Insecta_Tephritidae_XP_011195917.1,
		139	Bactrocera_latifrons_flies_Insecta_Tephritidae_XP_018804350.1,
		140	Ceratitis_capitata_Mediterranean_fruit_fly_Insecta_Tephritidae_XP_004525421.1,
		141	Bicyclus_anynana_squinting_bush_brown_Insecta_Nymphalidae_XP_023942779.1,
		142	Amyelois_transitella_moths_Insecta_Pyralidae_XP_013190181.1,
		143	Zerene_cesonia_dogface_butterfly_Insecta_Pieridae_XP_038222544.1,
		144	Trichoplusia_ni_cabbage_looper_Insecta_Noctuidae_XP_026741493.1,
		145	Galleria_mellonella_greater_wax_moth_Insecta_Pyralidae_XP_026756211.1,
		146	Helicoverpa_armigera_cotton_bollworm_Insecta_Noctuidae_XP_021197447.1,
		147	Spodoptera_frugiperda_fall_armyworm_Insecta_Noctuidae_XP_035443593.1,
		148	Hyposmocoma_kahamanoa_moths_Insecta_Cosmopterigidae_XP_026321351.1,
		149	Onthophagus_taurus_beetles_Insecta_Scarabaeidae_XP_022904580.1,
		150	Thrips_palmi_thrips_Insecta_Thripidae_XP_034232969.1,
		151	Manduca_sexta_tobacco_hornworm_Insecta_Sphingidae_XP_037292989.1,
		152	Cimex_lectularius_bed_bug_Insecta_Cimicidae_XP_014239452.1,
		153	Bradysia_coprophila_flies_Insecta_Sciaridae_XP_037039073.1,
		154	Chelonus_insularis_wasps_ants_and_bees_Insecta_Braconidae_XP_034946971.1,
		155	Tribolium_castaneum_red_flour_beetle_Insecta_Tenebrionidae_XP_966394.2,
		156	Formica_exsecta_ants_Insecta_Formicidae_XP_029670960.1,
		157	Leptinotarsa_decemlineata_Colorado_potato_beetle_Insecta_Chrysomelidae_XP_023015182.1
		;
   [Note: This tree contains information on the topology, 
          branch lengths (if present), and the probability
          of the partition indicated by the branch.]
   tree con_50_majrule = (1:0.0608027,9:0.09245733,(((((((((((((((((((2:0.1522415,112:0.1383798)1.000:0.05417401,(113:0.04089951,114:0.03783071)1.000:0.1343739)0.999:0.03773238,74:0.5261902)1.000:0.06726657,((120:0.4681043,153:0.3898911)0.996:0.121829,122:0.3833157)0.847:0.1591415,(121:0.3535142,(((133:0.00785787,134:0.008905137)1.000:0.2033869,140:0.1230305)0.739:0.04044319,((135:0.03742463,((136:0.02287777,137:0.0172045)0.998:0.005754885,139:0.02143149)1.000:0.02561182)0.894:0.0216546,138:0.08071172)1.000:0.06960543)1.000:0.1907159)0.603:0.05883642)0.847:0.1242386,90:0.1518113)0.847:0.08676596,89:0.1744955)0.847:0.06962831,62:0.1772306)0.847:0.03130457,(57:0.1406667,76:0.1094968)0.984:0.02364176)0.846:0.02710069,(85:0.08181632,(86:0.04853252,(87:0.01042022,88:0.006980301)1.000:0.01303405)1.000:0.0871618)1.000:0.1351145)0.847:0.02748519,(77:0.02343715,78:0.01369453)1.000:0.06123097)0.847:0.07459004,((((((((75:0.09860162,(93:0.03298775,104:0.06008077)1.000:0.01557538)1.000:0.01407838,(((97:0.02281471,((98:0.01877038,100:0.009214679)1.000:0.005388523,99:0.01580091)1.000:0.009123711)1.000:0.01124446,(105:0.02838177,(107:0.008462588,108:0.009141723)1.000:0.02863063)1.000:0.01728525)1.000:0.05804061,106:0.1064552)1.000:0.01816513)1.000:0.01513417,(102:0.01859091,103:0.01501938)1.000:0.0113599)1.000:0.009503812,96:0.02802617)1.000:0.01233743,101:0.04189087)1.000:0.02545834,(94:0.05722168,95:0.03720457)1.000:0.1137065)0.995:0.0240454,(91:0.0520987,92:0.02742845)1.000:0.05815435)1.000:0.05790859,((79:0.03550577,(80:0.01121321,(81:0.005747658,82:0.002773127)1.000:0.00494465)1.000:0.04205873)0.999:0.01409615,(83:0.01485008,84:0.01415174)1.000:0.02708877)1.000:0.08406978)1.000:0.03857205)0.847:0.1091222,(((64:0.09819406,68:0.1061991)1.000:0.09623464,(66:0.02501438,67:0.02018763)1.000:0.1473757)1.000:0.07133136,((69:0.1253159,(70:0.01099979,71:0.008334865)1.000:0.09769681)1.000:0.06173545,73:0.1441069)1.000:0.1070721)1.000:0.1158717)1.000:0.2834479,(((((((10:0.338429,20:0.2774145)1.000:0.1913268,(35:0.2620092,(49:0.2371423,157:0.4906881)0.950:0.08842715)1.000:0.08899118)1.000:0.1292693,155:0.4885195)0.755:0.03526194,(61:0.3603944,72:0.499229)0.844:0.08135389)0.847:0.0610305,63:0.4679288)0.990:0.07895343,149:0.9011808)1.000:0.197933,111:1.389162)0.713:0.05721566)0.917:0.09511586,((((((((19:0.07583306,34:0.06986532)1.000:0.2165244,129:0.1881747)1.000:0.05889769,((126:0.1300619,128:0.1389541)1.000:0.07692805,141:0.1011548)1.000:0.0916304)1.000:0.04601632,(127:0.2844747,143:0.2165785)1.000:0.1601697)1.000:0.06066633,((130:0.01286322,131:0.0123729)1.000:0.3344112,151:0.2208227)1.000:0.04516747)0.917:0.02351666,(144:0.1821142,(146:0.1697498,147:0.1522359)1.000:0.04526733)1.000:0.07599201)0.725:0.0345615,(142:0.244075,148:0.3012199)
```

```
0.811:0.06231832)0.534:0.05655389,145:0.264644)1.000:0.2870866)0.996:0.2013791,((((
((((((3:0.1308743,36:0.09603669)0.999:0.03473229,132:0.106744)1.000:0.09092754,((((
((((6:0.01934941,44:0.01631549)0.986:0.005595155,50:0.02304556)0.984:0.006838181,43
:0.01554218)1.000:0.09665306,45:0.09131043)0.739:0.01284142,(46:0.007909884,(47:0.0
02366284,48:0.001373636)1.000:0.003644494)1.000:0.07067312)1.000:0.02916636,38:0.08
418011)1.000:0.01698671,39:0.3675439)1.000:0.04593881,((40:0.007440492,41:0.0101147
3)1.000:0.1047657,58:0.06992283)1.000:0.05158156)1.000:0.05285686)1.000:0.1491919,(
(16:0.02228169,23:0.03178249)1.000:0.0592053,22:0.05746253)1.000:0.2591004)0.999:0.
05434792,((5:0.0977341,(25:0.1505455,56:0.09508833)0.571:0.01951545)1.000:0.0849352
4,((((7:0.1316676,12:0.1704683)0.984:0.02549432,37:0.16513)1.000:0.02429056,((((((8
:0.01629505,28:0.02734035)0.977:0.00384722,(31:0.01495146,32:0.0241132)0.950:0.0029
20262)1.000:0.01865096,26:0.0257306)1.000:0.0102861,27:0.05101623)1.000:0.06156152,
((21:0.08406142,29:0.04680583)0.930:0.01551642,30:0.05167178)0.960:0.01708479)0.965
:0.01282225,(14:0.1021041,15:0.1059921)0.634:0.0141179)1.000:0.04996724)0.993:0.023
56388,(11:0.06698633,(17:0.05340993,156:0.07650684)0.621:0.01239276)1.000:0.0516079
6)1.000:0.0581727)1.000:0.2371296)1.000:0.1555626,(51:0.4719538,(52:0.2722088,((53:
0.4473637,54:0.3279321)0.999:0.07741826,55:0.242623)0.988:0.04757268)1.000:0.169206
5)1.000:0.09904578)0.968:0.06310286,33:0.5384843)0.657:0.04149707,((24:0.5579541,15
4:0.3959763)0.863:0.08337691,(109:0.2043907,110:0.176394)1.000:0.2308812)1.000:0.17
80584)0.981:0.09711768,42:0.4386144)0.636:0.05044618,123:0.4129148)1.000:0.4827264,
150:0.4460313)0.741:0.05757419,((60:0.495523,(((115:0.05859696,(116:0.04506685,119:
0.04231964)0.994:0.008830393)1.000:0.0260503,((117:0.02792799,125:0.04676028)0.989:
0.007496751,118:0.0234532)1.000:0.01819706)1.000:0.1224092,124:0.09811425)1.000:0.6
063299)0.779:0.1391403,65:0.5842567)0.998:0.1083386)0.582:0.0668403,(13:1.009998,(1
8:0.1369988,59:0.1485139)1.000:0.6773605)0.992:0.1687938)0.843:0.1213807,152:1.4181
63)0.996:0.2368109,4:1.368983)1.000:0.7008861);

[Note: This tree contains information only on the topology
          and branch lengths (median of the posterior probability density).]
tree con_50_majrule = 
(1:0.0608027,9:0.09245733,(((((((((((((((((2:0.1522415,112:0.1383798):0.05417401,
(113:0.04089951,114:0.03783071):0.1343739):0.03773238,74:0.5261902):0.06726657,((12
0:0.4681043,153:0.3898911):0.121829,122:0.3833157):0.1591415,(121:0.3535142,(((133:
0.00785787,134:0.008905137):0.2033869,140:0.1230305):0.04044319,((135:0.03742463,((
136:0.02287777,137:0.0172045):0.005754885,139:0.02143149):0.02561182):0.0216546,138
:0.08071172):0.06960543):0.1907159):0.05883642):0.1242386,90:0.1518113):0.08676596,
89:0.1744955):0.06962831,62:0.1772306):0.03130457,(57:0.1406667,76:0.1094968):0.023
64176):0.02710069,(85:0.08181632,(86:0.04853252,(87:0.01042022,88:0.006980301):0.01
303405):0.0871618):0.1351145):0.02748519,(77:0.02343715,78:0.01369453):0.06123097):
0.07459004,((((((((75:0.09860162,(93:0.03298775,104:0.06008077):0.01557538):0.01407
838,(((97:0.02281471,((98:0.01877038,100:0.009214679):0.005388523,99:0.01580091):0.
009123711):0.01124446,(105:0.02838177,(107:0.008462588,108:0.009141723):0.02863063)
:0.01728525):0.05804061,106:0.1064552):0.01816513):0.01513417,(102:0.01859091,103:0
.01501938):0.0113599):0.009503812,96:0.02802617):0.01233743,101:0.04189087):0.02545
834,(94:0.05722168,95:0.03720457):0.1137065):0.0240454,(91:0.0520987,92:0.02742845)
:0.05815435):0.05790859,((79:0.03550577,(80:0.01121321,(81:0.005747658,82:0.0027731
27):0.00494465):0.04205873):0.01409615,(83:0.01485008,84:0.01415174):0.02708877):0.
08406978):0.03857205):0.1091222,(((64:0.09819406,68:0.1061991):0.09623464,(66:0.025
01438,67:0.02018763):0.1473757):0.07133136,((69:0.1253159,(70:0.01099979,71:0.00833
4865):0.09769681):0.06173545,73:0.1441069):0.1070721):0.1158717):0.2834479,((((((1
0:0.338429,20:0.2774145):0.1913268,(35:0.2620092,(49:0.2371423,157:0.4906881):0.088
42715):0.08899118):0.1292693,155:0.4885195):0.03526194,(61:0.3603944,72:0.499229):0
.08135389):0.0610305,63:0.4679288):0.07895343,149:0.9011808):0.197933,111:1.389162)
:0.05721566):0.09511586,((((((((19:0.07583306,34:0.06986532):0.2165244,129:0.188174
7):0.05889769,((126:0.1300619,128:0.1389541):0.07692805,141:0.1011548):0.0916304):0
.04601632,(127:0.2844747,143:0.2165785):0.1601697):0.06066633,((130:0.01286322,131:
0.0123729):0.3344112,151:0.2208227):0.04516747):0.02351666,(144:0.1821142,(146:0.16
97498,147:0.1522359):0.04526733):0.07599201):0.0345615,(142:0.244075,148:0.3012199)
:0.06231832):0.05655389,145:0.264644):0.2870866):0.2013791,((((((((((3:0.1308743,36
:0.09603669):0.03473229,132:0.106744):0.09092754,((((((((6:0.01934941,44:0.01631549
):0.005595155,50:0.02304556):0.006838181,43:0.01554218):0.09665306,45:0.09131043):0
.01284142,(46:0.007909884,(47:0.002366284,48:0.001373636):0.003644494):0.07067312):
0.02916636,38:0.08418011):0.01698671,39:0.3675439):0.04593881,((40:0.007440492,41:0
.01011473):0.1047657,58:0.06992283):0.05158156):0.05285686):0.1491919,((16:0.022281
69,23:0.03178249):0.0592053,22:0.05746253):0.2591004):0.05434792,((5:0.0977341,(25:
0.1505455,56:0.09508833):0.01951545):0.08493524,((((7:0.1316676,12:0.1704683):0.025
49432,37:0.16513):0.02429056,((((((8:0.01629505,28:0.02734035):0.00384722,(31:0.014
95146,32:0.0241132):0.002920262):0.01865096,26:0.0257306):0.0102861,27:0.05101623):
0.06156152,((21:0.08406142,29:0.04680583):0.01551642,30:0.05167178):0.01708479):0.0
1282225,(14:0.1021041,15:0.1059921):0.0141179):0.04996724):0.02356388,(11:0.0669863
3,(17:0.05340993,156:0.07650684):0.01239276):0.05160796):0.0581727):0.2371296):0.15
55626,(51:0.4719538,(52:0.2722088,((53:0.4473637,54:0.3279321):0.07741826,55:0.2426
23):0.04757268):0.1692065):0.09904578):0.06310286,33:0.5384843):0.04149707,((24:0.5
```

```
579541,154:0.3959763):0.08337691,(109:0.2043907,110:0.176394):0.2308812):0.1780584)
:0.09711768,42:0.4386144):0.05044618,123:0.4129148):0.4827264,150:0.4460313):0.0575
7419,((60:0.495523,(((115:0.05859696,(116:0.04506685,119:0.04231964):0.008830393):0
.0260503,((117:0.02792799,125:0.04676028):0.007496751,118:0.0234532):0.01819706):0.
1224092,124:0.09811425):0.6063299):0.1391403,65:0.5842567):0.1083386):0.0668403,(13
:1.009998,(18:0.1369988,59:0.1485139):0.6773605):0.1687938):0.1213807,152:1.418163)
:0.2368109,4:1.368983):0.7008861);LF
end;LF
```
