# Supplementary material for: Inferences on the evolution of the ascorbic acid synthesis pathway in insects using Phylogenetic Tree Collapser (PTC), a tool for the automated collapsing of phylogenetic trees using taxonomic information
Source: J Integr Bioinform. 2024 Jul 24;21(2):20230051. doi: 10.1515/jib-2023-0051 (PMC11377030; doi:10.1515/jib-2023-0051)
Supplement: Supplementary file 1 — Supplementary Material Details [file j_jib-2023-0051_suppl_001.zip › Supplementary_File_7_PGM2.con_PDF.pdf]

```
#NEXUS

[ID: 9201137380]
begin taxa;
	dimensions ntax=220;
	taxlabels
		Drosophila_hydei_flies_Insecta_Drosophilidae_XP_023161462.1
		Drosophila_hydei_flies_Insecta_Drosophilidae_XP_023172448.2
		Drosophila_hydei_flies_Insecta_Drosophilidae_XP_023171551.2
		Diabrotica_virgifera_virgifera_western_corn_rootworm_Insecta_Chrysomelidae_XP_028143025.1
		Drosophila_yakuba_flies_Insecta_Drosophilidae_XP_002091361.1
		Drosophila_yakuba_flies_Insecta_Drosophilidae_XP_002089741.2
		Fopius_arisanus_wasps_ants_and_bees_Insecta_Braconidae_XP_011310787.1
		Teleopsis_dalmanni_flies_Insecta_Diopsidae_XP_037929741.1
		Teleopsis_dalmanni_flies_Insecta_Diopsidae_XP_037949477.1
		Trichogramma_pretiosum_wasps_ants_and_bees_Insecta_Trichogrammatidae_XP_014238796.1
		Pseudomyrmex_gracilis_ants_Insecta_Formicidae_XP_020280661.1
		Cyphomyrmex_costatus_ants_Insecta_Formicidae_XP_018396236.1
		Sitophilus_oryzae_rice_weevil_Insecta_Curculionidae_XP_030757591.1
		Sitophilus_oryzae_rice_weevil_Insecta_Curculionidae_XP_030768391.1
		Apis_dorsata_giant_honeybee_Insecta_Apidae_XP_006623156.1
		Vespa_mandarinia_Asian_giant_hornet_Insecta_Vespidae_XP_035743739.1
		Atta_cephalotes_ants_Insecta_Formicidae_XP_012054188.1
		Drosophila_albomicans_flies_Insecta_Drosophilidae_XP_034108453.1
		Drosophila_albomicans_flies_Insecta_Drosophilidae_XP_034108654.1
		Drosophila_albomicans_flies_Insecta_Drosophilidae_XP_034107122.1
		Maniola_hyperantus_ringlet_Insecta_XP_034827204.1
		Apis_mellifera_honey_bee_Insecta_Apidae_XP_026297163.1
		Cimex_lectularius_bed_bug_Insecta_Cimicidae_XP_014251917.1
		Drosophila_sechellia_flies_Insecta_Drosophilidae_XP_002033901.1
		Drosophila_sechellia_flies_Insecta_Drosophilidae_XP_032572942.1
		Drosophila_melanogaster_fruit_fly_Insecta_Drosophilidae_NP_610992.2
		Drosophila_melanogaster_fruit_fly_Insecta_Drosophilidae_NP_610453.1
		Vollenhovia_emeryi_ants_Insecta_Formicidae_XP_011871365.1
		Drosophila_novamexicana_flies_Insecta_Drosophilidae_XP_030566323.1
		Drosophila_novamexicana_flies_Insecta_Drosophilidae_XP_030566424.1
		Drosophila_novamexicana_flies_Insecta_Drosophilidae_XP_030559958.1
		Drosophila_novamexicana_flies_Insecta_Drosophilidae_XP_030559753.1
		Spodoptera_frugiperda_fall_armyworm_Insecta_XP_035434313.1
		Spodoptera_frugiperda_fall_armyworm_Insecta_XP_035434146.1
		Scaptodrosophila_lebanonensis_flies_Insecta_Drosophilidae_XP_030387011.1
		Scaptodrosophila_lebanonensis_flies_Insecta_Drosophilidae_XP_030372628.1
		Pieris_rapae_cabbage_white_Insecta_Pieridae_XP_022123915.1
		Atta_colombica_ants_Insecta_Formicidae_XP_018046319.1
		Rhagoletis_zephyria_snowberry_fruit_fly_Insecta_Tephritidae_XP_017469128.1
		Drosophila_navojoa_flies_Insecta_Drosophilidae_XP_017959596.1
		Drosophila_navojoa_flies_Insecta_Drosophilidae_XP_017962256.1
		Drosophila_navojoa_flies_Insecta_Drosophilidae_XP_017962257.1
		Drosophila_navojoa_flies_Insecta_Drosophilidae_XP_017958700.1
		Acyrthosiphon_pisum_pea_aphid_Insecta_Aphididae_XP_008186737.1
		Anopheles_coluzzii_mosquitos_Insecta_Culicidae_XP_040231685.1
		Drosophila_mauritiana_flies_Insecta_Drosophilidae_XP_033155850.1
		Drosophila_mauritiana_flies_Insecta_Drosophilidae_XP_033155919.1
		Megachile_rotundata_alfalfa_leafcutting_bee_Insecta_Megachilidae_XP_003703795.1
		Trachymyrmex_cornetzi_ants_Insecta_Formicidae_XP_018364226.1
		Ctenocephalides_felis_cat_flea_Insecta_Pulicidae_XP_026473532.1
		Anoplophora_glabripennis_Asian_longhorned_beetle_Insecta_Cerambycidae_XP_018564394.1
		Drosophila_serrata_flies_Insecta_Drosophilidae_XP_020801284.1
		Drosophila_serrata_flies_Insecta_Drosophilidae_XP_020814606.1
		Anopheles_albimanus_mosquitos_Insecta_Culicidae_XP_035776653.1
		Apis_cerana_Asiatic_honeybee_Insecta_Apidae_XP_016922536.1
		Ooceraea_biroi_clonal_raider_ant_Insecta_Formicidae_XP_011339242.2
		Hyposmocoma_kahamanoa_moths_Insecta_Cosmopterigidae_XP_026315441.1
		Drosophila_rhopaloa_flies_Insecta_Drosophilidae_XP_016975014.1
		Drosophila_rhopaloa_flies_Insecta_Drosophilidae_XP_016971263.1
```

66 Ceratina_calcarata_bees_Insecta_Apidae_XP_017879794.2
67 Melanaphis_sacchari_aphids_Insecta_Aphididae_XP_025205583.1
68 Diuraphis_noxia_Russian_wheat_aphid_Insecta_Aphididae_XP_015378800.1
69 Nomia_melanderi_Alkali_bee_Insecta_Halictidae_XP_031839243.1
70 Drosophila_eugracilis_flies_Insecta_Drosophilidae_XP_017068721.1
71 Drosophila_eugracilis_flies_Insecta_Drosophilidae_XP_017066148.1
72 Osmia_bicornis_bicornis_red_mason_bee_Insecta_Megachilidae_XP_029048774.1
73 Lucilia_cuprina_Australian_sheep_blowfly_Insecta_Calliphoridae_XP_023307068.1
74 Drosophila_biarmipes_flies_Insecta_Drosophilidae_XP_016946573.1
75 Drosophila_biarmipes_flies_Insecta_Drosophilidae_XP_016968374.1
76 Drosophila_erecta_flies_Insecta_Drosophilidae_XP_001975581.1
77 Drosophila_erecta_flies_Insecta_Drosophilidae_XP_001970362.2
78 Drosophila_simulans_flies_Insecta_Drosophilidae_XP_016027660.1
79 Drosophila_simulans_flies_Insecta_Drosophilidae_XP_016026622.1
80 Nicrophorus_vespilloides_beetles_Insecta_Silphidae_XP_017781760.1
81 Thrips_palmi_thrips_Insecta_Thripidae_XP_034255598.1
82 Bactrocera_latifrons_flies_Insecta_Tephritidae_XP_018803639.1
83 Bactrocera_latifrons_flies_Insecta_Tephritidae_XP_018803564.1
84 Drosophila_suzukii_flies_Insecta_Drosophilidae_XP_016928030.1
85 Drosophila_suzukii_flies_Insecta_Drosophilidae_XP_016930474.1
86 Bombyx_mori_domestic_silkworm_Insecta_Bombycidae_XP_004929437.1
87 Bradysia_coprophila_flies_Insecta_Sciaridae_XP_037047063.1
88 Cryptotermes_secundus_termites_Insecta_Kalotermitidae_XP_033606653.1
89 Anopheles_stephensi_Asian_malaria_mosquito_Insecta_Culicidae_XP_035905304.1
90 Copidosoma_floridanum_wasps_ants_and_bees_Insecta_Encyrtidae_XP_014218420.1
91 Eufriesea_mexicana_bees_Insecta_Apidae_XP_017765990.1
92 Bactrocera_dorsalis_oriental_fruit_fly_Insecta_Tephritidae_XP_011198066.1
93 Bactrocera_dorsalis_oriental_fruit_fly_Insecta_Tephritidae_XP_011198068.1
94 Homo_sapiens_human_Dipnotetrapodomorpha_Hominidae_NP_060760.2
95 Homo_sapiens_human_Dipnotetrapodomorpha_Hominidae_NP_775853.2
96 Papilio_machaon_common_yellow_swallowtail_Insecta_Papilionidae_XP_014369384.1
97 Pogonomyrmex_barbatus_red_harvester_ant_Insecta_Formicidae_XP_025075767.1
98 Drosophila_miranda_flies_Insecta_Drosophilidae_XP_017150747.1
99 Drosophila_miranda_flies_Insecta_Drosophilidae_XP_033247062.1
100 Drosophila_miranda_flies_Insecta_Drosophilidae_XP_017147529.2
101 Drosophila_miranda_flies_Insecta_Drosophilidae_XP_033255654.1
102 Pediculus_humanus_corporis_human_body_louse_Insecta_Pediculidae_XP_002428808.1
103 Ceratosolen_solmsi_marchali_wasps_ants_and_bees_Insecta_Agaonidae_XP_011502643.1
104 Aedes_aegypti_yellow_fever_mosquito_Insecta_Culicidae_XP_001653384.1
105 Danaus_plexippus_plexippus_monarch_butterfly_Insecta_XP_032515592.1
106 Temnothorax_curvispinosus_ants_Insecta_Formicidae_XP_024878483.1
107 Drosophila_takahashii_flies_Insecta_Drosophilidae_XP_016993793.1
108 Drosophila_takahashii_flies_Insecta_Drosophilidae_XP_017015161.1
109 Chelonus_insularis_wasps_ants_and_bees_Insecta_Braconidae_XP_034944353.1
110 Formica_exsecta_ants_Insecta_Formicidae_XP_029678652.1
111 Rhopalosiphum_maidis_corn_leaf_aphid_Insecta_Aphididae_XP_026807396.1
112 Drosophila_ananassae_flies_Insecta_Drosophilidae_XP_001959966.1
113 Drosophila_ananassae_flies_Insecta_Drosophilidae_XP_001959097.1
114 Ceratitis_capitata_Mediterranean_fruit_fly_Insecta_Tephritidae_XP_004520779.1
115 Ceratitis_capitata_Mediterranean_fruit_fly_Insecta_Tephritidae_XP_012154689.1
116 Bombus_bifarius_bees_Insecta_Apidae_XP_033306936.1
117 Athalia_rosae_coleseed_sawfly_Insecta_Tenthredinidae_XP_012253605.1
118 Drosophila_busckii_flies_Insecta_Drosophilidae_XP_017835817.1
119 Drosophila_busckii_flies_Insecta_Drosophilidae_XP_017835874.1
120 Drosophila_busckii_flies_Insecta_Drosophilidae_XP_017835980.1
121 Drosophila_busckii_flies_Insecta_Drosophilidae_XP_017835701.1
122 Megalopta_genalis_bees_Insecta_Halictidae_XP_033328793.1
123 Rhagoletis_pomonella_apple_maggot_Insecta_Tephritidae_XP_036331553.1
124 Drosophila_subpulchrella_flies_Insecta_Drosophilidae_XP_037715996.1
125 Nylanderia_fulva_ants_Insecta_Formicidae_XP_029178303.1
126 Bombus_impatiens_common_eastern_bumble_bee_Insecta_Apidae_XP_003494027.1

127 Linepithema_humile_Argentine_ant_Insecta_Formicidae_XP_012224689.1
128 Osmia_lignaria_orchard_mason_bee_Insecta_Megachilidae_XP_034173547.1
129 Belonocnema_treatae_wasps_ants_and_bees_Insecta_Cynipidae_XP_033225167.1
130 Amyelois_transitella_moths_Insecta_Pyralidae_XP_013196676.1
131 Musca_domestica_house_fly_Insecta_Muscidae_XP_005190499.1
132 Bicyclus_anynana_squinting_bush_brown_Insecta_XP_023955130.1
133 Bombus_vosnesenskii_bees_Insecta_Apidae_XP_033359789.1
134 Glossina_fuscipes_tsetse_fly_Insecta_Glossinidae_XP_037895698.1
135 Trachymyrmex_septentrionalis_ants_Insecta_Formicidae_XP_018345181.1
136 Leptinotarsa_decemlineata_Colorado_potato_beetle_Insecta_Chrysomelidae_XP_023017831.1
137 Manduca_sexta_tobacco_hornworm_Insecta_Sphingidae_XP_037302089.1
138 Drosophila_subobscura_flies_Insecta_Drosophilidae_XP_034650722.1
139 Drosophila_subobscura_flies_Insecta_Drosophilidae_XP_034653327.1
140 Stomoxys_calcitrans_stable_fly_Insecta_Muscidae_XP_013104900.1
141 Dendroctonus_ponderosae_mountain_pine_beetle_Insecta_Curculionidae_XP_019770061.1
142 Dendroctonus_ponderosae_mountain_pine_beetle_Insecta_Curculionidae_XP_019763053.1
143 Anopheles_arabiensis_mosquitos_Insecta_Culicidae_XP_040165322.1
144 Wasmannia_auropunctata_little_fire_ant_Insecta_Formicidae_XP_011690259.1
145 Contarinia_nasturtii_swede_midge_Insecta_Cecidomyiidae_XP_031623850.1
146 Harpegnathos_saltator_Jerdon_s_jumping_ant_Insecta_Formicidae_XP_025159290.1
147 Drosophila_elegans_flies_Insecta_Drosophilidae_XP_017132952.1
148 Drosophila_elegans_flies_Insecta_Drosophilidae_XP_017123340.1
149 Drosophila_pseudoobscura_flies_Insecta_Drosophilidae_XP_001360584.3
150 Drosophila_pseudoobscura_flies_Insecta_Drosophilidae_XP_001361118.3
151 Bombus_terrestris_buff_tailed_bumblebee_Insecta_Apidae_XP_003393230.2
152 Hermetia_illucens_flies_Insecta_Stratiomyidae_XP_037912477.1
153 Monomorium_pharaonis_pharaoh_ant_Insecta_Formicidae_XP_012529989.1
154 Drosophila_persimilis_flies_Insecta_Drosophilidae_XP_002016144.1
155 Drosophila_persimilis_flies_Insecta_Drosophilidae_XP_026841811.1
156 Vanessa_tameamea_butterflies_Insecta_XP_026489473.1
157 Aphis_gossypii_cotton_aphid_Insecta_Aphididae_XP_027851435.1
158 Camponotus_floridanus_Florida_carpenter_ant_Insecta_Formicidae_XP_025271044.1
159 Bactrocera_tryoni_Queensland_fruit_fly_Insecta_Tephritidae_XP_039955314.1
160 Bactrocera_tryoni_Queensland_fruit_fly_Insecta_Tephritidae_XP_039955452.1
161 Microplitis_demolitor_wasps_ants_and_bees_Insecta_Braconidae_XP_008559651.1
162 Polistes_canadensis_wasps_ants_and_bees_Insecta_Vespidae_XP_014608220.1
163 Photinus_pyralis_common_eastern_firefly_Insecta_Lampyridae_XP_031335015.1
164 Zerene_cesonia_dogface_butterfly_Insecta_Pieridae_XP_038214884.1
165 Folsomia_candida_springtails_Collembola_Isotomidae_XP_021953796.1
166 Orussus_abietinus_hymenopterans_Insecta_Orussidae_XP_012281082.1
167 Drosophila_obscura_flies_Insecta_Drosophilidae_XP_022229280.1
168 Drosophila_obscura_flies_Insecta_Drosophilidae_XP_022221634.1
169 Drosophila_ficusphila_flies_Insecta_Drosophilidae_XP_017039307.1
170 Drosophila_ficusphila_flies_Insecta_Drosophilidae_XP_017044532.1
171 Solenopsis_invicta_red_fire_ant_Insecta_Formicidae_XP_011164321.1
172 Apis_florea_little_honeybee_Insecta_Apidae_XP_012346685.1
173 Diachasma_alloeum_wasps_ants_and_bees_Insecta_Braconidae_XP_015126712.1
174 Trichoplusia_ni_cabbage_looper_Insecta_XP_026732403.1
175 Nilaparvata_lugens_brown_planthopper_Insecta_Delphacidae_XP_022194630.2
176 Helicoverpa_armigera_cotton_bollworm_Insecta_XP_021194330.1
177 Culex_pipiens_pallens_northern_house_mosquito_Insecta_Culicidae_XP_039442490.1
178 Bactrocera_oleae_olive_fruit_fly_Insecta_Tephritidae_XP_014099458.1
179 Bactrocera_oleae_olive_fruit_fly_Insecta_Tephritidae_XP_014099226.1
180 Drosophila_bipectinata_flies_Insecta_Drosophilidae_XP_017093380.1
181 Drosophila_bipectinata_flies_Insecta_Drosophilidae_XP_017093703.1
182 Bombus_vancouverensis_nearcticus_bees_Insecta_Apidae_XP_033188624.1
183 Mus_musculus_house_mouse_Dipnotetrapodomorpha_Muridae_NP_081905.1
184 Mus_musculus_house_mouse_Dipnotetrapodomorpha_Muridae_NP_079976.1
185 Onthophagus_taurus_beetles_Insecta_Scarabaeidae_XP_022915965.1
186 Onthophagus_taurus_beetles_Insecta_Scarabaeidae_XP_022917761.1
187 Onthophagus_taurus_beetles_Insecta_Scarabaeidae_XP_022915851.1
188 Onthophagus_taurus_beetles_Insecta_Scarabaeidae_XP_022918562.1
189 Myzus_persicae_green_peach_aphid_Insecta_Aphididae_XP_022160614.1

```
		Bemisia_tabaci_sweet_potato_whitefly_Insecta_Aleyrodidae_XP_018907913.1
		Dinoponera_quadriceps_ants_Insecta_Formicidae_XP_014484360.1
		Aethina_tumida_small_hive_beetle_Insecta_XP_019864613.1
		Nasonia_vitripennis_jewel_wasp_Insecta_Pteromalidae_XP_032454843.1
		Cephus_cinctus_wheat_stem_sawfly_Insecta_Cephidae_XP_015586698.1
		Drosophila_kikkawai_flies_Insecta_Drosophilidae_XP_017019697.1
		Drosophila_kikkawai_flies_Insecta_Drosophilidae_XP_017027378.1
		Drosophila_mojavensis_flies_Insecta_Drosophilidae_XP_002004224.1
		Drosophila_mojavensis_flies_Insecta_Drosophilidae_XP_002006547.1
		Drosophila_mojavensis_flies_Insecta_Drosophilidae_XP_002006390.1
		Sipha_flava_yellow_sugarcane_aphid_Insecta_Aphididae_XP_025409349.1
		Trachymyrmex_zeteki_ants_Insecta_Formicidae_XP_018316080.1
		Drosophila_grimshawi_flies_Insecta_Drosophilidae_XP_001995249.1
		Drosophila_grimshawi_flies_Insecta_Drosophilidae_XP_032590626.1
		Drosophila_grimshawi_flies_Insecta_Drosophilidae_XP_001986531.1
		Tribolium_castaneum_red_flour_beetle_Insecta_Tenebrionidae_XP_008197604.1
		Drosophila_santomea_flies_Insecta_Drosophilidae_XP_039483597.1
		Drosophila_santomea_flies_Insecta_Drosophilidae_XP_039480332.1
		Habropoda_laboriosa_bees_Insecta_Apidae_XP_017799187.1
		Polistes_dominula_European_paper_wasp_Insecta_Vespidae_XP_015190318.1
		Papilio_polytes_common_Mormon_Insecta_Papilionidae_XP_013149198.1
		Zeugodacus_cucurbitae_melon_fly_Insecta_Tephritidae_XP_011178738.1
		Zeugodacus_cucurbitae_melon_fly_Insecta_Tephritidae_XP_011178742.1
		Lucilia_sericata_common_green_bottle_fly_Insecta_Calliphoridae_XP_037815336.1
		Bombyx_mandarina_wild_silkworm_Insecta_Bombycidae_XP_028030827.1
		Acromyrmex_echinatior_Panamanian_leafcutter_ant_Insecta_Formicidae_XP_011065291.1
		Drosophila_willistoni_flies_Insecta_Drosophilidae_XP_002074879.1
		Drosophila_willistoni_flies_Insecta_Drosophilidae_XP_002074789.1
		Drosophila_guanche_flies_Insecta_Drosophilidae_XP_034122227.1
		Drosophila_guanche_flies_Insecta_Drosophilidae_XP_034128148.1
		Aedes_albopictus_Asian_tiger_mosquito_Insecta_Culicidae_XP_029712473.1
		Culex_quinquefasciatus_southern_house_mosquito_Insecta_Culicidae_XP_038113782.1
		Odontomachus_brunneus_ants_Insecta_Formicidae_XP_032671569.1
		Drosophila_virilis_flies_Insecta_Drosophilidae_XP_002049062.1
		Drosophila_virilis_flies_Insecta_Drosophilidae_XP_002059384.2
		Drosophila_virilis_flies_Insecta_Drosophilidae_XP_002050082.1
		Pararge_aegeria_specked_wood_butterfly_Insecta_XP_039751581.1
		;
end;
begin trees;
	translate
		1	Drosophila_hydei_flies_Insecta_Drosophilidae_XP_023161462.1,
		2	Drosophila_hydei_flies_Insecta_Drosophilidae_XP_023172448.2,
		3	Drosophila_hydei_flies_Insecta_Drosophilidae_XP_023171551.2,
		4	Diabrotica_virgifera_virgifera_western_corn_rootworm_Insecta_Chrysomelidae_XP_028143025.1,
		5	Drosophila_yakuba_flies_Insecta_Drosophilidae_XP_002091361.1,
		6	Drosophila_yakuba_flies_Insecta_Drosophilidae_XP_002089741.2,
		7	Fopius_arisanus_wasps_ants_and_bees_Insecta_Braconidae_XP_011310787.1,
		8	Teleopsis_dalmanni_flies_Insecta_Diopsidae_XP_037929741.1,
		9	Teleopsis_dalmanni_flies_Insecta_Diopsidae_XP_037949477.1,
		10	Trichogramma_pretiosum_wasps_ants_and_bees_Insecta_Trichogrammatidae_XP_014238796.1,
		11	Pseudomyrmex_gracilis_ants_Insecta_Formicidae_XP_020280661.1,
		12	Cyphomyrmex_costatus_ants_Insecta_Formicidae_XP_018396236.1,
		13	Sitophilus_oryzae_rice_weevil_Insecta_Curculionidae_XP_030757591.1,
		14	Sitophilus_oryzae_rice_weevil_Insecta_Curculionidae_XP_030768391.1,
		15	Apis_dorsata_giant_honeybee_Insecta_Apidae_XP_006623156.1,
		16	Vespa_mandarinia_Asian_giant_hornet_Insecta_Vespidae_XP_035743739.1,
		17	Atta_cephalotes_ants_Insecta_Formicidae_XP_012054188.1,
		18	Drosophila_albomicans_flies_Insecta_Drosophilidae_XP_034108453.1,
		19	Drosophila_albomicans_flies_Insecta_Drosophilidae_XP_034108654.1,
		20	Drosophila_albomicans_flies_Insecta_Drosophilidae_XP_034107122.1,
		21	Maniola_hyperantus_ringlet_Insecta_XP_034827204.1,
		22	Apis_mellifera_honey_bee_Insecta_Apidae_XP_026297163.1,
```

```
		23	Cimex_lectularius_bed_bug_Insecta_Cimicidae_XP_014251917.1,
		24	Drosophila_sechellia_flies_Insecta_Drosophilidae_XP_002033901.1,
		25	Drosophila_sechellia_flies_Insecta_Drosophilidae_XP_032572942.1,
		26	Drosophila_melanogaster_fruit_fly_Insecta_Drosophilidae_NP_610992.2,
		27	Drosophila_melanogaster_fruit_fly_Insecta_Drosophilidae_NP_610453.1,
		28	Vollenhovia_emeryi_ants_Insecta_Formicidae_XP_011871365.1,
		29	Drosophila_novamexicana_flies_Insecta_Drosophilidae_XP_030566323.1,
		30	Drosophila_novamexicana_flies_Insecta_Drosophilidae_XP_030566424.1,
		31	Drosophila_novamexicana_flies_Insecta_Drosophilidae_XP_030559958.1,
		32	Drosophila_novamexicana_flies_Insecta_Drosophilidae_XP_030559753.1,
		33	Spodoptera_frugiperda_fall_armyworm_Insecta_XP_035434313.1,
		34	Spodoptera_frugiperda_fall_armyworm_Insecta_XP_035434146.1,
		35	Scaptodrosophila_lebanonensis_flies_Insecta_Drosophilidae_XP_030387011.1,
		36	Scaptodrosophila_lebanonensis_flies_Insecta_Drosophilidae_XP_030372628.1,
		37	Pieris_rapae_cabbage_white_Insecta_Pieridae_XP_022123915.1,
		38	Atta_colombica_ants_Insecta_Formicidae_XP_018046319.1,
		39	Rhagoletis_zephyria_snowberry_fruit_fly_Insecta_Tephritidae_XP_017469128.1,
		40	Drosophila_navojoa_flies_Insecta_Drosophilidae_XP_017959596.1,
		41	Drosophila_navojoa_flies_Insecta_Drosophilidae_XP_017962256.1,
		42	Drosophila_navojoa_flies_Insecta_Drosophilidae_XP_017962257.1,
		43	Drosophila_navojoa_flies_Insecta_Drosophilidae_XP_017958700.1,
		44	Acyrthosiphon_pisum_pea_aphid_Insecta_Aphididae_XP_008186737.1,
		45	Anopheles_coluzzii_mosquitos_Insecta_Culicidae_XP_040231685.1,
		46	Drosophila_mauritiana_flies_Insecta_Drosophilidae_XP_033155850.1,
		47	Drosophila_mauritiana_flies_Insecta_Drosophilidae_XP_033155919.1,
		48	Megachile_rotundata_alfalfa_leafcutting_bee_Insecta_Megachilidae_XP_003703795.1,
		49	Trachymyrmex_cornetzi_ants_Insecta_Formicidae_XP_018364226.1,
		50	Ctenocephalides_felis_cat_flea_Insecta_Pulicidae_XP_026473532.1,
		51	Anoplophora_glabripennis_Asian_longhorned_beetle_Insecta_Cerambycidae_XP_018564394.1,
		52	Drosophila_serrata_flies_Insecta_Drosophilidae_XP_020801284.1,
		53	Drosophila_serrata_flies_Insecta_Drosophilidae_XP_020814606.1,
		54	Anopheles_albimanus_mosquitos_Insecta_Culicidae_XP_035776653.1,
		55	Apis_cerana_Asiatic_honeybee_Insecta_Apidae_XP_016922536.1,
		56	Ooceraea_biroi_clonal_raider_ant_Insecta_Formicidae_XP_011339242.2,
		57	Hyposmocoma_kahamanoa_moths_Insecta_Cosmopterigidae_XP_026315441.1,
		58	Drosophila_rhopaloa_flies_Insecta_Drosophilidae_XP_016975014.1,
		59	Drosophila_rhopaloa_flies_Insecta_Drosophilidae_XP_016971263.1,
		60	Ceratina_calcarata_bees_Insecta_Apidae_XP_017879794.2,
		61	Melanaphis_sacchari_aphids_Insecta_Aphididae_XP_025205583.1,
		62	Diuraphis_noxia_Russian_wheat_aphid_Insecta_Aphididae_XP_015378800.1,
		63	Nomia_melanderi_Alkali_bee_Insecta_Halictidae_XP_031839243.1,
		64	Drosophila_eugracilis_flies_Insecta_Drosophilidae_XP_017068721.1,
		65	Drosophila_eugracilis_flies_Insecta_Drosophilidae_XP_017066148.1,
		66	Osmia_bicornis_bicornis_red_mason_bee_Insecta_Megachilidae_XP_029048774.1,
		67	Lucilia_cuprina_Australian_sheep_blowfly_Insecta_Calliphoridae_XP_023307068.1,
		68	Drosophila_biarmipes_flies_Insecta_Drosophilidae_XP_016946573.1,
		69	Drosophila_biarmipes_flies_Insecta_Drosophilidae_XP_016968374.1,
		70	Drosophila_erecta_flies_Insecta_Drosophilidae_XP_001975581.1,
		71	Drosophila_erecta_flies_Insecta_Drosophilidae_XP_001970362.2,
		72	Drosophila_simulans_flies_Insecta_Drosophilidae_XP_016027660.1,
		73	Drosophila_simulans_flies_Insecta_Drosophilidae_XP_016026622.1,
		74	Nicrophorus_vespilloides_beetles_Insecta_Silphidae_XP_017781760.1,
		75	Thrips_palmi_thrips_Insecta_Thripidae_XP_034255598.1,
		76	Bactrocera_latifrons_flies_Insecta_Tephritidae_XP_018803639.1,
		77	Bactrocera_latifrons_flies_Insecta_Tephritidae_XP_018803564.1,
		78	Drosophila_suzukii_flies_Insecta_Drosophilidae_XP_016928030.1,
		79	Drosophila_suzukii_flies_Insecta_Drosophilidae_XP_016930474.1,
		80	Bombyx_mori_domestic_silkworm_Insecta_Bombycidae_XP_004929437.1,
		81	Bradysia_coprophila_flies_Insecta_Sciaridae_XP_037047063.1,
		82	Cryptotermes_secundus_termites_Insecta_Kalotermitidae_XP_033606653.1,
		83	Anopheles_stephensi_Asian_malaria_mosquito_Insecta_Culicidae_XP_035905304.1,
		84	
```

```
Copidosoma_floridanum_wasps_ants_and_bees_Insecta_Encyrtidae_XP_014218420.1,
		85	Eufriesea_mexicana_bees_Insecta_Apidae_XP_017765990.1,
		86	Bactrocera_dorsalis_oriental_fruit_fly_Insecta_Tephritidae_XP_011198066.1,
		87	Bactrocera_dorsalis_oriental_fruit_fly_Insecta_Tephritidae_XP_011198068.1,
		88	Homo_sapiens_human_Dipnotetrapodomorpha_Hominidae_NP_060760.2,
		89	Homo_sapiens_human_Dipnotetrapodomorpha_Hominidae_NP_775853.2,
		90	Papilio_machaon_common_yellow_swallowtail_Insecta_Papilionidae_XP_014369384.1,
		91	Pogonomyrmex_barbatus_red_harvester_ant_Insecta_Formicidae_XP_025075767.1,
		92	Drosophila_miranda_flies_Insecta_Drosophilidae_XP_017150747.1,
		93	Drosophila_miranda_flies_Insecta_Drosophilidae_XP_033247062.1,
		94	Drosophila_miranda_flies_Insecta_Drosophilidae_XP_017147529.2,
		95	Drosophila_miranda_flies_Insecta_Drosophilidae_XP_033255654.1,
		96	Pediculus_humanus_corporis_human_body_louse_Insecta_Pediculidae_XP_002428808.1,
		97	Ceratosolen_solmsi_marchali_wasps_ants_and_bees_Insecta_Agaonidae_XP_011502643.1,
		98	Aedes_aegypti_yellow_fever_mosquito_Insecta_Culicidae_XP_001653384.1,
		99	Danaus_plexippus_plexippus_monarch_butterfly_Insecta_XP_032515592.1,
		100	Temnothorax_curvispinosus_ants_Insecta_Formicidae_XP_024878483.1,
		101	Drosophila_takahashii_flies_Insecta_Drosophilidae_XP_016993793.1,
		102	Drosophila_takahashii_flies_Insecta_Drosophilidae_XP_017015161.1,
		103	Chelonus_insularis_wasps_ants_and_bees_Insecta_Braconidae_XP_034944353.1,
		104	Formica_exsecta_ants_Insecta_Formicidae_XP_029678652.1,
		105	Rhopalosiphum_maidis_corn_leaf_aphid_Insecta_Aphididae_XP_026807396.1,
		106	Drosophila_ananassae_flies_Insecta_Drosophilidae_XP_001959966.1,
		107	Drosophila_ananassae_flies_Insecta_Drosophilidae_XP_001959097.1,
		108	Ceratitis_capitata_Mediterranean_fruit_fly_Insecta_Tephritidae_XP_004520779.1,
		109	Ceratitis_capitata_Mediterranean_fruit_fly_Insecta_Tephritidae_XP_012154689.1,
		110	Bombus_bifarius_bees_Insecta_Apidae_XP_033306936.1,
		111	Athalia_rosae_coleseed_sawfly_Insecta_Tenthredinidae_XP_012253605.1,
		112	Drosophila_busckii_flies_Insecta_Drosophilidae_XP_017835817.1,
		113	Drosophila_busckii_flies_Insecta_Drosophilidae_XP_017835874.1,
		114	Drosophila_busckii_flies_Insecta_Drosophilidae_XP_017835980.1,
		115	Drosophila_busckii_flies_Insecta_Drosophilidae_XP_017835701.1,
		116	Megalopta_genalis_bees_Insecta_Halictidae_XP_033328793.1,
		117	Rhagoletis_pomonella_apple_maggot_Insecta_Tephritidae_XP_036331553.1,
		118	Drosophila_subpulchrella_flies_Insecta_Drosophilidae_XP_037715996.1,
		119	Nylanderia_fulva_ants_Insecta_Formicidae_XP_029178303.1,
		120	Bombus_impatiens_common_eastern_bumble_bee_Insecta_Apidae_XP_003494027.1,
		121	Linepithema_humile_Argentine_ant_Insecta_Formicidae_XP_012224689.1,
		122	Osmia_lignaria_orchard_mason_bee_Insecta_Megachilidae_XP_034173547.1,
		123	Belonocnema_treatae_wasps_ants_and_bees_Insecta_Cynipidae_XP_033225167.1,
		124	Amyelois_transitella_moths_Insecta_Pyralidae_XP_013196676.1,
		125	Musca_domestica_house_fly_Insecta_Muscidae_XP_005190499.1,
		126	Bicyclus_anynana_squinting_bush_brown_Insecta_XP_023955130.1,
		127	Bombus_vosnesenskii_bees_Insecta_Apidae_XP_033359789.1,
		128	Glossina_fuscipes_tsetse_fly_Insecta_Glossinidae_XP_037895698.1,
		129	Trachymyrmex_septentrionalis_ants_Insecta_Formicidae_XP_018345181.1,
		130	Leptinotarsa_decemlineata_Colorado_potato_beetle_Insecta_Chrysomelidae_XP_023017831.1,
		131	Manduca_sexta_tobacco_hornworm_Insecta_Sphingidae_XP_037302089.1,
		132	Drosophila_subobscura_flies_Insecta_Drosophilidae_XP_034650722.1,
		133	Drosophila_subobscura_flies_Insecta_Drosophilidae_XP_034653327.1,
		134	Stomoxys_calcitrans_stable_fly_Insecta_Muscidae_XP_013104900.1,
		135	Dendroctonus_ponderosae_mountain_pine_beetle_Insecta_Curculionidae_XP_019770061.1,
		136	
```

```
		Dendroctonus_ponderosae_mountain_pine_beetle_Insecta_Curculionidae_XP_019763053.1,
		137	Anopheles_arabiensis_mosquitos_Insecta_Culicidae_XP_040165322.1,
		138	Wasmannia_auropunctata_little_fire_ant_Insecta_Formicidae_XP_011690259.1,
		139	Contarinia_nasturtii_swede_midge_Insecta_Cecidomyiidae_XP_031623850.1,
		140	Harpegnathos_saltator_Jerdon_s_jumping_ant_Insecta_Formicidae_XP_025159290.1,
		141	Drosophila_elegans_flies_Insecta_Drosophilidae_XP_017132952.1,
		142	Drosophila_elegans_flies_Insecta_Drosophilidae_XP_017123340.1,
		143	Drosophila_pseudoobscura_flies_Insecta_Drosophilidae_XP_001360584.3,
		144	Drosophila_pseudoobscura_flies_Insecta_Drosophilidae_XP_001361118.3,
		145	Bombus_terrestris_buff_tailed_bumblebee_Insecta_Apidae_XP_003393230.2,
		146	Hermetia_illucens_flies_Insecta_Stratiomyidae_XP_037912477.1,
		147	Monomorium_pharaonis_pharaoh_ant_Insecta_Formicidae_XP_012529989.1,
		148	Drosophila_persimilis_flies_Insecta_Drosophilidae_XP_002016144.1,
		149	Drosophila_persimilis_flies_Insecta_Drosophilidae_XP_026841811.1,
		150	Vanessa_tameamea_butterflies_Insecta_XP_026489473.1,
		151	Aphis_gossypii_cotton_aphid_Insecta_Aphididae_XP_027851435.1,
		152	Camponotus_floridanus_Florida_carpenter_ant_Insecta_Formicidae_XP_025271044.1,
		153	Bactrocera_tryoni_Queensland_fruit_fly_Insecta_Tephritidae_XP_039955314.1,
		154	Bactrocera_tryoni_Queensland_fruit_fly_Insecta_Tephritidae_XP_039955452.1,
		155	Microplitis_demolitor_wasps_ants_and_bees_Insecta_Braconidae_XP_008559651.1,
		156	Polistes_canadensis_wasps_ants_and_bees_Insecta_Vespidae_XP_014608220.1,
		157	Photinus_pyralis_common_eastern_firefly_Insecta_Lampyridae_XP_031335015.1,
		158	Zerene_cesonia_dogface_butterfly_Insecta_Pieridae_XP_038214884.1,
		159	Folsomia_candida_springtails_Collembola_Isotomidae_XP_021953796.1,
		160	Orussus_abietinus_hymenopterans_Insecta_Orussidae_XP_012281082.1,
		161	Drosophila_obscura_flies_Insecta_Drosophilidae_XP_022229280.1,
		162	Drosophila_obscura_flies_Insecta_Drosophilidae_XP_022221634.1,
		163	Drosophila_ficusphila_flies_Insecta_Drosophilidae_XP_017039307.1,
		164	Drosophila_ficusphila_flies_Insecta_Drosophilidae_XP_017044532.1,
		165	Solenopsis_invicta_red_fire_ant_Insecta_Formicidae_XP_011164321.1,
		166	Apis_florea_little_honeybee_Insecta_Apidae_XP_012346685.1,
		167	Diachasma_alloeum_wasps_ants_and_bees_Insecta_Braconidae_XP_015126712.1,
		168	Trichoplusia_ni_cabbage_looper_Insecta_XP_026732403.1,
		169	Nilaparvata_lugens_brown_planthopper_Insecta_Delphacidae_XP_022194630.2,
		170	Helicoverpa_armigera_cotton_bollworm_Insecta_XP_021194330.1,
		171	Culex_pipiens_pallens_northern_house_mosquito_Insecta_Culicidae_XP_039442490.1,
		172	Bactrocera_oleae_olive_fruit_fly_Insecta_Tephritidae_XP_014099458.1,
		173	Bactrocera_oleae_olive_fruit_fly_Insecta_Tephritidae_XP_014099226.1,
		174	Drosophila_bipectinata_flies_Insecta_Drosophilidae_XP_017093380.1,
		175	Drosophila_bipectinata_flies_Insecta_Drosophilidae_XP_017093703.1,
		176	Bombus_vancouverensis_nearcticus_bees_Insecta_Apidae_XP_033188624.1,
		177	Mus_musculus_house_mouse_Dipnotetrapodomorpha_Muridae_NP_081905.1,
		178	Mus_musculus_house_mouse_Dipnotetrapodomorpha_Muridae_NP_079976.1,
		179	Onthophagus_taurus_beetles_Insecta_Scarabaeidae_XP_022915965.1,
		180	Onthophagus_taurus_beetles_Insecta_Scarabaeidae_XP_022917761.1,
		181	Onthophagus_taurus_beetles_Insecta_Scarabaeidae_XP_022915851.1,
		182	Onthophagus_taurus_beetles_Insecta_Scarabaeidae_XP_022918562.1,
		183	Myzus_persicae_green_peach_aphid_Insecta_Aphididae_XP_022160614.1,
		184	Bemisia_tabaci_sweet_potato_whitefly_Insecta_Aleyrodidae_XP_018907913.1,
		185	Dinoponera_quadriceps_ants_Insecta_Formicidae_XP_014484360.1,
		186	Aethina_tumida_small_hive_beetle_Insecta_XP_019864613.1,
		187	Nasonia_vitripennis_jewel_wasp_Insecta_Pteromalidae_XP_032454843.1,
		188	Cephus_cinctus_wheat_stem_sawfly_Insecta_Cephidae_XP_015586698.1,
		189	Drosophila_kikkawai_flies_Insecta_Drosophilidae_XP_017019697.1,
		190	Drosophila_kikkawai_flies_Insecta_Drosophilidae_XP_017027378.1,
		191	Drosophila_mojavensis_flies_Insecta_Drosophilidae_XP_002004224.1,
		192	Drosophila_mojavensis_flies_Insecta_Drosophilidae_XP_002006547.1,
		193	Drosophila_mojavensis_flies_Insecta_Drosophilidae_XP_002006390.1,
		194	Sipha_flava_yellow_sugarcane_aphid_Insecta_Aphididae_XP_025409349.1,
		195	Trachymyrmex_zeteki_ants_Insecta_Formicidae_XP_018316080.1,
		196	Drosophila_grimshawi_flies_Insecta_Drosophilidae_XP_001995249.1,
```

```
		197	Drosophila_grimshawi_flies_Insecta_Drosophilidae_XP_032590626.1,
		198	Drosophila_grimshawi_flies_Insecta_Drosophilidae_XP_001986531.1,
		199	Tribolium_castaneum_red_flour_beetle_Insecta_Tenebrionidae_XP_008197604.1,
		200	Drosophila_santomea_flies_Insecta_Drosophilidae_XP_039483597.1,
		201	Drosophila_santomea_flies_Insecta_Drosophilidae_XP_039480332.1,
		202	Habropoda_laboriosa_bees_Insecta_Apidae_XP_017799187.1,
		203	Polistes_dominula_European_paper_wasp_Insecta_Vespidae_XP_015190318.1,
		204	Papilio_polytes_common_Mormon_Insecta_Papilionidae_XP_013149198.1,
		205	Zeugodacus_cucurbitae_melon_fly_Insecta_Tephritidae_XP_011178738.1,
		206	Zeugodacus_cucurbitae_melon_fly_Insecta_Tephritidae_XP_011178742.1,
		207	Lucilia_sericata_common_green_bottle_fly_Insecta_Calliphoridae_XP_037815336.1,
		208	Bombyx_mandarina_wild_silkworm_Insecta_Bombycidae_XP_028030827.1,
		209	Acromyrmex_echinatior_Panamanian_leafcutter_ant_Insecta_Formicidae_XP_011065291.1,
		210	Drosophila_willistoni_flies_Insecta_Drosophilidae_XP_002074879.1,
		211	Drosophila_willistoni_flies_Insecta_Drosophilidae_XP_002074789.1,
		212	Drosophila_guanche_flies_Insecta_Drosophilidae_XP_034122227.1,
		213	Drosophila_guanche_flies_Insecta_Drosophilidae_XP_034128148.1,
		214	Aedes_albopictus_Asian_tiger_mosquito_Insecta_Culicidae_XP_029712473.1,
		215	Culex_quinquefasciatus_southern_house_mosquito_Insecta_Culicidae_XP_038113782.1,
		216	Odontomachus_brunneus_ants_Insecta_Formicidae_XP_032671569.1,
		217	Drosophila_virilis_flies_Insecta_Drosophilidae_XP_002049062.1,
		218	Drosophila_virilis_flies_Insecta_Drosophilidae_XP_002059384.2,
		219	Drosophila_virilis_flies_Insecta_Drosophilidae_XP_002050082.1,
		220	Pararge_aegeria_specked_wood_butterfly_Insecta_XP_039751581.1
		;
   [Note: This tree contains information on the topology, 
          branch lengths (if present), and the probability
          of the partition indicated by the branch.]
   tree con_50_majrule = 
   (1:0.1744831,(41:0.02885967,192:0.02963361)1.000:0.09429064,(((((((((((((2:0.1275
   499,(43:0.04051201,193:0.03965172)1.000:0.1040754)1.000:0.1303643,(32:0.01617793,21
   9:0.02934486)1.000:0.1458753)1.000:0.04251596,198:0.2935841)1.000:0.06369057,19:0.3
   059531)1.000:0.04584146,114:0.2664585)1.000:0.07509881,((((((((((5:0.01363528,200:
   0.01208881)1.000:0.0308877,70:0.03788261)0.534:0.01131066,(((24:0.01745503,46:0.017
   0071)0.886:0.003878185,72:0.01628139)1.000:0.01010666,26:0.03037318)1.000:0.0426211
   3)1.000:0.05790253,64:0.176032)1.000:0.02459944,((68:0.04525526,78:0.05603386)1.000
   :0.03015675,101:0.08305551)1.000:0.02505486)1.000:0.02183769,(58:0.06414486,141:0.0
   8824343)1.000:0.06021656)1.000:0.02960671,163:0.1320668)1.000:0.08269226,(52:0.1104
   447,189:0.1036091)1.000:0.152282)1.000:0.05568434,(106:0.07896387,174:0.07312349)1.
   000:0.1572645)1.000:0.1383626,(((92:0.01085767,95:0.02333509)0.606:0.002466972,(143
   :0.004656222,148:0.004726118)1.000:0.004711245)1.000:0.04301997,((132:0.01651067,21
   2:0.02439144)1.000:0.04629167,161:0.04339905)1.000:0.03427015)1.000:0.1468524)1.000
   :0.07327633,(35:0.4424355,210:0.3181532)0.976:0.05943253)1.000:0.03957419)1.000:0.2
   366193,((((((((4:0.4820578,(((13:0.02168493,14:0.04035016)1.000:0.3955375,135:0.38
   20352)1.000:0.2079288,(51:0.3682072,130:0.2980572)0.998:0.07692972)0.747:0.02704313
   )0.851:0.06076458,136:0.7552717)0.867:0.0510328,(186:0.4437667,199:0.4828916)0.751:
   0.07698804)1.000:0.09801476,(((179:0.2592476,181:0.326391)1.000:0.6419281,182:1.383
   053)1.000:0.2861959,180:0.5185474)1.000:0.1410647)0.717:0.05101878,(74:0.685857,157
   :0.5436733)0.999:0.119793)1.000:0.1030391,((((((((7:0.1399617,167:0.1224578)1.000:0
   .3492936,103:0.260433)0.996:0.06799021,155:0.4211523)0.819:0.05462677,(((((10:0.308
   999,187:0.1641021)0.991:0.04823485,84:0.2224453)0.902:0.03850317,97:0.1560199)1.000
   :0.1490143,((((((((11:0.1301955,121:0.1164845)0.773:0.02347671,((((12:0.0410138,(((
   ((17:0.004043905,38:0.004251309)1.000:0.0203286,209:0.02098466)0.835:0.002447993,12
   9:0.01603892)0.985:0.003193111,49:0.01839521)1.000:0.009422772,195:0.02582926)1.000
   :0.01789123)1.000:0.03465324,(((28:0.1025043,138:0.1121843)1.000:0.03782408,100:0.0
   4922008)1.000:0.04383984,(147:0.06953862,165:0.09146174)1.000:0.03070399)0.996:0.01
   818681)1.000:0.04157672,91:0.09620079)1.000:0.03957805,56:0.191893)0.561:0.01362939
   )0.583:0.01464534,((104:0.05613516,119:0.05780215)0.597:0.01362165,152:0.05409531)1
   .000:0.08062149)0.773:0.03464231,((140:0.08626017,216:0.06744682)0.918:0.01348407,1
   85:0.08813132)1.000:0.09156218)1.000:0.16247,((((((((15:0.007225116,(22:0.01091224,
   55:0.006014781)1.000:0.005881551)0.722:0.004933854,166:0.01653142)1.000:0.07767093,
   (85:0.07961611,(((110:4.404135E-4,176:0.001119915)0.988:0.001123197,120:0.001750238
   ,127:5.324603E-4)1.000:0.007607151,145:0.007353317)1.000:0.05736548)1.000:0.0164480
   1)1.000:0.02399169,202:0.1095886)0.998:0.02129074,60:0.1879173)0.844:0.01961747,(48
   :0.09462031,(66:0.008963943,122:0.005317481)1.000:0.06905904)1.000:0.05956858)1.000
   :0.03652781,(63:0.09811924,116:0.1407467)1.000:0.07444014)1.000:0.1326901,(16:0.071
```

```
14237,(156:0.02326644,203:0.02370664)1.000:0.0646462)1.000:0.1289558)0.978:0.034797
15)1.000:0.0668312,160:0.4141984)0.811:0.0311972,188:0.215342)0.828:0.03697246,123:
0.3913478)0.979:0.0328902)0.995:0.0480529,111:0.3880317)0.548:0.04725385)1.000:0.21
53187,((((44:0.04289627,(62:0.05020679,183:0.03350379)0.998:0.007301952)1.000:0.018
17503,((61:0.08524068,105:0.04143731)0.603:0.007186316,151:0.06518432)1.000:0.03000
932)1.000:0.05761743,194:0.1501854)1.000:0.6762435,184:0.5851591)0.977:0.07434308)0
.551:0.04344606,((23:0.4937832,169:0.6860041)0.847:0.09101352,96:0.5514782)0.687:0.
07779296)0.677:0.0589209,(75:0.5711268,82:0.5557618)0.990:0.09611347)0.691:0.048734
03,(((88:0.06320169,178:0.1502558)1.000:0.3842339,(89:0.07060134,177:0.09661355)1.0
00:0.4476838)1.000:0.32957,159:0.8995281)0.689:0.08893558)0.693:0.04238503,(((((((2
1:0.1131719,220:0.17296)1.000:0.04473814,126:0.2289835)1.000:0.0746324,(99:0.339623
1,150:0.3219775)1.000:0.06575839)1.000:0.05096389,(37:0.3162499,158:0.3365299)1.000
:0.1429604)1.000:0.05481142,(90:0.1181557,204:0.1174874)1.000:0.1902755)1.000:0.036
80331,(((((33:0.01256084,34:0.0161254)1.000:0.151691,170:0.1485585)0.992:0.04510268
,168:0.1893013)1.000:0.1552264,124:0.3158595)0.921:0.02878326,((80:0.008730888,208:
0.01162361)1.000:0.2642301,131:0.2612098)1.000:0.07184068)0.959:0.02111077)1.000:0.
1436443,57:0.2345263)1.000:0.8661897)0.955:0.06525126,50:0.7775112)1.000:0.1369439,
(((39:0.007932897,117:0.003776224)1.000:0.1541755,(((((77:0.03047582,154:0.02370475
)0.659:0.00311436,86:0.01935213)1.000:0.03676609,173:0.04770268)1.000:0.06083781,20
6:0.07588643)1.000:0.08668135,109:0.1672688)1.000:0.0903744)1.000:0.1458719,((((76:
0.01936188,(87:0.02184805,153:0.02156148)1.000:0.005826856)1.000:0.02049161,172:0.0
2930556)1.000:0.06524156,205:0.06145953)1.000:0.1338047,108:0.2386888)1.000:0.26108
43)1.000:0.2753726,(((((((45:0.001688205,137:0.01094472)1.000:0.07192866,83:0.06963
993)1.000:0.06775603,54:0.14862)1.000:0.1520877,((98:0.1003764,214:0.08878961)1.000
:0.1776134,(171:0.008518043,215:0.01672738)1.000:0.2065449)1.000:0.07737015)1.000:0
.3116207,146:0.4748701)0.841:0.08334562,81:0.3904005)0.982:0.08315362,139:0.4494385
)1.000:0.1521324)0.505:0.07395648,((8:0.001646243,9:0.001755909)1.000:0.397556,(((6
7:0.03305219,207:0.04784218)1.000:0.1756985,(125:0.1475599,134:0.220834)1.000:0.098
34258)1.000:0.06615076,128:0.3522389)1.000:0.1311579)0.992:0.09170363)1.000:0.35145
64)1.000:0.1502237,36:0.3875563)0.812:0.03618777,((((((((((6:0.00597047,201:0.00579
2581)1.000:0.02960458,71:0.03285196)1.000:0.02074376,(((25:0.003500832,73:0.0016020
19)0.874:0.001012237,47:0.007243906)1.000:0.007651957,27:0.02298426)1.000:0.0213000
8)1.000:0.07322828,65:0.1341465)0.886:0.01857228,((59:0.06816095,142:0.03907227)1.0
00:0.02728471,((69:0.03290132,(79:0.0151558,118:0.02056697)1.000:0.02523132)1.000:0
.01537086,102:0.04940122)1.000:0.01669203)0.950:0.00578486)0.730:0.009732814,164:0.
1189936)1.000:0.02958844,(53:0.1479091,190:0.06434096)1.000:0.09256786)1.000:0.0387
6367,(107:0.1007311,175:0.05065762)1.000:0.1456288)1.000:0.08004578,(((93:0.0070643
64,94:0.01894696)1.000:0.003417507,(144:0.007588387,149:0.003341851)1.000:0.0035300
63)1.000:0.07372604,((133:0.01511686,213:0.01985135)1.000:0.03834109,162:0.04831271
)1.000:0.01730019)1.000:0.1129734)1.000:0.0711546,211:0.2925406)0.995:0.05778694)0.
966:0.0497135,(112:0.5099736,113:0.1701668)0.967:0.09388272)0.891:0.03675443,(((3:0
.1242716,(40:0.0404802,191:0.03164352)1.000:0.1733363)1.000:0.1937545,(31:0.0189315
7,218:0.02499551)1.000:0.1400593)1.000:0.05222935,((20:0.2819122,197:0.1645075)0.88
3:0.03640027,115:0.3505555)0.873:0.02271546)1.000:0.09450312)1.000:0.09665358,(29:0
.1528616,42:0.434207)1.000:0.1567268)0.884:0.03003949,18:0.3209727)0.881:0.03022297
,196:0.2334281)1.000:0.04067269,(30:0.01998016,217:0.01942743)1.000:0.1475426)1.000
:0.135065);LF
end;LF
```
