# Supplementary material for: Inferences on the evolution of the ascorbic acid synthesis pathway in insects using Phylogenetic Tree Collapser (PTC), a tool for the automated collapsing of phylogenetic trees using taxonomic information
Source: J Integr Bioinform. 2024 Jul 24;21(2):20230051. doi: 10.1515/jib-2023-0051 (PMC11377030; doi:10.1515/jib-2023-0051)
Supplement: Supplementary file 1 — Supplementary Material Details [file j_jib-2023-0051_suppl_001.zip › Supplementary_File_8_UGDH_Pri.con_PDF.pdf]

```
#NEXUS

[ID: 8037455178]
begin taxa;
	dimensions ntax=137;
	taxlabels
		Drosophila_hydei_flies_Insecta_Drosophilidae_XP_023173306.1
		Diabrotica_virgifera_virgifera_western_corn_rootworm_Insecta_Chrysomelidae_XP_028140132.1
		Diabrotica_virgifera_virgifera_western_corn_rootworm_Insecta_Chrysomelidae_XP_028140130.1
		Fopius_arisanus_wasps_ants_and_bees_Insecta_Braconidae_XP_011311215.1
		Teleopsis_dalmanni_flies_Insecta_Diopsidae_XP_037934081.1
		Pseudomyrmex_gracilis_ants_Insecta_Formicidae_XP_020290099.1
		Cyphomyrmex_costatus_ants_Insecta_Formicidae_XP_018405722.1
		Sitophilus_oryzae_rice_weevil_Insecta_Curculionidae_XP_030749317.1
		Atta_cephalotes_ants_Insecta_Formicidae_XP_012063955.1
		Vespa_mandarinia_Asian_giant_hornet_Insecta_Vespidae_XP_035734227.1
		Drosophila_albomicans_flies_Insecta_Drosophilidae_XP_034109395.1
		Maniola_hyperantus_ringlet_Insecta_XP_034838146.1
		Papilio_xuthus_Asian_swallowtail_Insecta_Papilionidae_XP_013174460.1
		Cimex_lectularius_bed_bug_Insecta_Cimicidae_XP_014243704.1
		Apis_mellifera_honey_bee_Insecta_Apidae_XP_006570997.1
		Vollenhovia_emeryi_ants_Insecta_Formicidae_XP_011877361.1
		Drosophila_melanogaster_fruit_fly_Insecta_Drosophilidae_NP_476980.1
		Drosophila_novamexicana_flies_Insecta_Drosophilidae_XP_030572429.1
		Spodoptera_frugiperda_fall_armyworm_Insecta_XP_035454154.1
		Spodoptera_frugiperda_fall_armyworm_Insecta_XP_035454396.1
		Scaptodrosophila_lebanonensis_flies_Insecta_Drosophilidae_XP_030372461.1
		Pieris_rapae_cabbage_white_Insecta_Pieridae_XP_022119091.1
		Atta_colombica_ants_Insecta_Formicidae_XP_018058633.1
		Rhagoletis_zephyria_snowberry_fruit_fly_Insecta_Tephritidae_XP_017470597.1
		Drosophila_navojoa_flies_Insecta_Drosophilidae_XP_017955051.1
		Acyrthosiphon_pisum_pea_aphid_Insecta_Aphididae_XP_008181512.1
		Megachile_rotundata_alfalfa_leafcutting_bee_Insecta_Megachilidae_XP_012137041.1
		Trachymyrmex_cornetzi_ants_Insecta_Formicidae_XP_018374266.1
		Agrilus_planipennis_emerald_ash_borer_Insecta_Buprestidae_XP_018322526.1
		Ctenocephalides_felis_cat_flea_Insecta_Pulicidae_XP_026472782.1
		Anoplophora_glabripennis_Asian_longhorned_beetle_Insecta_Cerambycidae_XP_018576732.1
		Anoplophora_glabripennis_Asian_longhorned_beetle_Insecta_Cerambycidae_XP_018576728.1
		Drosophila_serrata_flies_Insecta_Drosophilidae_XP_020801661.1
		Ooceraea_biroi_clonal_raider_ant_Insecta_Formicidae_XP_011352179.1
		Apis_cerana_Asiatic_honeybee_Insecta_Apidae_XP_016915229.1
		Hyposmocoma_kahamanoa_moths_Insecta_Cosmopterigidae_XP_026331265.1
		Drosophila_rhopaloa_flies_Insecta_Drosophilidae_XP_016990292.1
		Ceratina_calcarata_bees_Insecta_Apidae_XP_026675585.1
		Melanaphis_sacchari_aphids_Insecta_Aphididae_XP_025205984.1
		Nomia_melanderi_Alkali_bee_Insecta_Halictidae_XP_031839061.1
		Drosophila_eugracilis_flies_Insecta_Drosophilidae_XP_017080359.1
		Osmia_bicornis_bicornis_red_mason_bee_Insecta_Megachilidae_XP_029053011.1
		Lucilia_cuprina_Australian_sheep_blowfly_Insecta_Calliphoridae_XP_023303769.1
		Nicrophorus_vespilloides_beetles_Insecta_Silphidae_XP_017779772.1
		Nicrophorus_vespilloides_beetles_Insecta_Silphidae_XP_017776178.1
		Thrips_palmi_thrips_Insecta_Thripidae_XP_034233444.1
		Bactrocera_latifrons_flies_Insecta_Tephritidae_XP_018795465.1
		Bombyx_mori_domestic_silkworm_Insecta_Bombycidae_XP_004925143.1
		Bradysia_coprophila_flies_Insecta_Sciaridae_XP_037046853.1
		Zootermopsis_nevadensis_termites_Insecta_Termopsidae_XP_021938995.1
		Eufriesea_mexicana_bees_Insecta_Apidae_XP_017764146.1
		Bactrocera_dorsalis_oriental_fruit_fly_Insecta_Tephritidae_XP_011213805.1
		Homo_sapiens_human_Dipnotetrapodomorpha_Hominidae_XP_005262724.1
		Papilio_machaon_common_yellow_swallowtail_Insecta_Papilionidae_XP_014371186.1
```

Drosophila_miranda_flies_Insecta_Drosophilidae_XP_017135620.1
Ceratosolen_solmsi_marchali_wasps_ants_and_bees_Insecta_Agaonidae_XP_011504463.1
Temnothorax_curvispinosus_ants_Insecta_Formicidae_XP_024867521.1
Danaus_plexippus_plexippus_monarch_butterfly_Insecta_XP_032525972.1
Aedes_aegypti_yellow_fever_mosquito_Insecta_Culicidae_XP_001661518.1
Formica_exsecta_ants_Insecta_Formicidae_XP_029667472.1
Rhopalosiphum_maidis_corn_leaf_aphid_Insecta_Aphididae_XP_026816943.1
Ceratitis_capitata_Mediterranean_fruit_fly_Insecta_Tephritidae_XP_004525504.1
Drosophila_ananassae_flies_Insecta_Drosophilidae_XP_001958363.1
Bombus_bifarius_bees_Insecta_Apidae_XP_033318703.1
Athalia_rosae_coleseed_sawfly_Insecta_Tenthredinidae_XP_020708945.1
Drosophila_busckii_flies_Insecta_Drosophilidae_XP_017842574.1
Megalopta_genalis_bees_Insecta_Halictidae_XP_033329072.1
Nylanderia_fulva_ants_Insecta_Formicidae_XP_029164970.1
Bombus_impatiens_common_eastern_bumble_bee_Insecta_Apidae_XP_003485726.1
Linepithema_humile_Argentine_ant_Insecta_Formicidae_XP_012229075.1
Osmia_lignaria_orchard_mason_bee_Insecta_Megachilidae_XP_034195581.1
Belonocnema_treatae_wasps_ants_and_bees_Insecta_Cynipidae_XP_033231214.1
Amyelois_transitella_moths_Insecta_Pyralidae_XP_013195261.1
Bicyclus_anynana_squinting_bush_brown_Insecta_XP_023952108.1
Musca_domestica_house_fly_Insecta_Muscidae_XP_005191114.1
Bombus_vosnesenskii_bees_Insecta_Apidae_XP_033364229.1
Galleria_mellonella_greater_wax_moth_Insecta_Pyralidae_XP_026763549.1
Dufourea_novaeangliae_bees_Insecta_Halictidae_XP_015431079.1
Glossina_fuscipes_tsetse_fly_Insecta_Glossinidae_XP_037880144.1
Trachymyrmex_septentrionalis_ants_XP_018340687.1
Manduca_sexta_tobacco_hornworm_Insecta_Sphingidae_XP_030037441.2
Stomoxys_calcitrans_stable_fly_Insecta_Muscidae_XP_013114599.1
Dendroctonus_ponderosae_mountain_pine_beetle_Insecta_Curculionidae_XP_019766993.1
Wasmannia_auropunctata_little_fire_ant_Insecta_Formicidae_XP_011692078.1
Harpegnathos_saltator_Jerdon_s_jumping_ant_Insecta_Formicidae_XP_011153357.1
Contarinia_nasturtii_swede_midge_Insecta_Cecidomyiidae_XP_031631394.1
Drosophila_pseudoobscura_flies_XP_001354183.1
Bombus_terrestris_buff_tailed_bumblebee_Insecta_Apidae_XP_003402307.1
Monomorium_pharaonis_pharaoh_ant_Insecta_Formicidae_XP_036139285.1
Hermetia_illucens_flies_Insecta_Stratiomyidae_XP_037907628.1
Drosophila_persimilis_flies_Insecta_Drosophilidae_XP_026849516.1
Vanessa_tameamea_butterflies_Insecta_XP_026496444.1
Aphis_gossypii_cotton_aphid_Insecta_Aphididae_XP_027852657.1
Camponotus_floridanus_Florida_carpenter_ant_Insecta_Formicidae_XP_011264013.1
Bactrocera_tryoni_Queensland_fruit_fly_Insecta_Tephritidae_XP_039965029.1
Microplitis_demolitor_wasps_ants_and_bees_Insecta_Braconidae_XP_014300444.1
Polistes_canadensis_wasps_ants_and_bees_Insecta_Vespidae_XP_014616649.1
Photinus_pyralis_common_eastern_firefly_XP_031347802.1
Zerene_cesonia_dogface_butterfly_Insecta_Pieridae_XP_038220977.1
Orussus_abietinus_hymenopterans_Insecta_Orussidae_XP_012282071.1
Folsomia_candida_springtails_Collembola_Isotomidae_XP_035702540.1
Solenopsis_invicta_red_fire_ant_Insecta_Formicidae_XP_011156104.1
Apis_florea_little_honeybee_Insecta_Apidae_XP_012340198.1
Diachasma_alloeum_wasps_ants_and_bees_Insecta_Braconidae_XP_015126514.1
Nilaparvata_lugens_brown_planthopper_Insecta_Delphacidae_XP_039287508.1
Nilaparvata_lugens_brown_planthopper_Insecta_Delphacidae_XP_039287723.1
Trichoplusia_ni_cabbage_looper_Insecta_XP_026746668.1
Helicoverpa_armigera_cotton_bollworm_Insecta_XP_021199365.1
Bactrocera_oleae_olive_fruit_fly_Insecta_Tephritidae_XP_014094726.1
Mus_musculus_house_mouse_Dipnotetrapodomorpha_Muridae_XP_006503925.1
Onthophagus_taurus_beetles_Insecta_Scarabaeidae_XP_022918072.1
Myzus_persicae_green_peach_aphid_Insecta_Aphididae_XP_022182046.1
Dinoponera_quadriceps_ants_Insecta_Formicidae_XP_014471283.1
Aethina_tumida_small_hive_beetle_Insecta_XP_019878451.1
Bemisia_tabaci_sweet_potato_whitefly_Insecta_Aleyrodidae_XP_018908293.1
Nasonia_vitripennis_jewel_wasp_Insecta_Pteromalidae_XP_001599700.1
Cephus_cinctus_wheat_stem_sawfly_Insecta_Cephidae_XP_015594969.1
Drosophila_mojavensis_flies_Insecta_Drosophilidae_XP_002007480.1

```
		Sipha_flava_yellow_sugarcane_aphid_Insecta_Aphididae_XP_025422359.1
		Trachymyrmex_zeteki_ants_Insecta_Formicidae_XP_018309379.1
		Drosophila_grimshawi_flies_Insecta_Drosophilidae_XP_001983810.1
		Tribolium_castaneum_red_flour_beetle_Insecta_Tenebrionidae_XP_008190526.1
		Habropoda_laboriosa_bees_Insecta_Apidae_XP_017797480.1
		Polistes_dominula_European_paper_wasp_Insecta_Vespidae_XP_015185360.1
		Papilio_polytes_common_Mormon_Insecta_Papilionidae_XP_013135877.1
		Zeugodacus_cucurbitae_melon_fly_Insecta_Tephritidae_XP_011196038.1
		Lucilia_sericata_common_green_bottle_fly_Insecta_Calliphoridae_XP_037819406.1
		Drosophila_arizonae_flies_Insecta_Drosophilidae_XP_017862575.1
		Bombyx_mandarina_wild_silkworm_Insecta_Bombycidae_XP_028037540.1
		Acromyrmex_echinatior_Panamanian_leafcutter_ant_Insecta_Formicidae_XP_011060165.1
		Drosophila_willistoni_flies_Insecta_Drosophilidae_XP_002061809.1
		Aedes_albopictus_Asian_tiger_mosquito_Insecta_Culicidae_XP_029718184.1
		Aedes_albopictus_Asian_tiger_mosquito_Insecta_Culicidae_XP_029735770.1
		Aedes_albopictus_Asian_tiger_mosquito_Insecta_Culicidae_XP_029735681.1
		Odontomachus_brunneus_ants_Insecta_Formicidae_XP_032666296.1
		Pararge_aegeria_specked_wood_butterfly_Insecta_XP_039762194.1
		Drosophila_virilis_flies_Insecta_Drosophilidae_XP_002046857.1
		;
end;
begin trees;
	translate
		1	Drosophila_hydei_flies_Insecta_Drosophilidae_XP_023173306.1,
		2	Diabrotica_virgifera_virgifera_western_corn_rootworm_Insecta_Chrysomelidae_XP_028140132.1,
		3	Diabrotica_virgifera_virgifera_western_corn_rootworm_Insecta_Chrysomelidae_XP_028140130.1,
		4	Fopius_arisanus_wasps_ants_and_bees_Insecta_Braconidae_XP_011311215.1,
		5	Teleopsis_dalmanni_flies_Insecta_Diopsidae_XP_037934081.1,
		6	Pseudomyrmex_gracilis_ants_Insecta_Formicidae_XP_020290099.1,
		7	Cyphomyrmex_costatus_ants_Insecta_Formicidae_XP_018405722.1,
		8	Sitophilus_oryzae_rice_weevil_Insecta_Curculionidae_XP_030749317.1,
		9	Atta_cephalotes_ants_Insecta_Formicidae_XP_012063955.1,
		10	Vespa_mandarinia_Asian_giant_hornet_Insecta_Vespidae_XP_035734227.1,
		11	Drosophila_albomicans_flies_Insecta_Drosophilidae_XP_034109395.1,
		12	Maniola_hyperantus_ringlet_Insecta_XP_034838146.1,
		13	Papilio_xuthus_Asian_swallowtail_Insecta_Papilionidae_XP_013174460.1,
		14	Cimex_lectularius_bed_bug_Insecta_Cimicidae_XP_014243704.1,
		15	Apis_mellifera_honey_bee_Insecta_Apidae_XP_006570997.1,
		16	Vollenhovia_emeryi_ants_Insecta_Formicidae_XP_011877361.1,
		17	Drosophila_melanogaster_fruit_fly_Insecta_Drosophilidae_NP_476980.1,
		18	Drosophila_novamexicana_flies_Insecta_Drosophilidae_XP_030572429.1,
		19	Spodoptera_frugiperda_fall_armyworm_Insecta_XP_035454154.1,
		20	Spodoptera_frugiperda_fall_armyworm_Insecta_XP_035454396.1,
		21	Scaptodrosophila_lebanonensis_flies_Insecta_Drosophilidae_XP_030372461.1,
		22	Pieris_rapae_cabbage_white_Insecta_Pieridae_XP_022119091.1,
		23	Atta_colombica_ants_Insecta_Formicidae_XP_018058633.1,
		24	Rhagoletis_zephyria_snowberry_fruit_fly_Insecta_Tephritidae_XP_017470597.1,
		25	Drosophila_navojoa_flies_Insecta_Drosophilidae_XP_017955051.1,
		26	Acyrthosiphon_pisum_pea_aphid_Insecta_Aphididae_XP_008181512.1,
		27	Megachile_rotundata_alfalfa_leafcutting_bee_Insecta_Megachilidae_XP_012137041.1,
		28	Trachymyrmex_cornetzi_ants_Insecta_Formicidae_XP_018374266.1,
		29	Agrilus_planipennis_emerald_ash_borer_Insecta_Buprestidae_XP_018322526.1,
		30	Ctenocephalides_felis_cat_flea_Insecta_Pulicidae_XP_026472782.1,
		31	Anoplophora_glabripennis_Asian_longhorned_beetle_Insecta_Cerambycidae_XP_018576732.1,
		32	Anoplophora_glabripennis_Asian_longhorned_beetle_Insecta_Cerambycidae_XP_018576728.1,
		33	Drosophila_serrata_flies_Insecta_Drosophilidae_XP_020801661.1,
```

```
		34	Ooceraea_biroi_clonal_raider_ant_Insecta_Formicidae_XP_011352179.1,
		35	Apis_cerana_Asiatic_honeybee_Insecta_Apidae_XP_016915229.1,
		36	Hyposmocoma_kahamanoa_moths_Insecta_Cosmopterigidae_XP_026331265.1,
		37	Drosophila_rhopaloa_flies_Insecta_Drosophilidae_XP_016990292.1,
		38	Ceratina_calcarata_bees_Insecta_Apidae_XP_026675585.1,
		39	Melanaphis_sacchari_aphids_Insecta_Aphididae_XP_025205984.1,
		40	Nomia_melanderi_Alkali_bee_Insecta_Halictidae_XP_031839061.1,
		41	Drosophila_eugracilis_flies_Insecta_Drosophilidae_XP_017080359.1,
		42	Osmia_bicornis_bicornis_red_mason_bee_Insecta_Megachilidae_XP_029053011.1,
		43	Lucilia_cuprina_Australian_sheep_blowfly_Insecta_Calliphoridae_XP_023303769.1,
		44	Nicrophorus_vespilloides_beetles_Insecta_Silphidae_XP_017779772.1,
		45	Nicrophorus_vespilloides_beetles_Insecta_Silphidae_XP_017776178.1,
		46	Thrips_palmi_thrips_Insecta_Thripidae_XP_034233444.1,
		47	Bactrocera_latifrons_flies_Insecta_Tephritidae_XP_018795465.1,
		48	Bombyx_mori_domestic_silkworm_Insecta_Bombycidae_XP_004925143.1,
		49	Bradysia_coprophila_flies_Insecta_Sciaridae_XP_037046853.1,
		50	Zootermopsis_nevadensis_termites_Insecta_Termopsidae_XP_021938995.1,
		51	Eufriesea_mexicana_bees_Insecta_Apidae_XP_017764146.1,
		52	Bactrocera_dorsalis_oriental_fruit_fly_Insecta_Tephritidae_XP_011213805.1,
		53	Homo_sapiens_human_Dipnotetrapodomorpha_Hominidae_XP_005262724.1,
		54	Papilio_machaon_common_yellow_swallowtail_Insecta_Papilionidae_XP_014371186.1,
		55	Drosophila_miranda_flies_Insecta_Drosophilidae_XP_017135620.1,
		56	Ceratosolen_solmsi_marchali_wasps_ants_and_bees_Insecta_Agaonidae_XP_011504463.1,
		57	Temnothorax_curvispinosus_ants_Insecta_Formicidae_XP_024867521.1,
		58	Danaus_plexippus_plexippus_monarch_butterfly_Insecta_XP_032525972.1,
		59	Aedes_aegypti_yellow_fever_mosquito_Insecta_Culicidae_XP_001661518.1,
		60	Formica_exsecta_ants_Insecta_Formicidae_XP_029667472.1,
		61	Rhopalosiphum_maidis_corn_leaf_aphid_Insecta_Aphididae_XP_026816943.1,
		62	Ceratitis_capitata_Mediterranean_fruit_fly_Insecta_Tephritidae_XP_004525504.1,
		63	Drosophila_ananassae_flies_Insecta_Drosophilidae_XP_001958363.1,
		64	Bombus_bifarius_bees_Insecta_Apidae_XP_033318703.1,
		65	Athalia_rosae_coleseed_sawfly_Insecta_Tenthredinidae_XP_020708945.1,
		66	Drosophila_busckii_flies_Insecta_Drosophilidae_XP_017842574.1,
		67	Megalopta_genalis_bees_Insecta_Halictidae_XP_033329072.1,
		68	Nylanderia_fulva_ants_Insecta_Formicidae_XP_029164970.1,
		69	Bombus_impatiens_common_eastern_bumble_bee_Insecta_Apidae_XP_003485726.1,
		70	Linepithema_humile_Argentine_ant_Insecta_Formicidae_XP_012229075.1,
		71	Osmia_lignaria_orchard_mason_bee_Insecta_Megachilidae_XP_034195581.1,
		72	Belonocnema_treatae_wasps_ants_and_bees_Insecta_Cynipidae_XP_033231214.1,
		73	Amyelois_transitella_moths_Insecta_Pyralidae_XP_013195261.1,
		74	Bicyclus_anynana_squinting_bush_brown_Insecta_XP_023952108.1,
		75	Musca_domestica_house_fly_Insecta_Muscidae_XP_005191114.1,
		76	Bombus_vosnesenskii_bees_Insecta_Apidae_XP_033364229.1,
		77	Galleria_mellonella_greater_wax_moth_Insecta_Pyralidae_XP_026763549.1,
		78	Dufourea_novaeangliae_bees_Insecta_Halictidae_XP_015431079.1,
		79	Glossina_fuscipes_tsetse_fly_Insecta_Glossinidae_XP_037880144.1,
		80	Trachymyrmex_septentrionalis_ants_XP_018340687.1,
		81	Manduca_sexta_tobacco_hornworm_Insecta_Sphingidae_XP_030037441.2,
		82	Stomoxys_calcitrans_stable_fly_Insecta_Muscidae_XP_013114599.1,
		83	Dendroctonus_ponderosae_mountain_pine_beetle_Insecta_Curculionidae_XP_019766993.1,
		84	Wasmannia_auropunctata_little_fire_ant_Insecta_Formicidae_XP_011692078.1,
		85	Harpegnathos_saltator_Jerdon_s_jumping_ant_Insecta_Formicidae_XP_011153357.1,
		86	Contarinia_nasturtii_swede_midge_Insecta_Cecidomyiidae_XP_031631394.1,
		87	Drosophila_pseudoobscura_flies_XP_001354183.1,
		88	Bombus_terrestris_buff_tailed_bumblebee_Insecta_Apidae_XP_003402307.1,
		89	Monomorium_pharaonis_pharaoh_ant_Insecta_Formicidae_XP_036139285.1,
```

```
→→90→Hermetia_illucens_flies_Insecta_Stratiomyidae_XP_037907628.1,
→→91→Drosophila_persimilis_flies_Insecta_Drosophilidae_XP_026849516.1,
→→92→Vanessa_tameamea_butterflies_Insecta_XP_026496444.1,
→→93→Aphis_gossypii_cotton_aphid_Insecta_Aphididae_XP_027852657.1,
→→94→
Camponotus_floridanus_Florida_carpenter_ant_Insecta_Formicidae_XP_011264013.1,
→→95→
Bactrocera_tryoni_Queensland_fruit_fly_Insecta_Tephritidae_XP_039965029.1,
→→96→
Microplitis_demolitor_wasps_ants_and_bees_Insecta_Braconidae_XP_014300444.1,
→→97→Polistes_canadensis_wasps_ants_and_bees_Insecta_Vespidae_XP_014616649.1,
→→98→Photinus_pyralis_common_eastern_firefly_XP_031347802.1,
→→99→Zerene_cesonia_dogface_butterfly_Insecta_Pieridae_XP_038220977.1,
→→100→Orussus_abietinus_hymenopterans_Insecta_Orussidae_XP_012282071.1,
→→101→Folsomia_candida_springtails_Collembola_Isotomidae_XP_035702540.1,
→→102→Solenopsis_invicta_red_fire_ant_Insecta_Formicidae_XP_011156104.1,
→→103→Apis_florea_little_honeybee_Insecta_Apidae_XP_012340198.1,
→→104→Diachasma_alloeum_wasps_ants_and_bees_Insecta_Braconidae_XP_015126514.1,
→→105→Nilaparvata_lugens_brown_planthopper_Insecta_Delphacidae_XP_039287508.1,
→→106→Nilaparvata_lugens_brown_planthopper_Insecta_Delphacidae_XP_039287723.1,
→→107→Trichoplusia_ni_cabbage_looper_Insecta_XP_026746668.1,
→→108→Helicoverpa_armigera_cotton_bollworm_Insecta_XP_021199365.1,
→→109→Bactrocera_oleae_olive_fruit_fly_Insecta_Tephritidae_XP_014094726.1,
→→110→Mus_musculus_house_mouse_Dipnotetrapodomorpha_Muridae_XP_006503925.1,
→→111→Onthophagus_taurus_beetles_Insecta_Scarabaeidae_XP_022918072.1,
→→112→Myzus_persicae_green_peach_aphid_Insecta_Aphididae_XP_022182046.1,
→→113→Dinoponera_quadriceps_ants_Insecta_Formicidae_XP_014471283.1,
→→114→Aethina_tumida_small_hive_beetle_Insecta_XP_019878451.1,
→→115→Bemisia_tabaci_sweet_potato_whitefly_Insecta_Aleyrodidae_XP_018908293.1,
→→116→Nasonia_vitripennis_jewel_wasp_Insecta_Pteromalidae_XP_001599700.1,
→→117→Cephus_cinctus_wheat_stem_sawfly_Insecta_Cephidae_XP_015594969.1,
→→118→Drosophila_mojavensis_flies_Insecta_Drosophilidae_XP_002007480.1,
→→119→Sipha_flava_yellow_sugarcane_aphid_Insecta_Aphididae_XP_025422359.1,
→→120→Trachymyrmex_zeteki_ants_Insecta_Formicidae_XP_018309379.1,
→→121→Drosophila_grimshawi_flies_Insecta_Drosophilidae_XP_001983810.1,
→→122→
Tribolium_castaneum_red_flour_beetle_Insecta_Tenebrionidae_XP_008190526.1,
→→123→Habropoda_laboriosa_bees_Insecta_Apidae_XP_017797480.1,
→→124→Polistes_dominula_European_paper_wasp_Insecta_Vespidae_XP_015185360.1,
→→125→Papilio_polytes_common_Mormon_Insecta_Papilionidae_XP_013135877.1,
→→126→Zeugodacus_cucurbitae_melon_fly_Insecta_Tephritidae_XP_011196038.1,
→→127→
Lucilia_sericata_common_green_bottle_fly_Insecta_Calliphoridae_XP_037819406.1,
→→128→Drosophila_arizonae_flies_Insecta_Drosophilidae_XP_017862575.1,
→→129→Bombyx_mandarina_wild_silkworm_Insecta_Bombycidae_XP_028037540.1,
→→130→
Acromyrmex_echinatior_Panamanian_leafcutter_ant_Insecta_Formicidae_XP_01106016
5.1,
→→131→Drosophila_willistoni_flies_Insecta_Drosophilidae_XP_002061809.1,
→→132→Aedes_albopictus_Asian_tiger_mosquito_Insecta_Culicidae_XP_029718184.1,
→→133→Aedes_albopictus_Asian_tiger_mosquito_Insecta_Culicidae_XP_029735770.1,
→→134→Aedes_albopictus_Asian_tiger_mosquito_Insecta_Culicidae_XP_029735681.1,
→→135→Odontomachus_brunneus_ants_Insecta_Formicidae_XP_032666296.1,
→→136→Pararge_aegeria_specked_wood_butterfly_Insecta_XP_039762194.1,
→→137→Drosophila_virilis_flies_Insecta_Drosophilidae_XP_002046857.1
→→;
  [Note: This tree contains information on the topology,
         branch lengths (if present), and the probability
         of the partition indicated by the branch.]
  tree con_50_majrule =
(1:0.03692356,(((((((((((((((((((2:0.3385985,3:0.293406)1.000:0.6752865,114:0.50373
15)1.000:0.1241832,((8:0.3290839,83:0.3479445)1.000:0.569096,(31:0.02956691,32:0.07
864367)1.000:0.4078902)0.960:0.08958575)1.000:0.08982005,(29:0.5588426,98:0.5567371
)0.825:0.08958182,(44:0.01400179,45:0.02641081)1.000:0.5268233,111:0.4533959,122:0.
5620655)1.000:0.3210019,(((14:0.7474366,(((((26:0.08014922,112:0.05233326)0.996:0.0
2525834,((39:0.06077687,93:0.05985045)0.760:0.004421319,61:0.04988905)1.000:0.03536
272)1.000:0.1588025,119:0.2072927)1.000:0.8954535,((53:0.0716706,110:0.1316716)1.00
0:1.431242,101:0.9447711)1.000:0.2270254)1.000:0.1441367,115:0.737839)0.578:0.07421
351)0.833:0.1085487,(105:0.01776463,106:0.005163987)1.000:0.5163667)0.747:0.0644646
4,(46:0.6612425,50:0.4963945)0.814:0.1449412)0.959:0.07237422)0.771:0.0407427,((((4
:0.2358679,104:0.1195539)1.000:0.3250175,96:0.5154778)1.000:0.1744763,(((((((((6:0.
```

```
243994,70:0.1754986)0.810:0.0281212,((60:0.04550222,94:0.08906989)0.632:0.01120698,
68:0.0906188)1.000:0.06654652)0.592:0.01958472,((((7:0.06298295,(((((9:7.058738E-4,
23:0.00380274)1.000:0.01311193,130:0.02288073)0.591:0.002373802,80:0.02147711)0.540
:0.001767949,28:0.01743221)1.000:0.01273898,120:0.02933363)1.000:0.02147514)1.000:0
.08421167,84:0.08397329)0.969:0.01396276,(16:0.08475489,57:0.05935717)1.000:0.02884
883)0.771:0.01087306,(89:0.1144907,102:0.05692636)1.000:0.0329378)1.000:0.08636822)
0.861:0.02765022,34:0.1724014)1.000:0.1001842,(85:0.06190392,113:0.07775793,135:0.0
9074688)1.000:0.07863221)1.000:0.1313127,((10:0.08675361,(97:0.02458195,124:0.03965
07)1.000:0.07933525)1.000:0.3655194,((((((15:0.07766408,(35:0.02995715,103:0.032351
71)0.831:0.02614608)1.000:0.1230671,(51:0.1862159,(((64:0.002785792,76:0.002659411)
0.572:0.001397852,69:0.004429903)1.000:0.01695457,88:0.008708067)1.000:0.1280484)0.
945:0.02495527)0.949:0.0226802,38:0.1560511)0.992:0.02878322,123:0.1081221)1.000:0.
04060645,(27:0.09978085,(42:0.008273381,71:0.009408856)1.000:0.04608062)1.000:0.107
6897)1.000:0.04059924,((40:0.122489,67:0.07891815)1.000:0.03764658,78:0.1327597)1.0
00:0.1016072)1.000:0.0750994)0.995:0.06104414)1.000:0.09413724,((56:0.2218575,116:0
.2182908)1.000:0.184072,72:0.6342481)0.946:0.0473391)0.745:0.05360006,100:0.3927008
)0.845:0.06631715,117:0.5843595)0.614:0.0569009)0.847:0.07992031,65:0.3748611)1.000
:0.2306411)0.787:0.0954987,((((((((((12:0.2561297,136:0.1998334)0.846:0.06897806,74:
0.2391701)1.000:0.13116,(58:0.3269345,92:0.2810562)0.955:0.07980204)1.000:0.0794526
2,(22:0.30761,99:0.2862215)1.000:0.1258191)0.964:0.03710247,((13:0.06929902,54:0.06
568036)0.997:0.05205069,125:0.1370471)1.000:0.2639616)0.930:0.04151969,((48:0.00401
2966,129:0.01835151)1.000:0.3547686,81:0.2953836)1.000:0.1353375)1.000:0.07529146,(
((19:0.01814714,20:0.0452565)1.000:0.2229991,107:0.255456)0.732:0.07009797,108:0.22
21617)1.000:0.1200762)0.816:0.0501896,73:0.3889063)0.825:0.07301041,36:0.3561286,77
:0.311985)1.000:0.55625)1.000:0.1491125,30:0.6279218)1.000:0.1581391,(49:0.5455672,
86:0.5240222)1.000:0.1561332)0.999:0.08367173,((59:0.1039302,(132:0.006966458,(133:
0.003007809,134:0.01912927)0.964:0.008773347)1.000:0.1041229)1.000:0.4890282,90:0.3
700946)0.967:0.09405811)1.000:0.2453955,5:0.3385598)0.639:0.06510739,(((24:0.178556
8,((((47:0.02730259,52:0.01989886)0.963:0.005800408,95:0.009840547)1.000:0.03249193
,109:0.03383118)1.000:0.03225733,126:0.06320642)1.000:0.09453505)0.972:0.05318888,6
2:0.10299)1.000:0.1599377,(((43:0.02921961,127:0.04251379)1.000:0.1528446,79:0.3757
697)0.706:0.07505774,(75:0.1428764,82:0.2036)1.000:0.05603843)1.000:0.1150453)0.684
:0.04930031)1.000:0.2149133,21:0.1546157)1.000:0.0885739,131:0.2578737)0.599:0.0440
1018,((((17:0.06491301,(37:0.07407994,41:0.1171101)1.000:0.03673922)1.000:0.0234109
,63:0.1249566)0.810:0.02439695,33:0.1548755)1.000:0.04745553,(55:0.001307877,(87:0.
001672227,91:0.001556852)1.000:0.006562441)1.000:0.149112)1.000:0.07437489)0.998:0.
03347521,66:0.2188834)0.590:0.01559439,11:0.1326561)0.968:0.02501069,121:0.09970248
)1.000:0.04296224,(18:0.02465085,137:0.01305957)1.000:0.0661759)1.000:0.05785041,(2
5:0.04380833,(118:0.00667083,128:0.008087793)0.992:0.009207734)1.000:0.06308023);LF
LF
   [Note: This tree contains information only on the topologyLF
          and branch lengths (median of the posterior probability density).]LF
   tree con_50_majrule = 
(1:0.03692356,((((((((((((((((((2:0.3385985,3:0.293406):0.6752865,114:0.5037315):0
.1241832,((8:0.3290839,83:0.3479445):0.569096,(31:0.02956691,32:0.07864367):0.40789
02):0.08958575):0.08982005,(29:0.5588426,98:0.5567371):0.08958182,(44:0.01400179,45
:0.02641081):0.5268233,111:0.4533959,122:0.5620655):0.3210019,(((14:0.7474366,(((((
26:0.08014922,112:0.05233326):0.02525834,((39:0.06077687,93:0.05985045):0.004421319
,61:0.04988905):0.03536272):0.1588025,119:0.2072927):0.8954535,((53:0.0716706,110:0
.1316716):1.431242,101:0.9447711):0.2270254):0.1441367,115:0.737839):0.07421351):0.
1085487,(105:0.01776463,106:0.005163987):0.5163667):0.06446464,(46:0.6612425,50:0.4
963945):0.1449412):0.07237422):0.0407427,((((4:0.2358679,104:0.1195539):0.3250175,9
6:0.5154778):0.1744763,(((((((((6:0.243994,70:0.1754986):0.0281212,((60:0.04550222,
94:0.08906989):0.01120698,68:0.0906188):0.06654652):0.01958472,((((7:0.06298295,(((
((9:7.058738E-4,23:0.00380274):0.01311193,130:0.02288073):0.002373802,80:0.02147711
):0.001767949,28:0.01743221):0.01273898,120:0.02933363):0.02147514):0.08421167,84:0
.08397329):0.01396276,(16:0.08475489,57:0.05935717):0.02884883):0.01087306,(89:0.11
44907,102:0.05692636):0.0329378):0.08636822):0.02765022,34:0.1724014):0.1001842,(85
:0.06190392,113:0.07775793,135:0.09074688):0.07863221):0.1313127,((10:0.08675361,(9
7:0.02458195,124:0.0396507):0.07933525):0.3655194,((((((15:0.07766408,(35:0.0299571
5,103:0.03235171):0.02614608):0.1230671,(51:0.1862159,(((64:0.002785792,76:0.002659
411):0.001397852,69:0.004429903):0.01695457,88:0.008708067):0.1280484):0.02495527):
0.0226802,38:0.1560511):0.02878322,123:0.1081221):0.04060645,(27:0.09978085,(42:0.0
08273381,71:0.009408856):0.04608062):0.1076897):0.04059924,((40:0.122489,67:0.07891
815):0.03764658,78:0.1327597):0.1016072):0.0750994):0.06104414):0.09413724,((56:0.2
218575,116:0.2182908):0.184072,72:0.6342481):0.0473391):0.05360006,100:0.3927008):0
.06631715,117:0.5843595):0.0569009):0.07992031,65:0.3748611):0.2306411):0.0954987,(
((((((((12:0.2561297,136:0.1998334):0.06897806,74:0.2391701):0.13116,(58:0.3269345,
92:0.2810562):0.07980204):0.07945262,(22:0.30761,99:0.2862215):0.1258191):0.0371024
7,((13:0.06929902,54:0.06568036):0.05205069,125:0.1370471):0.2639616):0.04151969,((
48:0.004012966,129:0.01835151):0.3547686,81:0.2953836):0.1353375):0.07529146,(((19:
0.01814714,20:0.0452565):0.2229991,107:0.255456):0.07009797,108:0.2221617):0.120076
2):0.0501896,73:0.3889063):0.07301041,36:0.3561286,77:0.311985):0.55625):0.1491125,
```

```
30:0.6279218):0.1581391,(49:0.5455672,86:0.5240222):0.1561332):0.08367173,((59:0.10
39302,(132:0.006966458,(133:0.003007809,134:0.01912927):0.008773347):0.1041229):0.4
890282,90:0.3700946):0.09405811):0.2453955,5:0.3385598):0.06510739,(((24:0.1785568,
((((47:0.02730259,52:0.01989886):0.005800408,95:0.009840547):0.03249193,109:0.03383
118):0.03225733,126:0.06320642):0.09453505):0.05318888,62:0.10299):0.1599377,(((43:
0.02921961,127:0.04251379):0.1528446,79:0.3757697):0.07505774,(75:0.1428764,82:0.20
36):0.05603843):0.1150453):0.04930031):0.2149133,21:0.1546157):0.0885739,131:0.2578
737):0.04401018,((((17:0.06491301,(37:0.07407994,41:0.1171101):0.03673922):0.023410
9,63:0.1249566):0.02439695,33:0.1548755):0.04745553,(55:0.001307877,(87:0.001672227
,91:0.001556852):0.006562441):0.149112):0.07437489):0.03347521,66:0.2188834):0.0155
9439,11:0.1326561):0.02501069,121:0.09970248):0.04296224,(18:0.02465085,137:0.01305
957):0.0661759):0.05785041,(25:0.04380833,(118:0.00667083,128:0.008087793):0.009207
734):0.06308023);LF
end;LF
```
