# Supplementary material for: Inferences on the evolution of the ascorbic acid synthesis pathway in insects using Phylogenetic Tree Collapser (PTC), a tool for the automated collapsing of phylogenetic trees using taxonomic information
Source: J Integr Bioinform. 2024 Jul 24;21(2):20230051. doi: 10.1515/jib-2023-0051 (PMC11377030; doi:10.1515/jib-2023-0051)
Supplement: Supplementary file 1 — Supplementary Material Details [file j_jib-2023-0051_suppl_001.zip › Supplementary_File_9_UGDH_Sec.con_PDF.pdf]

```
#NEXUS
[ID: 7209653742]
begin taxa;
	dimensions ntax=36;
	taxlabels
		Drosophila_yakuba_flies_Insecta_Drosophilidae_XP_002093972.1
		Trichogramma_pretiosum_wasps_ants_and_bees_Insecta_Trichogrammatidae_XP_014220926.1
		Drosophila_melanogaster_fruit_fly_Insecta_Drosophilidae_NP_476980.1
		Drosophila_sechellia_flies_Insecta_Drosophilidae_XP_002035633.1
		Anopheles_coluzzii_mosquitos_Insecta_Culicidae_XP_040223207.1
		Drosophila_mauritiana_flies_Insecta_Drosophilidae_XP_033160592.1
		Anopheles_albimanus_mosquitos_Insecta_Culicidae_XP_035791595.1
		Drosophila_biarmipes_flies_Insecta_Drosophilidae_XP_016955928.1
		Drosophila_erecta_flies_Insecta_Drosophilidae_XP_001971522.1
		Drosophila_simulans_flies_Insecta_Drosophilidae_XP_002083909.1
		Drosophila_suzukii_flies_Insecta_Drosophilidae_XP_016934781.1
		Copidosoma_floridanum_wasps_ants_and_bees_Insecta_Encyrtidae_XP_014220016.1
		Anopheles_stephensi_Asian_malaria_mosquito_Insecta_Culicidae_XP_035907081.1
		Pediculus_humanus_corporis_human_body_louse_Insecta_Pediculidae_XP_002431407.1
		Drosophila_takahashii_flies_Insecta_Drosophilidae_XP_017010632.1
		Chelonus_insularis_wasps_ants_and_bees_Insecta_Braconidae_XP_034936581.1
		Drosophila_subpulchrella_flies_Insecta_Drosophilidae_XP_037720499.1
		Drosophila_subobscura_flies_Insecta_Drosophilidae_XP_034655354.1
		Anopheles_arabiensis_mosquitos_Insecta_Culicidae_XP_040153167.1
		Drosophila_elegans_flies_Insecta_Drosophilidae_XP_017122169.1
		Drosophila_obscura_flies_Insecta_Drosophilidae_XP_022212256.1
		Drosophila_ficusphila_flies_Insecta_Drosophilidae_XP_017048488.1
		Culex_pipiens_pallens_northern_house_mosquito_Insecta_Culicidae_XP_039444559.1
		Drosophila_bipectinata_flies_Insecta_Drosophilidae_XP_017095299.1
		Drosophila_kikkawai_flies_Insecta_Drosophilidae_XP_017028811.1
		Drosophila_santomea_flies_Insecta_Drosophilidae_XP_039485717.1
		Anopheles_gambiae_str._PEST_African_malaria_mosquito_Insecta_Culicidae_XP_316568.4
		Drosophila_guanche_flies_Insecta_Drosophilidae_XP_034134195.1
		Culex_quinquefasciatus_southern_house_mosquito_Insecta_Culicidae_XP_038120149.1
		Homo_sapiens_human_Dipnotetrapodomorpha_Hominidae_XP_005262724.1
		Bombyx_mori_domestic_silkworm_Insecta_Bombycidae_XP_004925143.1
		Atta_colombica_ants_Insecta_Formicidae_XP_018058633.1
		Nicrophorus_vespilloides_beetles_Insecta_Silphidae_XP_017779772.1
		Mus_musculus_house_mouse_Dipnotetrapodomorpha_Muridae_XP_006503925.1
		Rhopalosiphum_maidis_corn_leaf_aphid_Insecta_Aphididae_XP_026816943.1
		Folsomia_candida_springtails_Collembola_Isotomidae_XP_035702540.1
	;
end;
begin trees;
	translate
		1 Drosophila_yakuba_flies_Insecta_Drosophilidae_XP_002093972.1,
		2 Trichogramma_pretiosum_wasps_ants_and_bees_Insecta_Trichogrammatidae_XP_014220926.1,
		3 Drosophila_melanogaster_fruit_fly_Insecta_Drosophilidae_NP_476980.1,
		4 Drosophila_sechellia_flies_Insecta_Drosophilidae_XP_002035633.1,
		5 Anopheles_coluzzii_mosquitos_Insecta_Culicidae_XP_040223207.1,
		6 Drosophila_mauritiana_flies_Insecta_Drosophilidae_XP_033160592.1,
		7 Anopheles_albimanus_mosquitos_Insecta_Culicidae_XP_035791595.1,
		8 Drosophila_biarmipes_flies_Insecta_Drosophilidae_XP_016955928.1,
		9 Drosophila_erecta_flies_Insecta_Drosophilidae_XP_001971522.1,
		10 Drosophila_simulans_flies_Insecta_Drosophilidae_XP_002083909.1,
		11 Drosophila_suzukii_flies_Insecta_Drosophilidae_XP_016934781.1,
		12 Copidosoma_floridanum_wasps_ants_and_bees_Insecta_Encyrtidae_XP_014220016.1,
		13 Anopheles_stephensi_Asian_malaria_mosquito_Insecta_Culicidae_XP_035907081.1,
```

```
	14→
		Pediculus_humanus_corporis_human_body_louse_Insecta_Pediculidae_XP_002431407.1
		,LF
	15→Drosophila_takahashii_flies_Insecta_Drosophilidae_XP_017010632.1,LF
	16→
		Chelonus_insularis_wasps_ants_and_bees_Insecta_Braconidae_XP_034936581.1,LF
	17→Drosophila_subpulchrella_flies_Insecta_Drosophilidae_XP_037720499.1,LF
	18→Drosophila_subobscura_flies_Insecta_Drosophilidae_XP_034655354.1,LF
	19→Anopheles_arabiensis_mosquitos_Insecta_Culicidae_XP_040153167.1,LF
	20→Drosophila_elegans_flies_Insecta_Drosophilidae_XP_017122169.1,LF
	21→Drosophila_obscura_flies_Insecta_Drosophilidae_XP_022212256.1,LF
	22→Drosophila_ficusphila_flies_Insecta_Drosophilidae_XP_017048488.1,LF
	23→
		Culex_pipiens_pallens_northern_house_mosquito_Insecta_Culicidae_XP_039444559.1
		,LF
	24→Drosophila_bipectinata_flies_Insecta_Drosophilidae_XP_017095299.1,LF
	25→Drosophila_kikkawai_flies_Insecta_Drosophilidae_XP_017028811.1,LF
	26→Drosophila_santomea_flies_Insecta_Drosophilidae_XP_039485717.1,LF
	27→
		Anopheles_gambiae_str._PEST_African_malaria_mosquito_Insecta_Culicidae_XP_3165
		68.4,LF
	28→Drosophila_guanche_flies_Insecta_Drosophilidae_XP_034134195.1,LF
	29→
		Culex_quinquefasciatus_southern_house_mosquito_Insecta_Culicidae_XP_038120149.
		1,LF
	30→Homo_sapiens_human_Dipnotetrapodomorpha_Hominidae_XP_005262724.1,LF
	31→Bombyx_mori_domestic_silkworm_Insecta_Bombycidae_XP_004925143.1,LF
	32→Atta_colombica_ants_Insecta_Formicidae_XP_018058633.1,LF
	33→Nicrophorus_vespilloides_beetles_Insecta_Silphidae_XP_017779772.1,LF
	34→Mus_musculus_house_mouse_Dipnotetrapodomorpha_Muridae_XP_006503925.1,LF
	35→Rhopalosiphum_maidis_corn_leaf_aphid_Insecta_Aphididae_XP_026816943.1,LF
	36→Folsomia_candida_springtails_Collembola_Isotomidae_XP_035702540.1LF
	;LF
    [Note: This tree contains information on the topology, LF
          branch lengths (if present), and the probabilityLF
          of the partition indicated by the branch.]LF
    tree con_50_majrule =
    (1:0.01850179,26:0.02194616,(((((((((2:0.3007357,12:0.5343914)0.936:0.08666136,((((
    (14:0.7833255,((30:0.05683526,34:0.1878029)1.000:1.391394,36:1.067316)0.982:0.25555
    25,35:1.021118)1.000:0.2993398,16:0.3907344)0.994:0.3740395,32:0.3858501)0.993:0.34
    94596,31:1.086799)0.665:0.1383966,33:0.818676)1.000:0.3066795)1.000:0.3507672,((((5
    :0.01222659,(19:0.01369659,27:0.01208613)0.550:0.00497726)1.000:0.09961245,13:0.131
    3944)1.000:0.09541765,7:0.1626431)1.000:0.1229706,(23:0.007134079,29:0.0448917)1.00
    0:0.2026903)1.000:0.1718348)1.000:0.2326878,((18:0.02375139,28:0.02058305)1.000:0.0
    730963,21:0.07183998)1.000:0.1022416)0.998:0.04937023,24:0.1607121)0.522:0.02291348
    ,25:0.1927493)1.000:0.03632624,(((8:0.05030815,(11:0.03320594,17:0.03359624)1.000:0
    .02303905)1.000:0.03067871,15:0.06302282)0.954:0.01787377,(20:0.08449882,22:0.10096
    77)1.000:0.02504525)1.000:0.02339008)1.000:0.04075455,(3:0.03482461,((4:0.004672472
    ,10:0.0100617)0.996:0.007021822,6:0.01566016)0.997:0.007907641)1.000:0.03494902)1.0
    00:0.02290475,9:0.03827001)1.000:0.02455343);LF
LF
    [Note: This tree contains information only on the topologyLF
          and branch lengths (median of the posterior probability density).]LF
    tree con_50_majrule =
    (1:0.01850179,26:0.02194616,(((((((((2:0.3007357,12:0.5343914):0.08666136,(((((14:0
    .7833255,((30:0.05683526,34:0.1878029):1.391394,36:1.067316):0.2555525,35:1.021118)
    :0.2993398,16:0.3907344):0.3740395,32:0.3858501):0.3494596,31:1.086799):0.1383966,3
    3:0.818676):0.3066795):0.3507672,((((5:0.01222659,(19:0.01369659,27:0.01208613):0.0
    0497726):0.09961245,13:0.1313944):0.09541765,7:0.1626431):0.1229706,(23:0.007134079
    ,29:0.0448917):0.2026903):0.1718348):0.2326878,((18:0.02375139,28:0.02058305):0.073
    0963,21:0.07183998):0.1022416):0.04937023,24:0.1607121):0.02291348,25:0.1927493):0.
    03632624,(((8:0.05030815,(11:0.03320594,17:0.03359624):0.02303905):0.03067871,15:0.
    06302282):0.01787377,(20:0.08449882,22:0.1009677):0.02504525):0.02339008):0.0407545
    5,(3:0.03482461,((4:0.004672472,10:0.0100617):0.007021822,6:0.01566016):0.007907641
    ):0.03494902):0.02290475,9:0.03827001):0.02455343);LF
end;LF
```
